# ﻿Notes on the taxonomic status and distribution of some Cylindrotomidae (Diptera, Tipuloidea), with emphasis on Japanese species

**DOI:** 10.3897/zookeys.1083.75624

**Published:** 2022-01-24

**Authors:** Levente-Péter Kolcsár, Nikolai Paramonov, Yume Imada, Daichi Kato, Maribet Gamboa, Dai Shinoka, Makoto Kato, Kozo Watanabe

**Affiliations:** 1 Center for Marine Environmental Studies (CMES), Ehime University, Matsuyama, Ehime 790-8577, Japan; 2 Zoological Institute, Russian Academy of Sciences, 1 Universitetskaya Emb., St Petersburg 199034, Russia; 3 Graduate School of Science and Engineering, Ehime University, 2–5 Bunkyo-cho, Matsuyama, Ehime, 790-8577 Japan; 4 Echigo-Matsunoyama Museum of Natural Sciences, ‘Kyororo’, 1712-2 Matsunoyama, Tôkamachi, 942-1411, Japan; 5 Department of Ecology, Faculty of Sciences, Universidad Católica de la Santísima Concepción, 409054 Concepción, Chile; 6 Graduate School of Human and Environmental Studies, Kyoto University, Sakyo-ku, Yoshida-nihonmatsu-cho, Kyoto, 606-8501 Japan

**Keywords:** Barcode, COI sequences, comparison, Cylindrotominae, ovipositor, terminalia, Tipulomorpha

## Abstract

A morphological and molecular study of 17 Cylindrotomidae species revealed that the two subspecies of *Cylindrotomadistinctissima*, the Nearctic *C.americana* Osten Sacken, 1865, **stat. reval.** and the Palearctic *C.distinctissima* (Meigen, 1818), represent separated lineages and consequently are raised to species level. *Cylindrotomajaponica* Alexander, 1919, **syn. nov**. and *C.distinctissimaalpestris* Peus, 1952, **syn. nov.** are now known to be junior synonyms of *C.distinctissima*. *Triogmakuwanailimbinervis* Alexander, 1953, **syn. nov.** and *T.nimbipennis* Alexander, 1941, **syn. nov.** are now placed into synonymy under *Triogmakuwanai* (Alexander, 1913). The Japanese Cylindrotomidae are all redescribed and all available literature and distribution data are summarised. Supplementary descriptions and illustrations for male and female terminalia of *Cylindrotomanigriventris* Loew, 1849, *Diogmadmitrii* Paramonov, 2005, *Liogmanodicornis* (Osten Sacken, 1865), *Phalacrocerareplicata* (Linnaeus, 1758), *P.tipulina* Osten Sacken, 1865, and *Triogmatrisulcata* (Schummel, 1829) are provided. The following new distribution records are outlined; *Diogmacaudata* Takahashi, 1960 from Arkhangelsk Oblast, Russia; *D.glabrata* (Meigen, 1818) from Belarus, Latvia, and Altai Republic, Amur Oblast, Novgorod Oblast, Magadan Oblast, Samara Oblast, and Kuril Islands (Shikotan I and Paramushir I) in Russia; *Liogmaserraticornis* Alexander, 1919 from Khabarovsk Krai, Russia; *Phalacrocerareplicata* from Khabarovsk Krai, Russia; and the presence of *Cylindrotomanigriventris* in Altai Republic, Russia is confirmed.

## ﻿Introduction

The Cylindrotomidae, the so-called long-bodied crane flies, are the smallest crane fly family within the superfamily Tipuloidea, with 70 extant species and 18 extinct species (Greenwalt et al. 2019; Krzemiński et al. 2019; Kania-Kłosok et al. 2021; Oosterbroek 2021). The family is subdivided into two subfamilies, the Cylindrotominae (50 extant spp.) which are distributed in the Northern Hemisphere, and the Stibadocerinae (20 spp.) which occur in the Oriental, Australasian, and Neotropical Regions (Oosterbroek 2021).

The Cylindrotominae are characterised by the following character combinations: (head) 16-segmented antennae; (thorax) the transverse V-shaped suture of the scutum is less apparent than other crane flies; (abdomen) this is slender and elongated; (male terminalia) unbranched gonostylus; large aedeagal complex with trifid or secondary bifid (*Diogma* Edwards, 1938) aedeagus; relatively short and broad female terminalia with leaf- or blade-like cerci and hypogynial valves (Alexander 1928; Peus 1952; Brodo 1967; Ribeiro 2009). Although Cylindrotomidae are also characterised by reduction of radial wing veins (i.e., R_1_ and R_3_), this character is highly variable among species and specimens (Peus 1952; Brodo 1967). Cylindrotominae larvae are very distinctive and resemble parts of lower plants such as bryophytes to a remarkable degree, due to the following trait complexes: elongated cuticular outgrowths, body colourations (green to brown) and dorsal patterns (Alexander 1920; 1928; Peus 1952; Takahashi 1960; Brodo 1967; Imada 2020). The biology and morphology at the immature stages, with ecomorphological analyses of the elongated lobes are recently detailed for 11 species in five genera (Imada 2020).

Members of the subfamily Stibadocerinae are primarily separated from the Cylindrotominae based on the following characters in adults: very elongated antenna, usually longer than their entire body, and highly reduced number of wing veins, particularly, the lack of vein R_4+5_ (Ribeiro 2009).

Despite the low species diversity of Cylindrotominae, both genus- and species-level taxonomy are still problematic areas. Most of the Eastern Palearctic and Oriental species were originally described based upon characteristics of wing venation and body colouration (see species descriptions of C.P. Alexander). Later revisions of European and Nearctic Cylindrotomidae revealed that these characters were highly variable among specimens (Peus 1952; Brodo 1967). The monophyly and validity of the different genera as *Cylindrotoma*, *Liogma*, and *Phalacrocera*, and the systematic position of several Eastern Palearctic and Oriental species have been in question for some time (Peus 1952; Takahashi 1960; Brodo 1967).

This article clarifies taxonomic status of some Cylindrotominae at species level, based on morphological comparison and molecular (mtDNA COI) data. The species that occur in Japan are redescribed, including the review of the species’ literature and distribution data. An elevation of a subspecies and new synonyms are proposed. The genus-level taxonomy and species classification will be presented in the future with the phylogeny of the Cylindrotominae.

## ﻿Materials and methods

A total of 456 Cylindrotominae specimens belonging to 17 taxa of five genera was investigated. The specimens were identified in reference to the original literature (Peus 1952; Takahashi 1960; Brodo 1967; Nakamura 2001; Paramonov 2006) and by comparing with type specimens. Terminology follows Cumming and Wood (2017). For preparation of male and female terminalia, caudal end of abdomen was cut off and macerated with 10–15% KOH at room temperature and neutralised with 3% acetic acid; then the terminalia was placed in glycerol and observed under a stereomicroscope. The cleared terminalia was preserved in tubes containing glycerol. Wings and entire bodies of specimens were photographed using a Zeiss Stemi 508 stereomicroscope equipped with Canon Kiss M digital camera; the photos were stacked using the Zerene Stacker version 1.04. Illustrations were drawn with Adobe Photoshop CC 2019. For providing distribution maps, an approximate spatial coordinate was selected on Google Earth Pro and with QGIS version 3.6 Noosa for each sampling locality in literature.

Specimens from the following depositories were examined:

**BLKU**Biosystematics Laboratory, Kyushu University.

**CKLP** Private Collection of L.-P. Kolcsár.

**CYI** Private Collection of Y. Imada.

**EUMJ**Ehime University Museum, Matsuyama, Japan.

**FAUK** Entomological Laboratory, Faculty of Agriculture, Kyushu University.

**LMM** Regional Museum of Lapland, Rovaniemi, Finland.

**ZIN**Zoological Institute, Russian Academy of Science, Saint-Petersburg, Russia.

**ZFMK**Zoological Research Museum Alexander Koenig, Bonn, Germany.

**USNM**U.S. National Museum of Natural History, Smithsonian Institution, Washington, D.C., USA.

### ﻿DNA isolation, amplification, sequencing, and alignment

Mitochondrial DNA was extracted using DNeasy Blood & Tissue kits (Qiagen GmbH, Hilden, Germany). Extracted DNA was amplified using LCO-1490 and HCO-2198 primers (Folmer et al. 1994) on the 658 bp region of the mitochondrial cytochrome oxidase I (COI, cox1) gene, with an annealing temperature of 38⁰C and 40 PCR cycles. The PCR products were purified using the QIAquick PCR Purification Kit (Qiagen GmbH, Hilden, Germany) and sequenced by Eurofins Operon (Tokyo, Japan) in both directions using the same primer set as above. Forward and reverse reads were assembled and edited using CodonCode Aligner v 3.5 (Codon Code Corporation, Dedham, USA). All sequences were submitted to GenBank, and also transferred to BoldSystems. BoldSystems ID was used as sequence identifier.

The newly sequenced (for this study) and published (public) Cylindrotominae sequences from BoldSystems (http://www.boldsystems.org) (Table 1) in multiple COI alignments were used in this study. All sequences were aligned using ClustalW (Thompson et al. 1994) and the phylogenetic search was conducted using a maximum likelihood approach in PhyML v 3.0 (Guindon and Gascuel 2003) under a GTR model of evolution (as determined by Modeltest v 3.7; Posada and Crandall 1998) and 1000 bootstrap analysis. Specimens of Limoniidae: *Limoniaphragmitidis* (Schrank, 1781) (FINTI876-12), Pediciidae: *Pediciarivosa* (Linnaeus, 1758) (FINTI657-12), and Tipulidae: *Tipulamaxima* Poda, 1761 (FINTI636-12) were used as outgroups. The genetic distance between species groups was determined using DnaSP v 5.1 (Librado and Rozas 2009).

**Table 1.** Barcode sequences included this study.

Species name – BoldSystems	Species name (new)	Genebank ID	BOLD ID	BOLD BIN	country	latitude	longitude	date	collectors
**New sequences for this study**									
*Cylindrotomad.distinctissima*	*Cylindrotomadistinctissima*	MT151834	GBMNB25014-20	BOLD:AAD0770	Finland	63.92	26.869	2008/6/18-7/13	J. Salmela
*Cylindrotomajaponica*	*Cylindrotomadistinctissima*	MT151788	GBMNB24968-20	BOLD:AAD0770	Japan	39.94	140.86	2014.09.20	D. Kato
		MT151789	GBMNB24969-20	BOLD:AAD0770	Japan	35.74	139.18	2019.05.11	L.P. Kolcsár
		MT151790	GBMNB24970-20	BOLD:AAD0770	Japan	43.65	142.82	2019.07.24	L.P. Kolcsár
		MT151791	GBMNB24971-20	BOLD:AAD0770	Japan	43.39	143.96	2019.07.27	L.P. Kolcsár
		MT151805	GBMNB24985-20	BOLD:AAD0770	Japan	35.32	133.59	2015.05.17	D. Kato
		MT151806	GBMNB24986-20	BOLD:AAD0770	Japan	40.5	140.2	2013.09.18	D. Kato
		MT151807	GBMNB24987-20	BOLD:AAD0770	Japan	36.11	137.56	2016.07.22	D. Kato
*Cylindrotomanigriventris*		MT151830	GBMNB25010-20	BOLD:ABV9491	Finland	60.6	23.959	2018.06.09	Kato D.
		MT151826	GBMNB25006-20	BOLD:AED8500	Russia	51.06	85.59	2016/06/27-30	N.E. Vikhrev
*Diogmadmitrii*		MT151827	GBMNB25007-20	BOLD:AED6086	Russia	44	39.994	2012.06.11	N.E. Vikhrev
*Diogmaglabrata*		MT151828	GBMNB25008-20	BOLD:ABV3921	Finland	63.43	21.074	2019.07.02	L.P. Kolcsár
		MT151829	GBMNB25009-20	BOLD:ABV3921	Finland	60.56	27.838	2016.07.25	E. Viitanen
		MT151792	GBMNB24972-20	BOLD:AED4669	Japan	44.05	145.1	2019.07.26	L.P. Kolcsár
		MT151793	GBMNB24973-20	BOLD:AED4669	Japan	44.05	145.1	2019.07.26	L.P. Kolcsár
		MT151808	GBMNB24988-20	BOLD:AED4670	Japan	35.86	137.51	2016.07.22	D. Kato
		MT151809	GBMNB24989-20	BOLD:AED4670	Japan	35.86	137.51	2016.07.22	D. Kato
		MT151810	GBMNB24990-20	BOLD:AED4670	Japan	39.94	140.86	2015.08.05	D. Kato
		MT151825	GBMNB25005-20	BOLD:ABV3921	Russia	55.36	36.74	2014.06.29	D. Kato
*Liogmabrevipecten*		MT151794	GBMNB24974-20	BOLD:AED7661	Japan	33.56	132.93	2019.06.17	L.P. Kolcsár
		MT151795	GBMNB24975-20	BOLD:AED3259	Japan	33.75	133.15	2019.06.05	L.P. Kolcsár
		MT151803	GBMNB24983-20	BOLD:AED3259	Japan	33.71	133.1	2019.05.18	L.P. Kolcsár
		MT151811	GBMNB24991-20	BOLD:AED7662	Japan	39.94	140.86	2014.07.15	D. Kato
		MT151812	GBMNB24992-20	BOLD:AED8471	Japan	34.59	132.14	2015.05.18	D. Kato
		MT151813	GBMNB24993-20	BOLD:AED7662	Japan	42.92	141.17	2014.06.23	D. Kato
*Liogmamikado*		MT151796	GBMNB24976-20	BOLD:AED6113	Japan	33.48	130.93	2019.05.21	L.P. Kolcsár
		MT151797	GBMNB24977-20	BOLD:AED6113	Japan	33.76	133.12	2019.06.05	L.P. Kolcsár
		MT151814	GBMNB24994-20	BOLD:AED6113	Japan	33.49	130.96	2016.04.22	D. Kato
		MT151815	GBMNB24995-20	BOLD:AED6114	Japan	40.68	140.1	2014.05.11	D. Kato
		MT151816	GBMNB24996-20	BOLD:AED6114	Japan	40.53	140.48	2013.05.31	D. Kato
*Liogmanodicornis*		MT151832	GBMNB25012-20	BOLD:AAK8889	Canada	45.2	-75.83	2011.06.07	F. Brodo
*Liogmaserraticornis*		MT151798	GBMNB24978-20	BOLD:AED5489	Japan	33.48	130.93	2019.05.21	L.P. Kolcsár
		MT151799	GBMNB24979-20	BOLD:AED5489	Japan	33.75	133.15	2019.06.16	L.P. Kolcsár
		MT151817	GBMNB24997-20	BOLD:AED5489	Japan	33.43	130.23	2015.04.23	D. Kato
*Liogmaserraticornis*		MT151818	GBMNB24998-20	BOLD:AED5489	Japan	40.51	140.43	2013.06.08	D. Kato
		MT151819	GBMNB24999-20	BOLD:AED5489	Japan	35.73	138.83	2014.07.08	D. Kato
		MT151820	GBMNB25000-20	BOLD:AED5489	Japan	35.23	137.15	2016.05.04	D. Kato
		MT151824	GBMNB25004-20	BOLD:AED5489	Russia	43.1	131.54	2007.06.13	N.M. Paramonov
*Phalacrocerareplicata*		MT151833	GBMNB25013-20	BOLD:AAD9776	Canada	45.2	-75.83	2017.05.10	F. Brodo
*Phalacroceratipulina*		MT151831	GBMNB25011-20	BOLD:AED8285	USA	37.36	-80.53	2018.02.25	Y. Imada
*Triogmakuwanaikuwanai*	*Triogmakuwanai*	MT151787	GBMNB24967-20	BOLD:AED6747	Japan	40.52	140.34	2013.05.24	D. Kato
		MT151802	GBMNB24982-20	BOLD:AEE0240	Japan	33.75	133.15	2019.06.05	L.P. Kolcsár
		MT151821	GBMNB25001-20	BOLD:AEE0245	Japan	35.35	133.58	2015.05.17	D. Kato
		MT151822	GBMNB25002-20	BOLD:AEE0240	Japan	33.43	130.36	2015.05.02	D. Kato
		MT151823	GBMNB25003-20	BOLD:AED6747	Japan	40.94	140.46	2014.05.15	D. Kato
*Triogmakuwanailimbinervis*	*Triogmakuwanai*	MT151800	GBMNB24980-20	BOLD:AED7834	Japan	33.86	132.77	2019.03.31	L.P. Kolcsár
		MT151801	GBMNB24981-20	BOLD:AED7834	Japan	33.86	132.77	2019.03.31	L.P. Kolcsár
		MT151804	GBMNB24984-20	BOLD:AED7834	Japan	33.86	132.76	2019.04.06	L.P. Kolcsár
**Sequences from BOLDSystems**									
*Cylindrotomaborealis*	*Cylindrotomadistinctissima*		FINTI044-11	BOLD:AAD0770	Finland	60.492	22.302	2011.08.10	J. Salmela
			FINTI045-11	BOLD:AAD0770	Finland	60.223	22.905	2009.08.01	J. Penttinen
			FINTI046-11	BOLD:AAD0770	Finland	62.076	22.492	2010.07.27	J. Salmela, T. Tuovinen
			FINTI047-11	BOLD:AAD0770	Finland	61.066	22.272	2010.08.18	L. Paasivirta
			FINTI054-11	BOLD:AAD0770	Finland	61.34	23.25	2006.08.11	E. Saarela
			FINTI491-12	BOLD:AAD0770	Finland	61.871	24.188	2005.07.30	J. Salmela, J. Kirjavainen
			FINTI507-12	BOLD:AAD0770	Finland	66.373	29.319	2001.08.09	Oulanka Biological Station
			FINTI588-12	BOLD:AAD0770	Finland	60.56	24.218	2011.08.05	E. Viitanen
Cylindrotomacf.distinctissima	*Cylindrotomadistinctissima*		SATIP608-09	BOLD:AAD0770	Germany	47.832	11.793	2009.05.21	C. Young
			SATIP609-09	BOLD:AAD0770	Germany	47.832	11.793	2009.05.21	C. Young
			SATIP610-09	BOLD:AAD0770	Germany	47.832	11.793	2009.05.21	C. Young
			SATIP611-09	BOLD:AAD0770	Germany	47.832	11.793	2009.05.21	C. Young
			SATIP612-09	BOLD:AAD0770	Germany	47.832	11.793	2009.05.21	C. Young
			SATIP613-09	BOLD:AAD0770	Germany	47.832	11.793	2009.05.21	C. Young
			SATIP614-09	BOLD:AAD0770	Germany	48.115	11.206	2009.05.20	C. Young
			SATIP619-09	BOLD:AAD0770	Germany	48.115	11.206	2009.05.20	C. Young
			SATIP1838-12	BOLD:AAD0770	Poland	49.444	21.685	1988.08.25	C. Young
			SATIP1839-12	BOLD:AAD0770	Poland	54.389	18.752	1988.09.04	C. Young
			SATIP1840-12	BOLD:AAD0770	Poland	54.389	18.752	1988.09.04	C. Young
			SATIP1841-12	BOLD:AAD0770	Poland	54.389	18.752	1988.09.04	C. Young
*Cylindrotomad.americana*	*Cylindrotomaamericana*		BBTIP172-10	BOLD:AAV1805	Canada	49.074	-125.8	2010.07.08	BIObus 2010
			BBTIP183-10	BOLD:AAV1805	Canada	49.042	-125.7	2010.07.05	BIObus 2010
			BBTIP220-10	BOLD:AAV1805	Canada	51.265	-117.5	2010.07.16	BIObus 2010
			BBTIP221-10	BOLD:AAV1805	Canada	51.265	-117.5	2010.07.16	BIObus 2010
			CNCDI077-11	BOLD:ABA1601	Canada	52.617	-117.9	2003.07.22	F. Brodo
			CNTMC2308-14	BOLD:AAV1805	Canada	58.451	-62.8	2013.08.16	D. Whitaker
			POSPA900-15	BOLD:AAV1805	Canada	49.301	-123.1	2014.05.26	B. Titaro
			RBNII437-13	BOLD:ABA1601	Canada	53.124	-117.8	2012.06.14	BIOBus 2012
			SSJAA1387-13	BOLD:ABA1601	Canada	53.124	-117.8	2012.06.14	BIOBus 2012
			SSJAA1478-13	BOLD:ABA1601	Canada	53.124	-117.8	2012.06.14	BIOBus 2012
			SSJAA1499-13	BOLD:ABA1601	Canada	53.124	-117.8	2012.06.14	BIOBus 2012
			SSJAA904-13	BOLD:ABA1601	Canada	53.124	-117.8	2012.06.14	BIOBus 2012
			SSJAD5274-13	BOLD:ABA1601	Canada	53.124	-117.8	2012.07.21	BIOBus 2012
			SSJAD6461-13	BOLD:ABA1601	Canada	53.124	-117.8	2012.07.21	BIOBus 2012
			SSJAD6463-13	BOLD:ABA1601	Canada	53.124	-117.8	2012.07.21	BIOBus 2012
			SSJAD6464-13	BOLD:ABA1601	Canada	53.124	-117.8	2012.07.21	BIOBus 2012
			SSJAD6465-13	BOLD:ABA1601	Canada	53.124	-117.8	2012.07.21	BIOBus 2012
			SSJAD6466-13	BOLD:ABA1601	Canada	53.124	-117.8	2012.07.21	BIOBus 2012
			SSJAD6467-13	BOLD:ABA1601	Canada	53.124	-117.8	2012.07.21	BIOBus 2012
			SSJAD6468-13	BOLD:ABA1601	Canada	53.124	-117.8	2012.07.21	BIOBus 2012
			SSJAD6469-13	BOLD:ABA1601	Canada	53.124	-117.8	2012.07.21	BIOBus 2012
			SSJAD6471-13	BOLD:ABA1601	Canada	53.124	-117.8	2012.07.17	BIOBus 2012
			FINTI050-11	BOLD:AAV1805	USA	58.31	-134.4	1988.06.07	F. Brodo
*Cylindrotomad.distinctissima*	*Cylindrotomadistinctissima*		FINTI053-11	BOLD:AAD0770	Czech Rep.	50.03	17.51	2011.05.24	J. Stary
			FINTI042-11	BOLD:AAD0770	Finland	69.035	20.839	2006.07.01	J. Jakovlev, J. Penttinen
			FINTI043-11	BOLD:AAD0770	Finland	69.035	20.839	2006.07.01	J. Jakovlev, J. Penttinen
			FINTI061-11	BOLD:AAD0770	Finland	67.83	26.052	2009.06.30	J. Salmela
			FINTI062-11	BOLD:AAD0770	Finland	67.588	24.214	2006.07.01	J. Penttinen, J. Jakovlev
			FINTI078-11	BOLD:AAD0770	Finland	68.636	22.784	2009.07.22	J. Salmela
			FINTI517-12	BOLD:AAD0770	Finland	63.924	26.869	2008.07.13	J. Salmela
			FINTI743-12	BOLD:AAD0770	Finland	61.926	22.436	2008.07.02	J. Salmela
			FINTI040-11	BOLD:AAD0770	Lithuania	54.115	24.28	2011.08.06	S. Podenas
			FINTI041-11	BOLD:AAD0770	Lithuania	54.115	24.28	2011.08.06	S. Podenas
			FINTI563-12	BOLD:AAD0770	Russia	49.127	154.48	2000.07.28	A.S. Lelej S.Y. Storozhenko
*Cylindrotomad.distinctissima*			FINTI565-12	BOLD:AAD0770	Russia	43.624	132.22	2001.08.26	V.S. Sidorenko
			FINTI566-12	BOLD:AAD0770	Russia	51.791	87.228	2006.07.15	N.M. Paramonov
			FINTI567-12	BOLD:AAD0770	Russia	42.937	133.93	2007.07.16	N.M. Paramonov
			FINTI568-12	BOLD:AAD0770	Russia	55.874	48.723	2010.06.10	N.M. Paramonov
			FINTI569-12	BOLD:AAD0770	Russia	44.154	40.041	2004.06.13	N.M. Paramonov
	*Cylindrotomadistinctissima*		FINTI570-12	BOLD:AAD0770	Russia	44.19	40.066	2007.08.06	N.M. Paramonov
			FINTI571-12	BOLD:AAD0770	Russia	55.911	48.729	2009.06.15	N.M. Paramonov
			FINTI572-12	BOLD:AAD0770	Russia	55.911	48.729	2009.06.15	N.M. Paramonov
			FINTI573-12	BOLD:AAD0770	Russia	60.233	29.163	2007.07.24	N.M. Paramonov
			CNCDI078-11	BOLD:AAD0770	Sweden	60.05	17.333	1992.06.10	F. Brodo
			FINTI1082-12	BOLD:AAD0770	Sweden	68.334	18.794	2002.07.17	J. Kramer
*Cylindrotomadistinctissima*	*Cylindrotomaamericana*		SSBAB2554-12	BOLD:AAV1805	Canada	51.35	-116.1	2012.06.19	BIOBus 2012
			SSBAB3039-13	BOLD:AAV1805	Canada	51.35	-116.1	2012.06.20	BIOBus 2012
			SSBAE1284-13	BOLD:AAV1805	Canada	51.35	-116.1	2012.07.28	BIOBus 2012
			SSBAE1285-13	BOLD:AAV1805	Canada	51.35	-116.1	2012.07.28	BIOBus 2012
			SSBAE1289-13	BOLD:AAV1805	Canada	51.35	-116.1	2012.07.28	BIOBus 2012
			SSBAE1292-13	BOLD:AAV1805	Canada	51.35	-116.1	2012.07.28	BIOBus 2012
			SSBAE1293-13	BOLD:AAV1805	Canada	51.35	-116.1	2012.07.28	BIOBus 2012
			SSBAE1294-13	BOLD:AAV1805	Canada	51.35	-116.1	2012.07.23	BIOBus 2012
			SSBAE1295-13	BOLD:AAV1805	Canada	51.35	-116.1	2012.07.23	BIOBus 2012
			SSGBB7185-14	BOLD:AAV1805	Canada	49.429	-57.74	2013.07.20	BIObus 2013
*Cylindrotomanigriventris*			FINTI048-11	BOLD:ABV9491	Finland	61.109	24.264	2009.06.24	J. Penttinen
			FINTI049-11	BOLD:ABV9491	Finland	60.427	24.922	2009.06.24	J. Penttinen
			FINTI745-12	BOLD:ABV9491	Finland	62.075	22.492	2010.06.17	J. Salmela, T. Tuovinen
*Diogmacaudata*			FINTI080-11	BOLD:ABV3921	Finland	66.335	29.513	2005.08.03	J. Salmela
			FINTI087-11	BOLD:ABV3921	Finland	66.335	29.513	2005.08.03	J. Salmela
Diogmacf.glabrata			FINTI1135-12	BOLD:ABV3921	Finland	60.491	22.302	2011.05.23	J. Salmela
			FINTI1136-12	BOLD:ABV3921	Finland	60.491	22.302	2011.05.23	J. Salmela
			AMTPD3808-15	BOLD:ABV3921	Germany	47.387	10.344	2014.07.20	D. Doczkal
*Diogmaglabrata*			FINTI1025-12	BOLD:ABV3921	Finland	61.837	24.064	2006.08.03	J. Salmela
			FINTI235-12	BOLD:ABV3921	Finland	63.407	28.2	2008.07.14	J. Salmela
			FINTI842-12	BOLD:ABV3921	Finland	63.941	26.663	2008.07.13	J. Salmela
			FINTI918-12	BOLD:ABV3921	Finland	62.201	22.454	2008.08.08	J. Salmela
Liogmacf.nodicornis			SATIP1842-12	BOLD:AAK8889	USA	40.422	-80.17	1998.05.20	D. Koenig
			SATIP1845-12	BOLD:AAK8889	USA	41.558	-80.2	1998.05.18	C. Young, D. Koenig, T. Tomon
			SATIP268-09	BOLD:AAK8889	USA	40.612	-79.95		C. Young
*Liogmanodicornis*			BBTIP158-10	BOLD:AAK8889	Canada	48.593	-86.29	2010.06.10	BIObus 2010
			BBTIP162-10	BOLD:AAK8889	Canada	48.593	-86.29	2010.06.10	BIObus 2010
			CNCTI002-12	BOLD:AAK8889	Canada	45.4	-75.85	1995.06.09	F. Brodo
			CNCTI003-12	BOLD:AAK8889	Canada	45.4	-75.85	1995.06.09	F. Brodo
			CNCTI006-12	BOLD:AAK8889	Canada	45.267	-75.8	2011.06.07	F. Brodo
			CNCTI007-12	BOLD:AAK8889	Canada	45.267	-75.8	2011.06.07	F. Brodo
			CNFNE3074-14	BOLD:AAK8889	Canada	48.857	-64.38	2013.07.05	F. Tremblay
			CNRGK935-15	BOLD:AAK8889	Canada	43.822	-79.19	2014.06.10	K. Kerr, A. Sritharan
			CNTIC6257-15	BOLD:AAK8889	Canada	44.453	-75.87	2014.06.11	M. Brown
			JSDIQ829-10	BOLD:AAK8889	Canada	44.621	-75.77	2010.05.30	J. Sones
			OPPAM1198-17	BOLD:AAK8889	Canada	45.256	-77.19	2014.06.18	CBG Collections Staff
			SSEIC5992-13	BOLD:AAK8889	Canada	53.663	-112.8	2012.07.01	BIOBus 2012
			SSROC9031-15	BOLD:AAK8889	Canada	43.811	-79.16	2013.06.09	BIObus 2013
			GMFRQ424-15	BOLD:AAK8889	USA	38.892	-78.17	2014.06.02	K.J. Anderson
*Phalacrocerareplicata*			CNTIA2077-15	BOLD:AAD9776	Canada	44.453	-75.87	2014.05.14	M.B. Lynch
			CNTIA2078-15	BOLD:AAD9776	Canada	44.453	-75.87	2014.05.14	M.B. Lynch
			CNTIA2079-15	BOLD:AAD9776	Canada	44.453	-75.87	2014.05.14	M.B. Lynch
			CNTIA2081-15	BOLD:AAD9776	Canada	44.453	-75.87	2014.05.14	M.B. Lynch
			CNTIB1805-15	BOLD:AAD9776	Canada	44.453	-75.87	2014.05.14	M. Brown
			OPPOA298-17	BOLD:AAD9776	Canada	44.283	-77.8	2014.05.23	CBG Collections Staff
			PHTCH356-08	BOLD:AAD9776	Canada	58.741	-93.82	2008.07.16	C.W.Young
			PHTCH357-08	BOLD:AAD9776	Canada	58.741	-93.82	2008.07.16	C.W.Young
			PHTCH358-08	BOLD:AAD9776	Canada	58.741	-93.82	2008.07.16	C.W.Young
			PHTCH359-08	BOLD:AAD9776	Canada	58.741	-93.82	2008.07.16	C.W.Young
			PHTCH385-08	BOLD:AAD9776	Canada	58.741	-93.82	2008.07.16	C.W.Young
			FINTI310-12	BOLD:AAD9776	Finland	69.746	27.822	2007.02.07	J. Salmela
			FINTI805-12	BOLD:AAD9776	Finland	63.433	27.53	2008.06.04	J. Salmela
			CNCDI081-11	BOLD:AAD9776	Norway	60.6	7.5	1992.07.17	F. Brodo
			CNCTI005-12	BOLD:AAD9776	Norway	60.6	7.5	1992.07.17	F. Brodo
*Triogmatrisulcata*			FINTI801-12	BOLD:ABW4579	Finland	63.433	27.53	2008.06.04	J. Salmela
			FINTI928-12	BOLD:ABW4579	Finland	62.215	25.742	2005.06.09	J. Salmela
*Limoniaphragmitidis*			FINTI876-12	BOLD:ABV3744	Finland	62.22	25.77	2006.08.10	J. Salmela
*Pediciarivosa*			FINTI657-12	BOLD:ABW1968	Finland	69.751	27.88	2006.07.03	J. Salmela
*Tipulamaxima*			FINTI636-12	BOLD:AAD6106	Finland	60.333	24.501	2007.07.19	J. Ilmonen

## ﻿Results

### ﻿Molecular analyses

**Figure 1.**
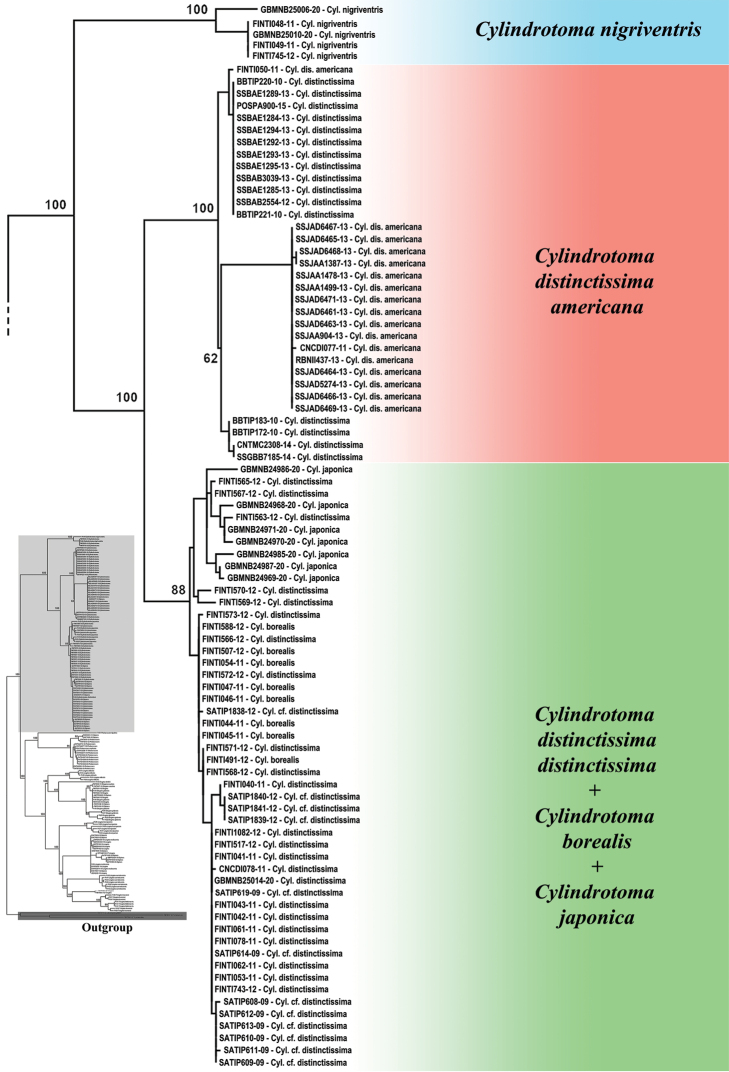
Partial maximum likelihood tree based on COI marker showing the clade of *Cylindrotoma* sequences, which is magnified from the entire tree on the left as highlighted with pale grey. Outgroup highlighted with dark grey. Numbers at nodes indicate bootstrap values of major clades. Sequence identifiers are BoldSystems numbers, see Table 1 for further information.

**Figure 2.**
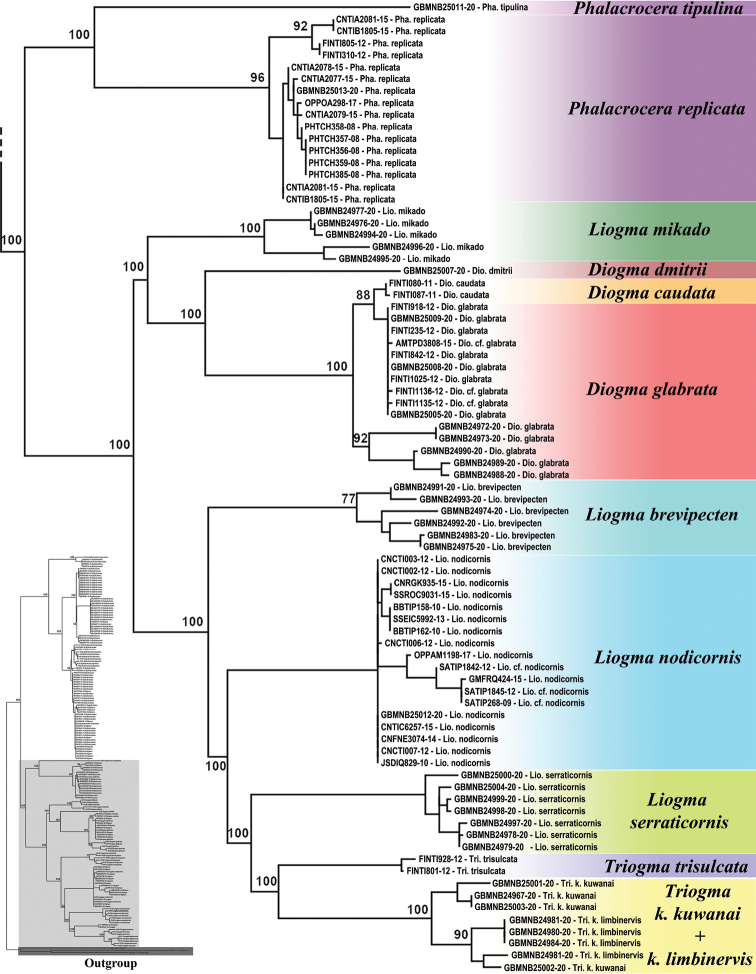
Partial maximum likelihood tree based on COI marker showing the clades of *Phalacrocera*, *Diogma*, *Liogma*, and *Triogma* sequences, which is magnified from the entire tree on the left as highlighted with pale grey. Outgroup highlighted with dark grey. Numbers at nodes indicate bootstrap values of major clades. Sequence identifiers are BoldSystems numbers, see Table 1 for further information.

A maximum likelihood tree based on the COI barcode sequences is shown in Figures 1 and 2. To make viewing easier the tree was divided into two parts, with Figure 1 showing the *Cylindrotoma* clade, and Figure 2 consisting of *Diogma*, *Liogma*, *Triogma*, and *Phalacrocera* representing the sister clade.

The tree (Fig. 1) illustrates *Cylindrotomanigriventris* Loew, 1849 as the sister group of *C.distintissima* which consists of two subspecies, *Cylindrotomad.americana* Osten Sacken, 1865 in the Nearctic and *Cylindrotomad.distinctissima* (Meigen, 1818) in the Palearctic. Validity of *C.japonica* Alexander, 1919 was not supported because *Cylindrotomad.distinctissima* (Meigen, 1818) formed a clade together with *C.japonica* Alexander, 1919.

The monophyly of *Phalacrocera* was recovered based on the sequences of two species, *P.replicata* (Linnaeus, 1758) and *P.tipulina* Osten Sacken, 1865. The sequences from the Nearctic and Western Palearctic specimens of *P.replicata* formed the respective clades.

Within Figure 2*Liogmamikado* (Alexander, 1919) is placed as sister to *Diogma*. In the case of *Diogma*, *D.dmitrii* Paramonov, 2005 represented the sister species of *D.caudata* Takahashi, 1960 and *D.glabrata* (Meigen, 1818). The sequences from the latter species were not separated, and the two sequences of *D.caudata* from Finnish specimens were closely related to the clade of sequences of *D.glabrata* of Finnish specimens, while the sequences of *D.glabrata* from Japanese specimens formed a separate clade.

Although two species of *Triogma* were monophyletic, the clade was nested in the clade of *Liogma* spp., with exception of the aforementioned *L.mikado*. Four species, *Liogmabrevipecten* Alexander, 1932, *L.nodicornis* (Osten Sacken, 1865), *L.serraticornis* Alexander, 1919, and *Triogmatrisulcata* (Schummel, 1829), represented a distinct clade. Two subspecies of *Triogmakuwanai* (Alexander, 1913), *T.k.kuwanai* and *T.k.limbinervis*, were not clearly distinguished.

### ﻿Taxonomic treatment

Based upon our morphological comparison and genetic analyses, the two subspecies of *Cylindrotomadistinctissima*, the Palearctic *C.d.distinctissima* and the Nearctic *C.d.americana* represent separate lineages. Therefore, we propose the elevation of these subspecies to species rank as *C.americana* stat. reval. and *Cylindrotomadistinctissima*. Furthermore, *Cylindrotomajaponica* syn. nov. and *C.distinctissimaalpestris* Peus, 1952 syn. nov. are treated as junior synonyms of *C.distinctissima*. Similarly, *Triogmakuwanailimbinervis* syn. nov. and *T.nimbipennis* Alexander, 1941 syn. nov. are junior synonyms of *Triogmakuwanai*. Each case is discussed in detail under the corresponding species discussion.

Cylindromine species that occur in Japan are redescribed, along with their habitus and wing photographs and the illustrations of male and female terminalia. The male and female terminalia of *Cylindrotomanigriventris* Loew, 1849, *Diogmadmitrii* Paramonov, 2005, *Liogmanodicornis* (Osten Sacken, 1865), *Phalacrocerareplicata* (Linnaeus, 1758), *P.tipulina* Osten Sacken, 1865, and *Triogmatrisulcata* (Schummel, 1829) are also illustrated and described in detail.

***Cylindrotoma* Macquart, 1834**

#### ﻿ Cylindrotoma distinctissima

Taxon classificationAnimaliaDipteraCylindrotomidae

(Meigen, 1818)

565C6AA8-CC18-5836-B8F3-EABE25232A20

**Figure 3.**
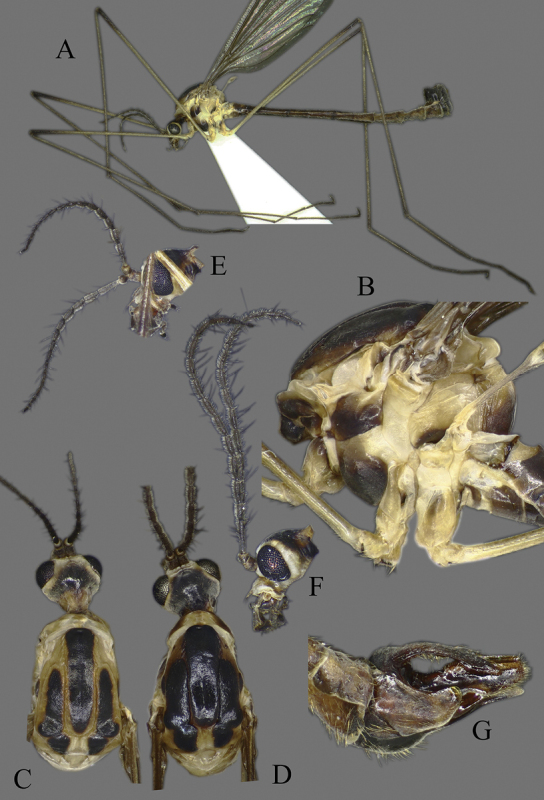
*Cylindrotomadistinctissima* (Meigen, 1818) **A** habitus of male, lateral view (colouration of wings is artefact) **B** thorax of male, lateral view **C** head and thorax dorsal view of pale “*distinctissima*” form **D** head and thorax dorsal view of dark, “*japonica*” form **E** head of female, lateral view **F** head of male, lateral view **G** female terminalia lateral view.

**Figure 4.**
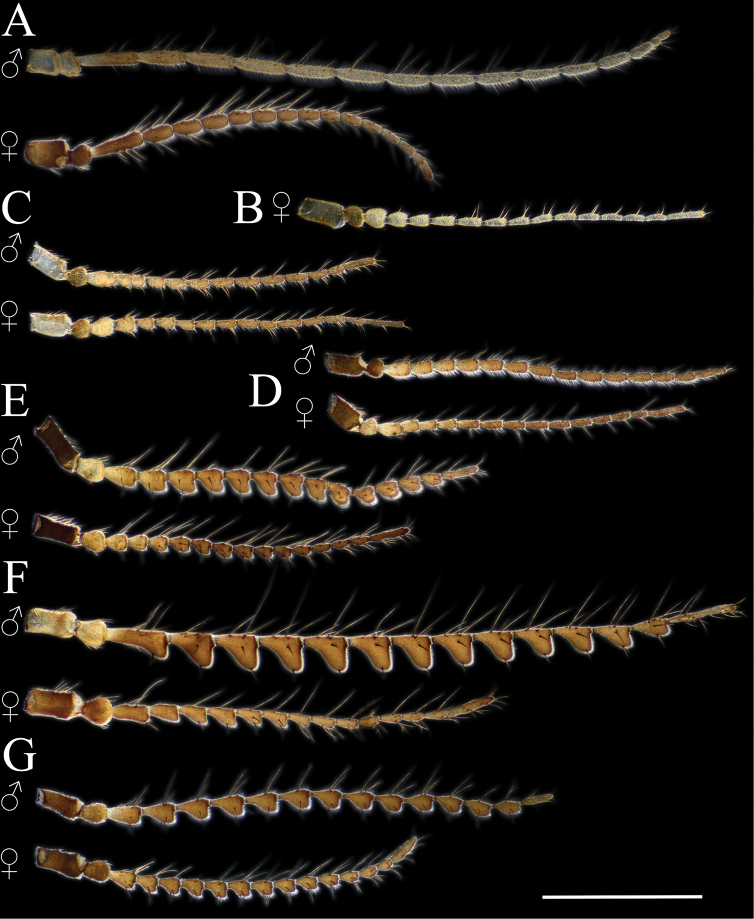
Antennae **A***Cylindrotomadistinctissima* (Meigen, 1818) **B***Diogmacaudata* Takahashi, 1960 **C***Diogmaglabrata* (Meigen, 1818) **D***Liogmamikado* (Alexander, 1919) **E***Liogmabrevipecten* Alexander, 1932 **F***Liogmaserraticornis* Alexander, 1919 **G***Triogmakuwanai* (Alexander, 1913). Scale bar: 1 mm.

**Figure 5.**
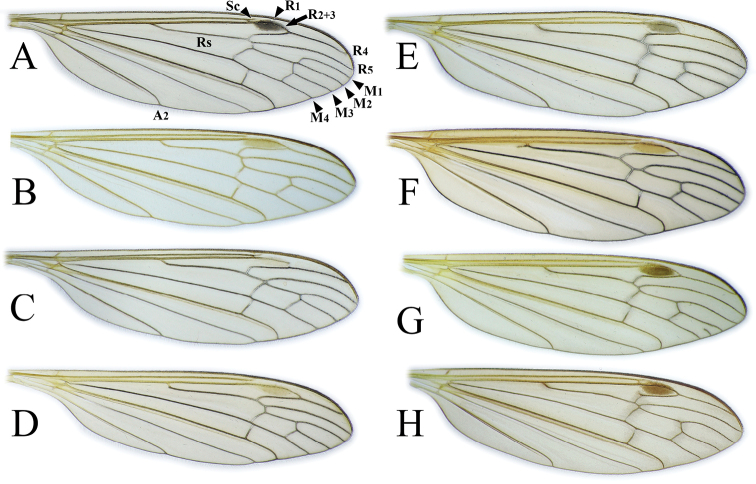
Wing **A***Cylindrotomadistinctissima* (Meigen, 1818) **B***Diogmacaudata* Takahashi, 1960 **C***Diogmaglabrata* (Meigen, 1818) **D***Liogmamikado* (Alexander, 1919) **E***Liogmabrevipecten* Alexander, 1932 **F***Liogmaserraticornis* Alexander, 1919 **G***Triogmakuwanai* (Alexander, 1913) of “*kuwanai*” form **H***Triogmakuwanai* (Alexander, 1913) of “*limbinervis*” form.

**Figure 6.**
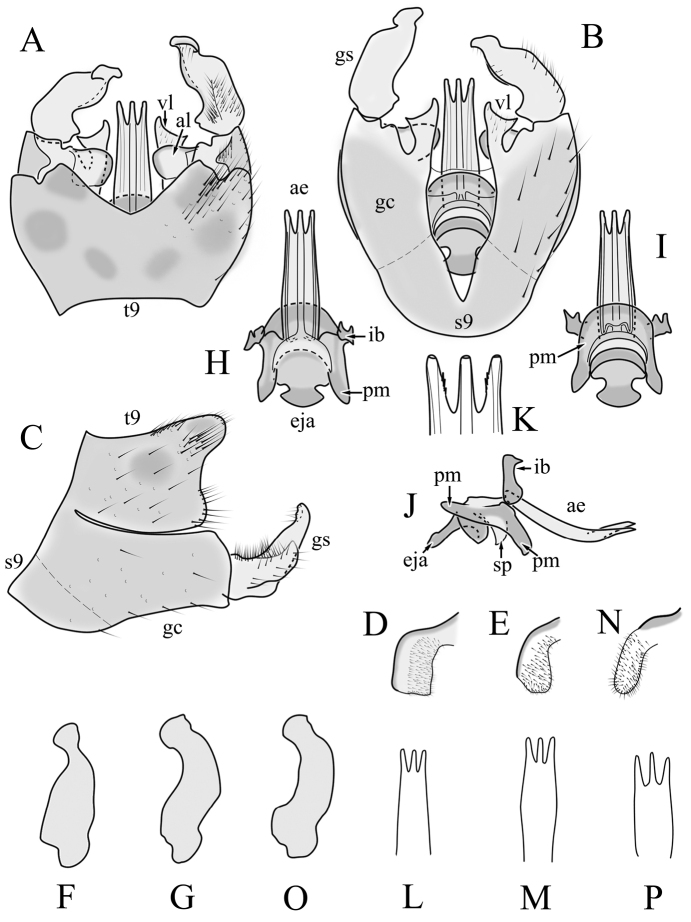
Male genital structures of *Cylindrotomadistinctissima* (Meigen, 1818) (**A–M**), in comparison to *C.americana* Osten Sacken, 1865 (**N–P**) **A** terminalia, dorsal view **B** terminalia, ventral view **C** terminalia, lateral view; **D** Apical lobe of the gonocoxite (Japan) **E** apical lobe of the gonocoxite (Finland) **F** shape of the gonostylus caudal view (Japan) **G** shape of the gonostylus caudal view – Finland **H** aedeagus complex, dorsal view **I** aedeagus complex, ventral view **J** aedeagus complex, lateral view **K** tip of the aedeagus **L** shape of the aedeagus (Japan) **M** shape of the aedeagus (Finland); *C.americana* Osten Sacken **N** apical lobe of the gonocoxite **O** shape of the gonostylus caudal view **P** shape of the aedeagus. Abbreviations: ae – aedeagus; al – gonocoxite apical lobe; eja – ejaculatory apodeme; gc – gonocoxite; gs – gonostylus; ib – interbase; pm – paramere; sp – sperm pump; s9 – sternite 9; t9 – tergite 9; vl – gonocoxite ventral lobe.

**Figure 7.**
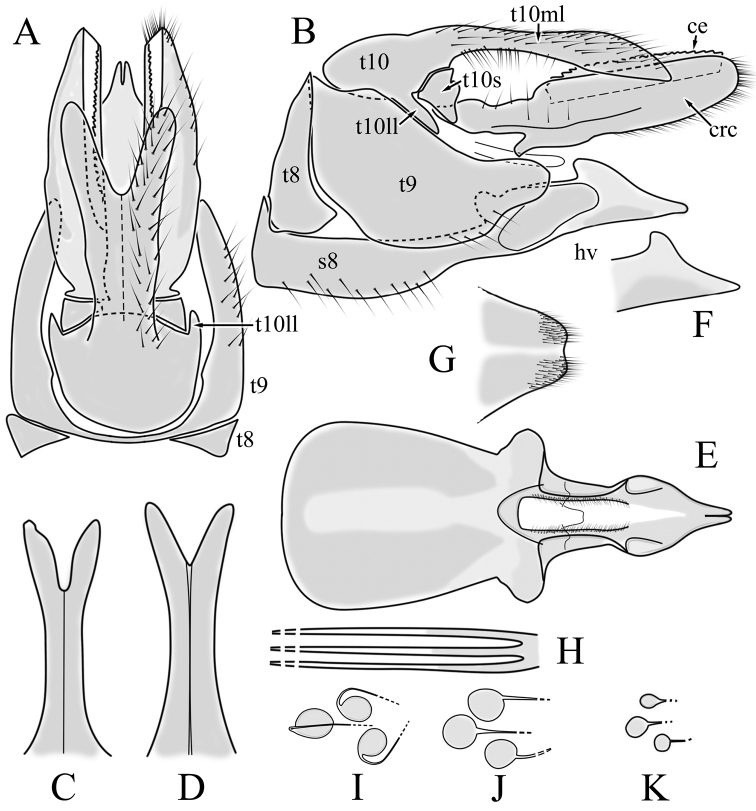
Female genital structures of *Cylindrotomadistinctissima* (Meigen, 1818) (**A–J**) and *C.americana* Osten Sacken, 1865 (**K**) **A** terminalia, dorsal view **B** terminalia, lateral view **C** shape variant of median lobe of tergite 10 (Krasnodar Krai, Russia) **D** shape variant of median lobe of tergite 10 (Finland) **E** sternite 8 and hypogynial valves, inner dorsal view **F** shape variant of tip of the hypogynial valve **G** sternite 10 **H** genital opening and sperm ducts **I** spermathecae (Japan) **J** spermathecae (Finland) **K** spermathecae of *C.americana* Osten Sacken, 1865. Abbreviations: ce – cutting edge; crc – cercus; hv – hypogynial valve; t8 – tergite 8; t9 – tergite 9; t10 – tergite 10; t10s – tergite 10 triangular sclerite; t10ll – tergite 10 lateral lobe; s8 – stergite 8.

**Figure 8.**
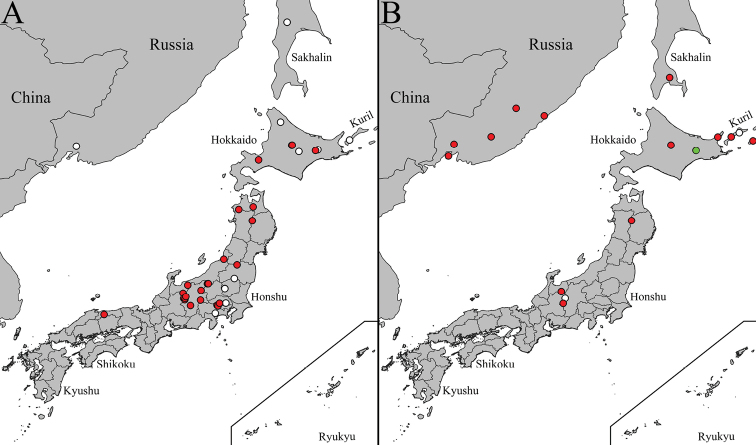
Occurrence data in Japan and surrounding areas of **A***Cylindrotomadistinctissima* (Meigen, 1818) **B***Diogmaglabrata* (Meigen, 1818) and *D.caudata* Takahashi, 1960. Red dots indicate locations of investigated specimens, white dots indicate approximate locations of literature data. Green dot indicate approximate location of type locality of *D.caudata* Takahashi, 1960.

Figs 3 , 4A , 5A , 6 , 7 , 8A

Tipula brevicornis (Zetterstedt, 1838)
Cylindrotoma tenebrarum Krogerus, 1937
Cylindrotoma distinctissima borealis Peus, 1952
Cylindrotoma japonica Alexander, 1919, syn. nov.; Alexander 1919: 344–345: original description; Alexander 1924: 595: faunistic records; Alexander 1928: 9: distribution, illustrations; Esaki 1950: 1513: illustration; Ishida 1955: 77: distribution; Takahashi 1960: 81: distribution; Alexander 1966: 122: distribution, faunistic records; Sidorenko 1999: 68–70: identification key, illustration, distribution; Nakamura 2001: 23–29: identification key, illustration, distribution, faunistic records; Pilipenko and Sidorenko 2004: 12 faunistic records; Boldgiv 2006: phylogeny, faunistic records; in Paramonov 2006 as Cylindrotomadistinctissimajaponica: 888: stat. nov., identification key, illustration, distribution; Gelhaus et al. 2007: 64 comparison; Sasakawa 2008: 131: faunistic records; Nakamura 2014: 54: distribution; Kato and Suzuki 2017: 16: faunistic records, distribution; Imada 2020: biology and ecology of larvae.
Cylindrotoma distinctissima alpestris Peus, 1952, syn. nov.: Peus 1952: original description.

##### Type material examined.

*Cylindrotomajaponica* Alexander, 1919: ***Paratype***. **Japan** • ♀; Saitama Pref., Saitama; 31 May 1919; R. Takahashi leg.; USNM.

##### Non-type material examined.

*Cylindrotomadistinctissimadistinctissima* (Meigen, 1818): **Finland** • 1 ♂; Vieremä, Mammonhauta; 63.924404°N, 26.869023°E; alt. 135; 18 Jun. 2008 – 13 Jul. 2008; J. Salmela leg.; CKLP. **Russia** • 1 ♂, 1 ♀; Krasnodar Krai, Apsheronsky District, Mezmay Settlement, Kamyshanova polyana, Mezmaika River; 44.16989°N, 40.05181°E; alt. 1200 m; 11 Jun. 2004; N.M. Paramonov leg.; CKLP.

*Cylindrotomajaponica* Alexander, 1919: **Japan** • 1 ♂; Mt. Shirouma Alps, 36.78°N, 137.7°E; 8 Aug. 1931; J. Machida leg.; USNM. • 1 ♀; Aomori, Towada, Sakura Spa, Okuse; 40.627315°N, 140.909831°E; alt. 854 m; 21 Jun. 2014, D. Kato leg.; BLKU. • 1 ♂, 1 ♀; Aomori, Nishimeyamura, Okawa Path, Kawaratai; 40.500625°N, 140.204058°E; alt. 300 m; 18 Sep. 2013; D. Kato leg.; BLKU. • 1 ♂, reared from larva; Gifu, Takayama, Nigorikawa; 36.0545°N, 137.55818°E; 1375 m; larva collected: 5 Aug. 2015, emerged: 26 May. 2015; M. Kato leg.; CYI. • 1 ♂; Gifu, Mt. Norikura, Japanese Alps; 36.12°N, 137.5°E; 26 Jun. 1929; J. Machida leg.; USNM. • 1 ♀; Hokkaido, Sapporo, Minami-ku, Jozankei, trail of Mt. Sapporo; 42.92392°N, 141.17688°E; alt. 450–860 m; 3 Sep. 2018; D. Kato leg.; BLKU. • 2 ♂, 3 ♀; Hokkaido, Higashikawa, Asahidake, River Yukomabetsu; 43.65226°N, 142.80229°E; alt. 1120 m; 23 Jul. 2019; L.-P. Kolcsár leg.; CKLP. • 2 ♂; Hokkaido, Higashikawa, Asahidake; 43.65582°N, 142.82608°E; alt. 1100–1500 m; 24 Jul. 2019; L.-P. Kolcsár, leg.; CKLP. • 1 ♂; Hokkaido, Ashoro, Meakan Moutain, small sandy/muddy stream; 43.3907°N, 143.96821°E; alt. 365 m; 27 Jul. 2019; L.-P. Kolcsár leg.; CKLP. • 1 ♀; Iwate, Hachimantai, Toshiti Spa; 39.94253°N, 140.86804°E; alt. 1344 m; 3 Aug. 2013; • 1 ♀; same locality; 1 Jul. 2014; • 1 ♂; same locality; 5 Aug. 2014; • 2 ♀; same locality; 20 Sep. 2014; • 1 ♀; same locality; 5 Aug. 2015; D. Kato leg.; BLKU. • 3 ♂; Nagano, Matsumoto, Azumi, Mt. Norikura, near Kuraigahara-Sansou; 36.11987°N, 137.5692°E; alt. 2370 m; 22 Jul. 2016; D. Kato leg.; BLKU. • 1 ♂; Nagano, Ueda, Daimyozin stream, Sugadaira MRC; 36.51992°N, 138.3539°E; alt. 1315 m; 27 Aug. 2012; D. Kato leg.; BLKU. • 2 ♂; Nagano, Sakae-mura, Sakai, Koakazawa-gawa River; 36.85352°N, 138.66358°E; alt. 1320–1600 m; 19. Sep. 2019; D. Kato leg.; BLKU. • 1 ♂; Nagano, Chino, Shibunoyu; 36.03582°N, 138.32771°E; alt. 1863 m; 21 Jul. 2013; M. Kato leg.; CYI. • 2 ♂; Nagano, Miyada, Kisokomagatake; 35.76917°N, 137.8357°E; alt. 1683 m; 13 Aug. 2013; M. Kato leg.; CYI. • 1 ♂; Nagano, Matsumoto, Kamikouchi; 36.20966°N, 137.60662°E; alt. 1320 m; 3 Aug. 2014; M. Kato leg.; CYI. • 1 ♀; Niigata, Yuzawa, Mitsumata, Mt. Naeba; 36.85616°N, 138.71041°E; alt. 1500–1900 m; 8 Aug. 2019; D. Kato leg.; BLKU. • 1 ♂; Niigata, Kurokawa, Echigo; 38.05°N, 139.47°E; 19 May 1954; B. Kintaro leg.; USNM. • 1 ♀; Okayama, Maniwa, Hiruzen-Shimotokuyama; 35.32931°N, 133.59725°E; alt. 784 m; 17 May. 2015; D. Kato leg.; BLKU. • 2 ♀; Tokyo, Tokyo, Akiruno, rocky river and stream; 35.74766°N, 139.18466°E; alt. 288 m; 11 May. 2019; L.-P. Kolcsár leg.; CKLP. • 1 ♂; Tokyo, Tokyo, Mitake; 35.78°N, 139.14°E; 10 May. 1931; B. Oda leg.; USNM. • 1 ♂; Toyama, Kurobegoro; 36.38°N, 137.47°E; 8 Aug. 1931; Imanishi leg.; USNM. • 1 ♀; Yamagata, Yonezawa, Shirabu-onsen; 37.77646°N, 140.11964°E; alt. 888 m; 26 Jun. 2015; Y. Imada leg.; CYI. **Russia** • 1 ♂; Saghalien [Far East, Sakhalin Oblast], Shimizu; 1922.07.27, T. Esaki leg.; USNM.

##### Redescription.

Colouration very variable, base colour whitish yellow to dark orange, with pale brown to black markings.

**Head**. Vertex and occiput with dark area, size variable among specimens, larger on “*borealis*” and “*japonica*” form; yellowish around eye (Fig. 3C, D, F). Rostrum short, yellow to brown, without nasus, but with tuft of hairs (Fig. 3F, E). Palpus five segmented, last segment 2 × longer than penultimate segment. Antenna yellowish brown to black (Fig. 3F, E); scape short, as long as wide; pedicel short, subspherical to drop-shaped; flagellum 14 segmented (Fig. 4A). Flagellar segments simple in both sexes, not expanded ventrally, covered with dense, whitish setae (sensilla), especially in ventral side (Figs 3E, F, 4A); sensilla less dense in female; first flagellomere longer than second in both sexes; verticels black, relatively long.

**Thorax**. Whitish yellow to dark orange, with contrasting black marks. Cervical sclerites brown to black. Pronotum pale in middle, darker laterally (Fig. 3B, C, D). Mesonotal pattern variable, from three longitudinal, pale brown (“*alpestris*” form) to black (the typical “*distinctissima*” form Fig. 3C) markings to one large patch (“*japonica*” form Fig. 3D); longitudinal mesonotal suture distinct, formed by deep groove (Fig. 3C, D). Scutellum yellow, triangular (Fig. 3C, D). Mediotergite yellow, posterior part black (Fig. 3B). Anepisternum and katepisternum separated, both darker ventrally (Fig. 3B). Katatergite yellow, black above posterior spiracle, with creases. Coxa base yellow to pale brown, apically yellow, trochanter yellowish (Fig. 3B); femur and tibia yellowish, with distinct and wide, black ring at tip; tarsus uniformly black. Stem of halter yellow, knob usually darker. Wing hyaline, with yellowish brown to brown tinge; veins brown to black; pterostigma brown to black (Fig. 5A); wing membrane with interference patterns, visible with dark background (Fig. 3A). Four branches of M reaching wing margin. Cell a2 less than 6 × longer than wide.

**Abdomen**. Yellow (“*alpestris*” form) to almost black (“*japonica*” form); gradually lightening caudally, without clear pattern or with narrow longitudinal line medially.

**Male terminalia**. Black, directed dorsally (Fig. 3A). Tergite 9 partly fused with gonocoxite (Fig. 6C). Caudal margin of tergite 9 with deep V-shaped notch at middle (Fig. 6A); posterior edge of tergite 9 forming dorsal and ventral portion in lateral view (Fig. 6C), shapes variable among specimens. Gonocoxite fused with sternite 9 (Fig. 6B, C); gonocoxite with ventral crescent-shaped lobe (Fig. 6A, B: vl); apical lobe of gonocoxite (al) prominent, well separated, directed inward; both ventral and inner lateral margins sclerotised, shape variable (Fig. 6A, D, E). Gonostylus undivided; twisted, widening in caudal view, shape variable among and within population(s) (Fig. 6F, Japan; Fig. 6G, Finland). Interbase small, without membranous or sclerotised lobe between interbases (Fig. 6H, I). Aedeagus dorsoventrally flattened, gently curved dorsally (Fig. 6J), gradually narrowing to tip, shape variable among and within population(s) (Fig. 6H, I, L, Japan; Fig. 6M, Finland); tip divided into three short, nearly equal tubes in last 1/4 of its length (Fig. 6L, M). Spines on lateral branch of aedeagus small, indistinct (Fig. 6K).

**Female terminalia**. Brown to black, strongly sclerotised (Fig. 3G). Tergite 8 separated in middle by membranous area (Fig. 7A). Tergite 9 larger than tergite 8 in lateral view (Fig. 7B). Tergite 10 with elongated Y-shaped projection, shape variable among specimens (Fig. 7A, Japan; Fig. 7C, Russia (Krasnodar Krai), Fig. 7D, Finland). Cercus with serrate, cutting edge on inner-dorsal surface (Fig. 7A, B). Hypogynial valve on dorsal side with bulbous or triangular projection near middle, shape variable within specimens (Fig. 7B, F); distal part of hypogynial valve narrowing to tip. Three, relatively large spermathecae present, diameter ~ 0.15–0.2 of wide of sternite 8; duct of spermatheca straight or curved (Fig. 7I, J). Sperm ducts simple, without darkened areas (Fig. 7H). Sternite 10 with a small notch at tip, less sclerotised at midline (Fig. 7G).

##### Distribution.

Widely distributed species in Palearctic, known from: Austria, Belarus, Belgium, Bulgaria, Croatia, Czech Rep., Denmark, Estonia, Finland, France, Germany, Great Britain, Hungary, Ireland, Italy, Kazakhstan, Lithuania, Luxembourg, Mongolia, Netherlands, Norway, Poland, Romania, Russia (North European territory, Central European territory, South European territory, West Siberia (Altay), Far East (Kamchatka Krai, Primorsky Krai, Sakhalin Oblast (incl. Kuril I), Serbia, Slovakia, Slovenia, Spain, Sweden, Switzerland, Ukraine, and Turkey (Paramonov and Lobkova 2013; Devyatkov 2021; Oosterbroek 2021). Distribution records of *C.japonica* transferred to *C.distinctissima*: Mongolia and Japan (Hokkaido I, Honshu I, and Kyushu I) (Fig. 8A).

##### Comments.

The species was originally described 250 years ago from Europe, where it is among the most widespread of cylindrotomines. The colour polymorphisms of *C.distinctissima* have been described as separate species or subspecies. Peus (1952) separated three subspecies, the nominate subspecies *C.d.distinctissima* (Meigen, 1818), widespread in Europe, *C.d.borealis* Peus, 1952 from Norway, and *C.d.alpestris* Peus, 1952 from Italian Alps. Later, *Cylindrotomad.borealis* was raised to species rank based on the generally darker habitus and slightly different genital characters (Salmela and Autio 2007). As the COI gene sequence’s genetic distance between *C.d.distinctissima* and *C.borealis* was low, the species was later synonymised with *C.d.distinctissima* (Salmela 2013). In our ML tree, *C.borealis* sequences were also not separated from *C.d.distinctissima* sequences. *Cylindrotomad.alpestris* was treated as species in CCW (2018–2021), because it showed the sympatric distribution with *C.d.distinctissima* in Alps (Italy). This subspecies was designated based on very pale colouration, compared with the nominative subspecies, but the male terminalia does not show any differentiation, which was highlighted in the original description by Peus (1952). Peus noted that this subspecies maybe just a local colour variation, as *Cylindrotoma* specimens showed colour polymorphisms, especially in mountain specimens (as noted by the personal experience of N. Paramonov), but there is no genital differentiation between the two species, and therefore we synonymise *C.d.alpestris* syn. nov. with *C.distinctissima*.

Another species related to *C.distinctissima* was described from Japan. The description of *Cylindrotomajaponica* Alexander, 1919, was based on the darker colouration of the thorax (Fig. 3D) (Alexander 1919). The rank of this species was first questioned by Paramonov (2006), who referred to it as a subspecies of *C.distinctissima* in his identification key of The Cylindrotomidae of Far East Russia. Our morphological and genetic comparisons suggest that *C.japonica* does not differ significantly from *C.distinctissima*, even at the subspecies level. The colouration of *C.japonica* shows a high level of variability in Japan. The specimens collected in Hokkaido Island, have, typically, three separated black marks on the mesonotum (Fig. 3C). Small genital differences occur between the typical examples of *C.distinctissima* and *C.japonica*, in the shape of the apical gonocoxal lobe (rectangular in Japanese specimens (Fig. 6D) and less sclerotised and rounded in studied European specimens Fig. 6E), the shape of aedeagus (evenly narrowing in Japanese specimens Figure 6L, and broader at the middle in examined European specimens Fig. 6M), as well as the shape of the gonostylus in caudal view (Fig. 6F, G). However, these also show variability amongst specimens (see illustrations by Peus 1952: fig. 27; Salmela and Autio 2007; figs 1e, 2b, e). Ujvárosi et al. (2011: fig. 2) illustrated the high variability level of the ventral lobe of the gonocoxite in Bulgarian and Romanian populations in the case of *C.d.distinctissima*, but we did not find that similar variability in *C.japonica* specimens examined. *C.japonica* syn. nov. and *C.distinctissima* are now synonymised based on the high colour variability level, the minimal genital differences, and the small genetic differentiation between the species.

Four species of *Cylindrotoma* have been described from the Nearctic, which are related to *C.distinctissima*, namely *C.americana* Osten Sacken, 1865, *C.juncta* Coquillett, 1900, *C.splendens* Doane, 1900, and *C.pallescens* Alexander, 1931. After the revision of North American Cylindrotomidae, these later three species were synonymised with *C.americana*, and the latter species was treated as a subspecies of *C.distinctissima* as *C.distinctissimaamericana* Osten Sacken, 1865, as their male terminalia were highly similar to each other (Brodo 1967). Molecular analysis shows a relatively high (~ 4.6%) genetic distance between the Nearctic and Palearctic subspecies, and a slight genital difference between these two clades was found in our study (see below the comparative diagnosis of *C.americana*). Based upon the two subspecies’ genetic and geographic separation, the two subspecies are now raised to species rank, *C.americana* stat. reval. and *C.distinctissima*. Furthermore, the Nearctic *C.americana* shows an additional molecular differentiation, as specimens from Jasper National Park, Alberta, Canada were found to belong to a separate barcode BIN (BOLD:ABA1601), and the remaining sequences, both from western and eastern parts of North America represent another barcode BIN (BOLD:AAV1805). The phylogenetic relationship between these clades is not resolved in the molecular tree and lowly supported (Bootstrap: 65) in our analysis.

#### ﻿ Cylindrotoma americana

Taxon classificationAnimaliaDipteraCylindrotomidae

Osten Sacken, 1865, stat. reval.

2355E37A-0E02-58E7-A9F4-801B979449E0

Figs 6N, O, P , 7K

Cylindrotoma juncta Coquillett, 1900
Cylindrotoma splendens Doane, 1900
Cylindrotoma pallescens Alexander, 1931.

##### Non-type material examined.

**Canada** • 1 ♂; British Columbia, Cowichan Valley, Upper Carmanah Valley; 48.616°N, 124.733° W; alt. 95 m; 4 Jul. 1991 – 15 Aug. 1991; N. Winchester leg.; CKLP. • 1 ♀; British Columbia, Cowichan Valley, Upper Carmanah Valley; 48.67°N, 124.69° W; alt. 160 m; 4 Jul. 1991 – 15 Aug. 1991; N. Winchester leg.; CKLP. **Usa** • 1 ♂, 1 ♀; Alaska, Juneau; 58.37°N, 134.54° W; alt. 330 m; 14 Jun. 1988; F. Brodo leg.; CKLP.

##### Comparative diagnosis.

General appearance, colouration, antennal structure, and male and female terminalia are very similar to *C.distinctissima*. Differences: only the ventral margin of the inner gonocoxal lobe are sclerotised (Fig. 6N) (the lateral margin is also sclerotised in *C.distinctissima* (Fig. 6D, E). Sheath of aedeagus shorter and wider (Fig. 6P) than in *C.distinctissima* (Fig. 6L, M). Aedeagus does not narrow to the tip in this species, the lateral margin being almost straight (Fig. 6P) (in *C.distinctissima* the aedeagus clearly narrows to the tip, starting from around the middle in Japanese specimens (Fig. 6M). Spermathecae small (Fig. 7K), diameter ~ 0.07–0.1 of the width of sternite 8 (in *C.distinctissima* relatively large, 0.15–0.2 of the width of sternite 8).

For a detailed species description see Brodo (1967) under “*Cylindrotomadistinctissimaamericana*”.

##### Distribution.

Widely distributed species in Nearctic, known from Canada and USA (Alaska to Oregon and Colorado, in the east from Labrador and Newfoundland to Ontario and Pennsylvania) (Oosterbroek 2021).

#### ﻿ Cylindrotoma nigriventris

Taxon classificationAnimaliaDipteraCylindrotomidae

Loew, 1849

1F0BE019-A015-50FF-9AF5-6E507E25B426

Figs 9 , 10

##### Non-type material examined.

**Finland** • 1 ♂; Lohja, Karkola; 60.60841°N, 23.95901°E; alt. 125 m; 9 Jun. 2018; E. Viitanen leg.; CKLP. **Russia** • 1 ♂; Altai Republic, Ongudaysky District, Onguday, Seminsky Pass; 51.06°N, 85.59°E; alt. 1650 m; 27 Jun. 2016 – 30 Jun. 2016; N.E. Vikhrev leg.; CKLP. • 1 ♀; Altai Republic, Kupchegen Settlement, Chike-Taman Pass; 50.64477°N, 86.3117°E; alt. 1266 m; 28 Jun. 1964; E.P. Narchuk leg.; CKLP.

##### Supplementary description.

**Figure 9.**
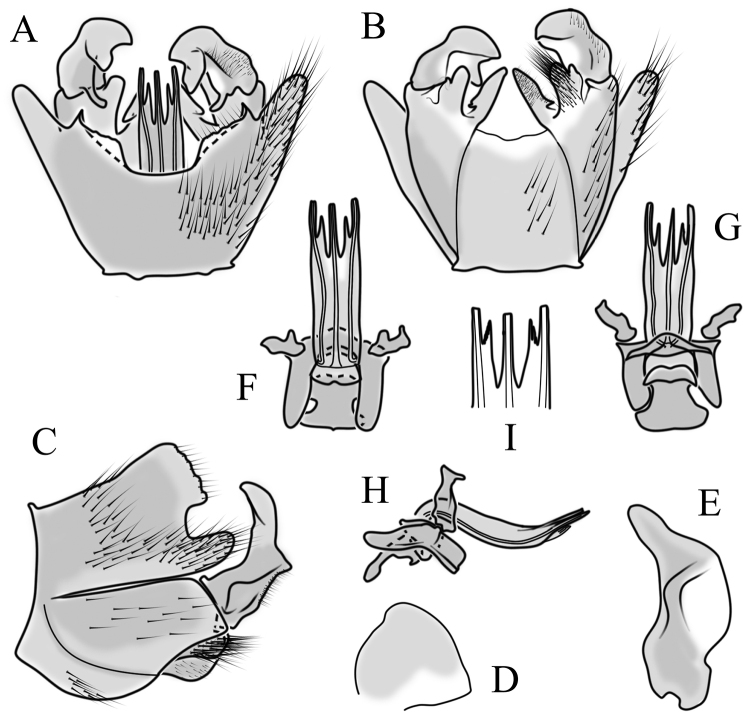
Male genital structures of *Cylindrotomanigriventris* Loew, 1849 **A** terminalia, dorsal view **B** terminalia, ventral view (aedeagus complex removed) **C** terminalia, lateral view **D** ventral lobe of the gonocoxite, lateral view **E** shape of the gonostylus, caudal view **F** aedeagus complex, dorsal view **G** aedeagus complex, ventral view **H** aedeagus complex, lateral view **I** tip of the aedeagus.

**Male terminalia**: Directed dorsally. Tergite 9 partly fused with gonocoxite (Fig. 9C). Posterior margin of tergite 9 with deep, U-shaped notch (Fig. 9A). Posterior edge of tergite 9 forming dorsal and ventral portion in lateral view (Fig. 9C). Ventral part produced caudally, forming finger-like lobe, covered by long hairs; dorsal portion wavy, formed by posterior margin of tergite 9 (Fig. 9C); lateral part of dorsal portion bent under tergite 9, forming a gently curved plate, covered with few fine setae, visible in caudal view. Gonocoxite fused with sternite 9 (Fig. 9B, C); sternite 9 sclerotised with few long hairs. Gonocoxite ventral lobe, laterally flattened, directed dorso-laterally, shape as in Fig. 9D; apical lobe of gonocoxite directed caudally, not inward as in *C.distinctissima* or *C.americana*; covered by long hairs, except small bare portion at base, next to gonostylus, visible in ventral view (Fig. 9B). Gonostylus undivided, twisted; base wide, with a small gently curved finger-like lobe directed inward; inner ventral part paler, slightly membranous; in caudal view medially with outgrowth ridge (Fig. 9E); gonostylus narrowing to tip in caudal view. Interbase small, without membranous median part (Fig. 9F). Aedeagus dorsoventrally flattened, gently curved dorsally (Fig. 9H), tip divided into three short, equal tubes in last 1/4 of its length (Fig. 9F, G). Spines on inner side of lateral branch of aedeagus large, distinct, forming spike-like outgrowth (Fig. 9I); in lateral view individual spine can be separated (Fig. 9H).

**Figure 10.**
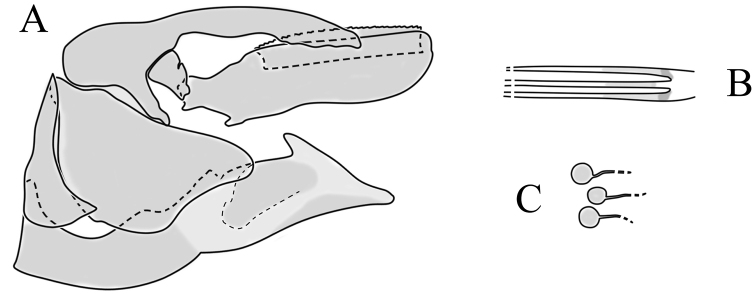
Female genital structures of *Cylindrotomanigriventris* Loew, 1849 **A** terminalia, lateral view **B** sperm ducts **C** Spermathecae.

**Female terminalia**: (Fig. 10A). Very similar to terminalia of *C.distinctissima* and *C.americana* stat. reval. The only clear difference is the sclerotisation of lateral sperm ducts (Fig. 10B), corresponding to the position of large spines on lateral branches of aedeagus (Fig. 9I). Spermathecae small (Fig. 10C), diameter ~ 0.08–0.12 × width of sternite 8 (in inner dorsal view).

##### Distribution.

Palearctic species, distributed from Finland to Far East Russia. Reported from Finland, Kazakhstan, Mongolia, and Russia: North European Russia, West Siberia (Altai Republic), East Siberia (Irkutsk Oblast), Far East Russia (Sakhalin Oblast, Primorsky Krai) (Oosterbroek 2021). The species was reported from the Altai Republic (Russia) by Soós and Oosterbroek (1992), but without any collection data, here we publish the first confirmatory record from the Altai Republic.

##### Comments.

Besides the apparent terminal differences in male specimens, the only distinct difference between *C.nigriventries*, *C.distinctissima*, and *C.americana* stat. reval. noted in our study, was the colouration of the scutellum, which is yellow in the latter two species, and with a median brown stripe or patch in *C.nigriventries*. Salmela and Autio (2007) and Gelhaus et al. (2007) note that these species also differ in the colouration of the abdomen (dark brown, almost black in *C.nigriventries*, and yellowish brown in *C.distinctissima*), however, some of the Japanese species of *C.distinctissima* have a very dark brown abdomen (Fig. 3A). The illustration of the female terminalia of *C.nigriventris* by Gelhaus et al. (2007: fig. 9) shows a high similarity to the drawing of the female terminalia of *C.distinctissima* by Peus (1952: fig. 21b), making the former suspect.

#### ﻿ Diogma caudata

Taxon classificationAnimaliaDipteraCylindrotomidae

Takahashi, 1960

6FC19634-5F8D-530A-AE79-9175863D5D60

**Figure 11.**
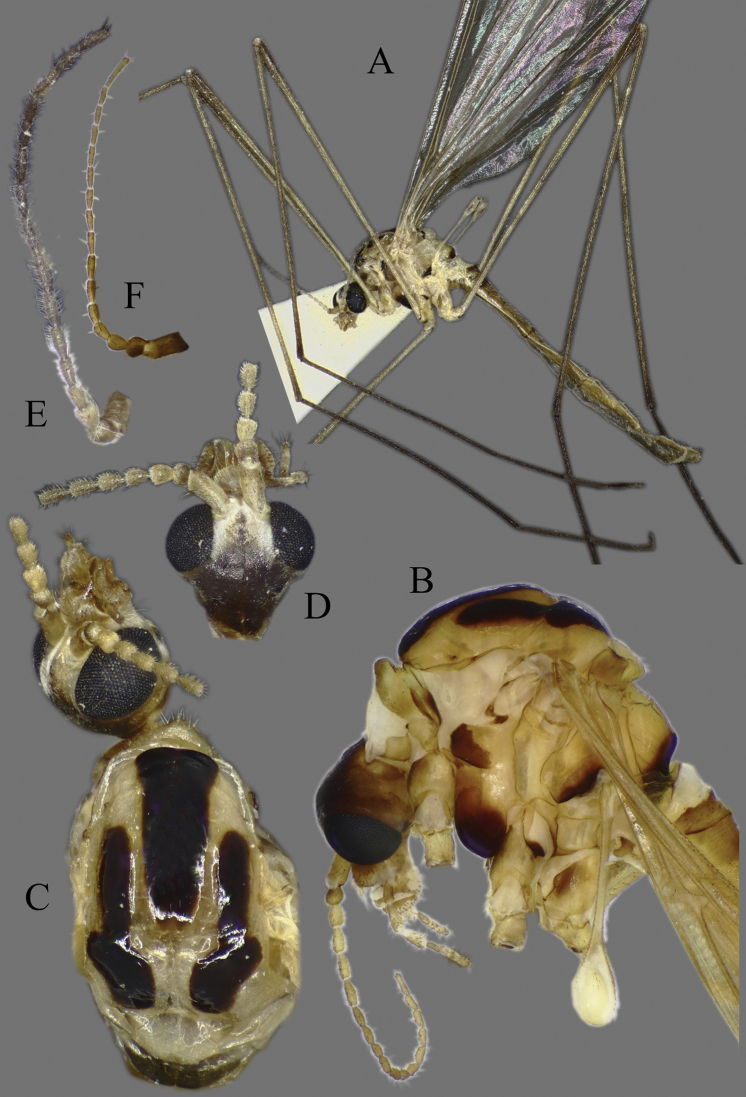
*Diogmacaudata* Takahashi, 1960 **A** habitus of the holotype male, lateral view (colouration of wings is artefact) **B** head and thorax of female, lateral view **C** head and thorax of female, lateral and dorsal views **D** head of female, dorsal view **E** antenna of holotype male **F** antenna of female.

**Figure 12.**
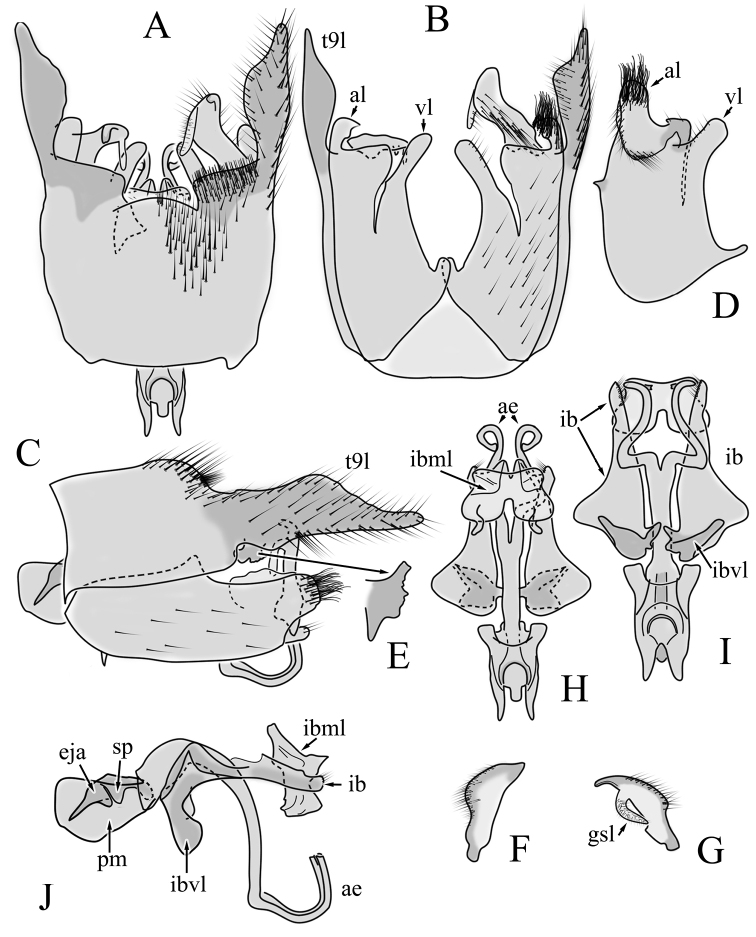
Male genital structures of *Diogmacaudata* Takahashi, 1960 **A** terminalia, dorsal view **B** terminalia, ventral view (aedeagus complex removed) **C** terminalia, lateral view **D** gonocoxite, inner lateral view **E** shape of heavily sclerotised lobe (lamina) of tergite 9 **F** shape of the gonostylus, caudal view **G** shape of the gonostylus, inner ventral view **H** aedeagus complex, dorsal view **I** aedeagus complex, ventral view **J** aedeagus complex, lateral view. Abbreviations: ae – aedeagus; al – gonocoxite apical lobe; eja – ejaculatory apodeme; gsl – lobe of gonostylus; ib – interbase; ibml – interbase median lobe; ibvl – interbase ventral lobe; pm – paramere; sp – sperm pump; t9l – tergite 9 lateral lobe; vl – gonocoxite ventral lobe.

**Figure 13.**
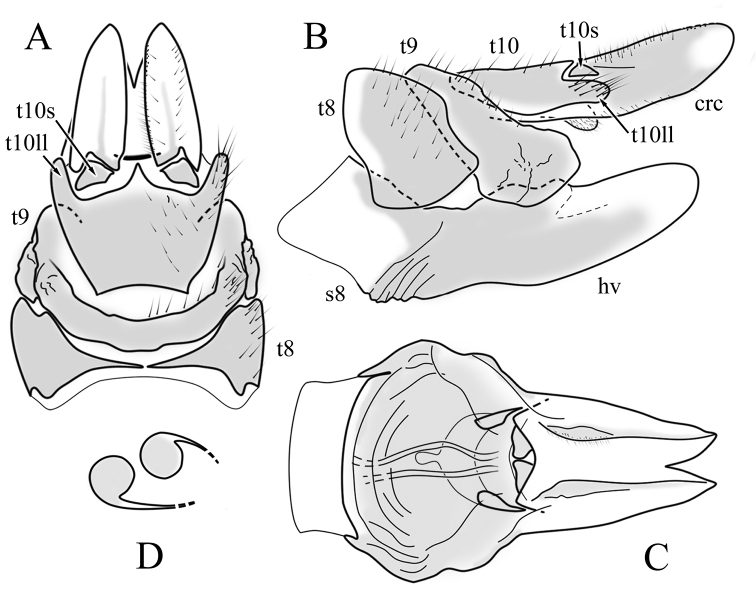
Female genital structures of *Diogmacaudata* Takahashi, 1960 **A** terminalia, dorsal view **B** terminalia, lateral view **C** sternite 8, hypogynial valve, genital fork, and sperm ducts, inner dorsal view **D** spermathecae. Abbreviations: crc – cercus; hv – hypogynial valve; t8 – tergite 8; t9 – tergite 9; t10 – tergite 10; t10s – tergite 10 triangular sclerite; t10ll – tergite 10 lateral lobe; s8 – stergite 8.

Figs 4B , 5C , 8B , 11 , 12 , 13

Diogma caudata in Takahashi 1960: 82–84: original description; Siitonen 1984: 203: faunistic record; Sidorenko 1999: 68–70: identification key, illustration, distribution; Oosterbroek et al. 2001: 122: distribution; Paramonov 2004a: 258: faunistic record; Paramonov 2005: 211: comparison; Mukkala et al. 2005: 7: faunistic record; Bartsch et al. 2005: red list status, faunistic record; Paramonov 2006: 888–889: identification key, illustration, distribution; Polevoi 2006: 96: faunistic records; Sandström 2008: red list status; Salmela 2008: 12: ecology; Salmela 2012a: 242: distribution; Salmela 2012b: 16: distribution; Salmela and Petrašiūnas 2014: 31: checklist; Nakamura 2014: 54: distribution.

##### Type material examined.

*Diogmacaudata* Takahashi: ***Holotype***: • ♂; Japan, Hokkaido, Mount Meakandake; 5 Jul. 1958; M. Takahashi leg.; ELUK.

##### Non-type material examined.

**Finland** • 3 ♂, 1 ♀; Kaavi, Kalalamminpuro; 63.11458°N, 28.67255°E; alt. 140 m; 20 Jun. 2008 – 17 Jul. 2008; J. Salmela leg.; LMM, CKLP. **Russia** • 1 ♂, 1 ♀; Arkhangelsk Oblast, Plesetsk District, Obozersky Settlement, around the settlement; 63.44231°N, 40.30789°E; alt. 100 m; 26 Jun. 1959; N.P. Krivosheina leg.; CKLP. • 1 ♂; Karelia Republic, Kon: 6909:550, Kondopoga District, Kivach Nature Reserve, spruce forest; 62.26766°N, 33.97975°E, alt. 42 m; 19 May. 1993 – 23 Jun. 1993; A.V. Polevoi leg.; window trap; ZIN. • 1 ♂; Karelia Republic, Karelia, Kon: 6982:570, Medvezhyegorsk Urban Settlement, 3 km NW Medvezhyegorsk City, point №6; 62.93364°N, 34.38467°E; alt. 130 m; 19 Jul. 2002; A.V. Polevoi leg.; ZIN. • 1 ♂; Perm Krai, Kungur Urban Okrug, Kungur City, forest station; 57.42881°N, 56.944206°E; alt. 219 m; 16 Jun. 1960; K.B. Borisova leg.; ZIN. • 1 ♂; Tuva Republic, Tandinsky District, north slope of Tannu-Ola mountains, near Chagytaj Lake; 50.99591°N, 94.6764°E; alt. 1500 m; 24 Jun. 1963; N.A. Violovich leg.; ZIN. **Sweden** • 2 ♂; Lule Lappmark, Kaltbacken bei Messaure; 66.67347°N, 20.32239°E; alt. 240 m; 23 Jun. 1969 – 26 Jun. 1969; • 1 ♀; same locality; 22 Jun. 1970 – 24 Jun. 1970; • 2 ♂; same locality; 23 Jun. 1971 – 30 Jun. 1971; • 31 ♂, 4 ♀; same locality; 12 Jun. 1972 – 13 Jul. 1972 / 21. Aug. 1972 – 28 Aug. 1972; • 2 ♂; same locality; 19 Jun. 1973 – 25 Jun. 1973; • 1 ♂, 1 ♀; same locality; 17 Jun. 1974 – 8 Jul. 1974; K. Müller leg.; ZFMK.

##### Redescription.

**Head**. Dorsally dark brown, ventrally brown. Frons with white pubescence noticeable only in dry specimens (Fig. 11C, D). Rostrum pale brown, short, without nasus, with few hairs. Mouthparts pale brown to brown (Fig. 11C, D). Palpus pale brown to brown, short, five segmented; last segment slightly longer than penultimate segment (Fig. 11B). Scape cylindrical, 2 × as long as pedicel; pedicel ovate, slightly darker than scape; flagellum 14-segmented, gradually darkening from base to tip; segments simple in both sexes, not expanded ventrally (Figs 4B, 11E, F); in male, first flagellomere as long as wide, remaining segments cylindrical; last segment 1.2–1.3 × as long as penultimate segment; flagellomeres with short, relative sparse whitish setae (sensilla), just slightly denser in ventral and lateral sides (Fig. 11E); in female, last flagellomere 1.8–2 × as long as penultimate; last 4–6 segments without sensilla (Figs 4B, 10F). Verticels black, shorter than length of flagellomere; usually one verticel in ventral surface and two or three in dorsal/dorsolateral sides, first segment with 4–6 verticels.

**Thorax**. General colouration yellowish brown with contrasting, shiny black markings. Mesonotum pale brown, greenish yellow in fresh, living specimen (Takahashi 1960), with three separated, broad, longitudinal black markings (Fig. 11C); several small yellow setae along pale strips. Scutellum yellow. Posterior part of mediotergite black. Anepisternum and katepisternum separated, both ventral parts dark brown to black. Ventral corner of laterotergite black. Additional small darker patch on posterior basalare, and on ventral part of meron. Coxa and trochanter yellowish, darker on anterior- dorsal parts (Fig. 11B); femur pale brown; tibia gradually darkening from pale brown to dark brown/black; tarsus uniformly black. Wing hyaline; veins pale brown to brown; pterostigma pale (Fig. 5B); three branches of M reaching wing margin; M1 in same level as M1+2; cell a2 narrow, > 7 × longer than wide (Fig. 5B); membrane with interference patterns, visible with dark background (Fig. 11A). Halter monochrome, yellow or pale brown.

**Abdomen**. Tergites and sternites pale brown to brown, tergite 8 and sternite 8 darker than others (Fig. 11A). Pleural parts greenish in living specimen (Takahashi 1960).

**Male terminalia**: Black, large, complex, directed caudally. Tergite 9 not fused with gonocoxite, partly cover gonocoxite (Fig. 12C); medial part rounded with small tuft of hairs (Fig. 12A); lateral lobe of tergite 9 greatly extended, complex; as long as basal part of tergite 9; ventral portion of lateral lobe elongated, finger-like in lateral view (Fig. 12C); lateral margin almost straight or weakly divergent in dorsal view (Fig. 12A, B); ventral base of lateral lobe with small, black, heavily sclerotised lobe (Fig. 12C, E) – named lamina by some authors – shape variable; posterior margin of tergite 9 between median round part and lateral lobe covered with dense short, blunt ended setae (Fig. 12A). Gonocoxite complex; apical lobe with dense hairs (Fig. 12B–D); ventral lobe round, almost bare (Fig. 12B, D); inner part of gonocoxite with basally directed lobe, with hairs on margin (Fig. 12D). Gonostylus simple, wider at middle (Fig. 12F); with finger-like membranous lobe on inner side, poorly visible in dry specimens (Fig. 12G). Aedeagus bifid; aedeagus with apical branches long, curved ventrally almost in right angle, then curved in right angle posteriorly, then turn dorsally in right angle in lateral view (Fig. 12J); dorsal lobe between interbases complex, sclerotised (Fig. 12H, J); interbase with ventral projection (Fig. 12J). Sperm pump and ejaculatory apodeme small (Fig. 12H–J), covered by parameres in lateral view (Fig. 12J).

**Female terminalia**: Brown, tip of cercus and hypopygial valve yellowish brown. Tergite 8 separated at middle by membranous area (Fig. 13A). Tergites 8 and 9 similar in size (Fig. 13B). Ventral corner of tergite 9 rugged (Fig. 13B). Triangular sclerite separated from tip of tergite 10 (Fig. 13A). Lateral lobes of tergite 10 elongated, with few longer hairs (Fig. 13A, B). Cercus and hypogynial valve simple, wide, blade-shaped, tips rounded (Fig. 13B). Dorsal apical surface of cercus rough, formed by few blunt, pyramid or round teeth (Fig. 13A, B). Base of sternite 8, weakly sclerotised, extended laterally at middle, with transverse creases (Fig. 13AC). Two round spermathecae present, duct curved (Fig. 13D). Lateral sclerite of genital fork elongated; two sperm ducts simple, without any clear markings (Fig. 13D).

##### Distribution.

Finland, Japan (Hokkaido I, Fig. 8B), Sweden, and Russia (Kareliya Republic, Perm Krai and Tuva) (Oosterbroek 2021). First records from Arkhangelsk Oblast, Russia.

##### Comments.

The species was initially described from Hokkaido, Japan. However, no additional Japanese data has been published, and the species was not found in the type locality in our study. The species later was reported from Finland, Sweden, and Russia (Karelia Republic, Perm Krai, and Tuva). Morphologically it is a well separated species from the close related *Diogmaglabrata*, but the Finnish specimens show only a small COI genetic difference from it and form a clade together with the West Palearctic *D.glabrata*. No significant morphological differences were found between the Finnish and Russian specimens and the Japanese holotype.

#### ﻿ Diogma glabrata

Taxon classificationAnimaliaDipteraCylindrotomidae

(Meigen, 1818)

62F83DDA-AC22-5F5B-983C-10AAF0063D1A

**Figure 14.**
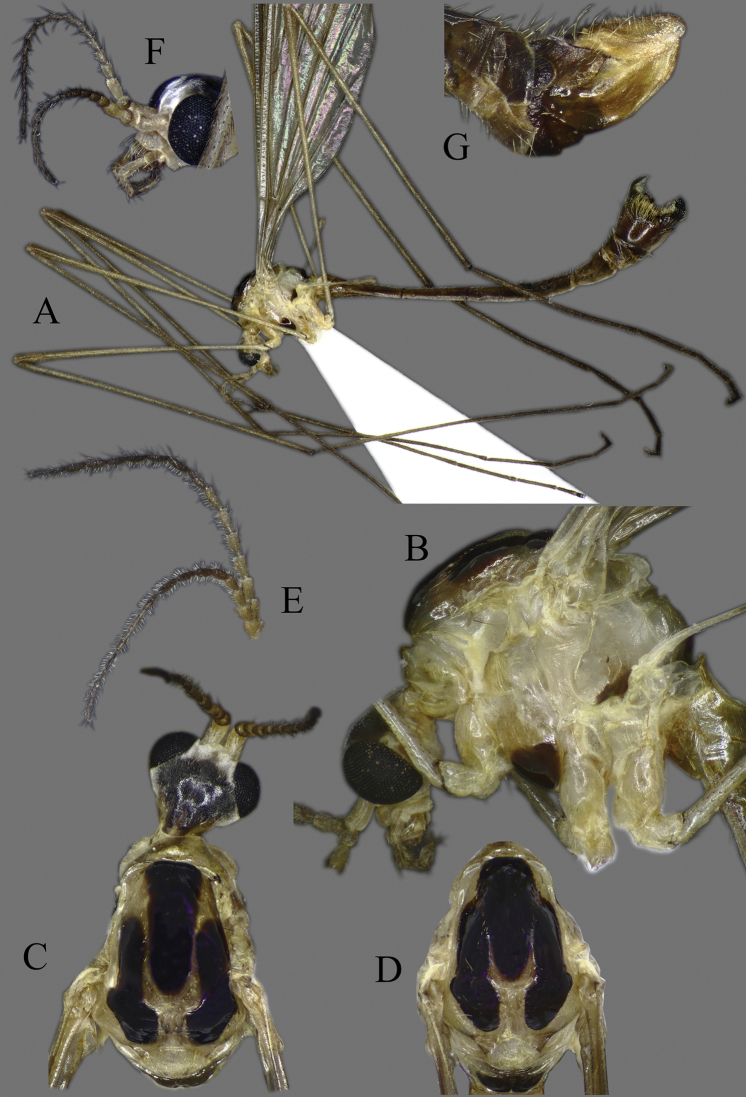
*Diogmaglabrata* (Meigen, 1818) **A** habitus of male, lateral view (colouration of wings is artefact) **B** head and thorax, lateral view **C** head and thorax, dorsal view of pale form **D** thorax, dorsal view – dark form **E** antenna of male, dorsal view **F** head of female, lateral view **G** female terminalia lateral view.

**Figure 15.**
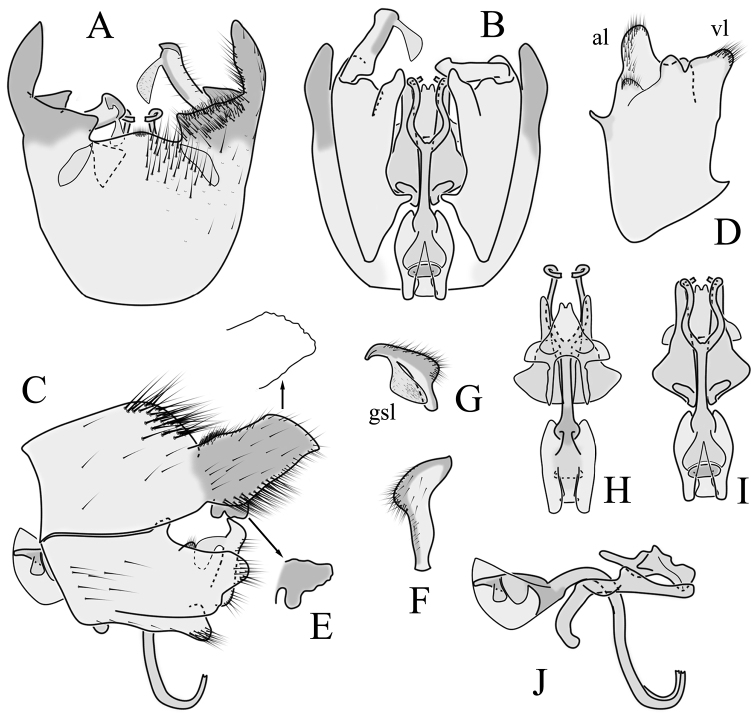
Male genital structures of *Diogmaglabrata* (Meigen, 1818) **A** terminalia, dorsal view **B** terminalia, ventral view **C** terminalia, lateral view **D** gonocoxite, inner lateral view **E** shape of heavily sclerotized lobe (lamina) of tergite 9 **F** shape of the gonostylus, caudal view **G** shape of the gonostylus, inner ventral view **H** aedeagus complex, dorsal view **I** aedeagus complex, ventral view **J** aedeagus complex, lateral view. Abbreviations: al – gonocoxite apical lobe; gsl – gonostylar lobe; vl – gonocoxite ventral lobe.

**Figure 16.**
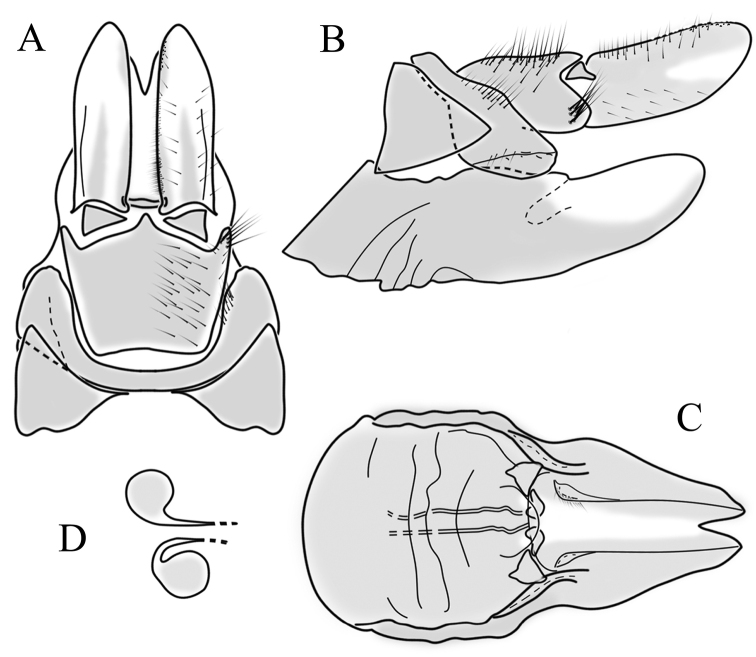
Female genital structures of *Diogmaglabrata* (Meigen, 1818) **A** terminalia, dorsal view **B** terminalia, lateral view **C** sternite 8, hypogynial valve, genital fork, and sperm ducts, inner dorsal view **D** spermathecae.

Figs 4C , 5C , 8B , 14 , 15 , 16

Phalacrocera megacauda in Alexander 1931: 349–350: original description.
Diogma glabrata megacauda in Alexander 1949: 196: comparison; Ishida 1955: 76: distribution; Takahashi 1960: 82: distribution, comparison, illustration; Alexander 1966: 122: distribution, faunistic records; Sidorenko 1999: 68–70: identification key, illustration, distribution.
Diogma glabrata (glabratamegacauda) in Paramonov 2006: 888–889: identification key, illustration, distribution.
Diogma glabrata megacauda , D.glabrata in Gelhaus et al. 2007: synonymy, comparison, ecology, distribution, illustration.
Diogma glabrata in Nakamura 2014: 54: distribution; Imada 2020: biology and ecology of larvae.

##### Non-type material examined.

**Belarus** • 2 ♂, 1 ♀; Brest Oblast; Kamenets District, Belavezhskaya Pushcha National Park; 52.58807°N, 23.81746°E; alt. 160 m; 4 Aug. 1961; E.P. Narchuk leg.; ZIN. **Denmark** • 1 ♂; ?Upilbo; 27 Jul. 1917; P. Nielsen leg.; USNM. **Estonia** • 1 ♀; Ida-Viru County, Narva-Jõesuu Town [Gungerburg]; 59.45°N, 28.03°E; 18 Jul. 1909; A.I. Chekini leg.; ZIN. **Finland** • 1 ♂, 2 ♀; Pihtipudas, Valkeispuro; 63.41082°N, 26.05336°E; alt. 170 m; 12 Jul. 2008 – 14 Aug. 2008; J. Salmela leg.; Malaise trap; LMM. • 1 ♂; Luvia, Niemenkyla; 61.39108°N, 21.56586°E; 12 m; 18 Jul. 2012; E. Viitanen leg.; CKLP. • 1 ♂; Mustasaari, Valassaaret; 63.43103°N, 21.07421°E; alt. 1 m; 2 Jul. 2019; E. Viitanen leg.; CKLP. • 1 ♀; Virolahti, Kurkela; 60.56858°N, 27.83847°E; alt. 8 m; 25 Jul. 2016; E. Viitanen leg.; CKLP. • 1 ♀; Fennia, Kb: 698:72, Ilomantsi, Tapionaho; 62.86016°N, 31.48371°E; alt. 190 m; 7 Jul. 1993 – 28 Jul. 1993; J.B. Jakovlev leg.; ZIN. • 1 ♀; Sotkamo, Iso-Matojarvi, Window trap №3, Kn: 7086:590; 63.86638°N, 28.85971°E; alt. 210 m; 1 Jul. 1997 – 14 Jul 1997; Kuussaari leg.; ZIN. **Japan** • 2 ♂; Hokkaido, Higashikawa, Asahidake, River Yukomabetsu; 43.65226°N, 142.80229°E; alt. 1120 m; 23 Jul. 2019; L.-P. Kolcsár leg.; CKLP. • 4 ♂, 1 ♀; Hokkaido, Shari, Shiretoko Pass; 44.05331°N, 145.10166°E; alt. 716 m; 26 Jul. 2019; L.-P. Kolcsár leg.; CKLP. • 1 ♂; Iwate, Hachimantai, Toshiti Spa; 39.94253°N, 140.86804°E; alt. 1344 m; 3 Aug. 2013; • 2 ♀; same locality; 5 Aug. 2015; D. Kato leg.; BLKU. • 4 ♂; Nagano, Otakimura, Mt. Ontake; 35.86894°N, 137.51421°E; alt. 1990 m; 22 Jul. 2016; D. Kato leg.; BLKU. • 1 ♂; Toyama, Toyama, Arimine Jurodani; 36.46063°N, 137.42198°E; alt. 1130 m; 28 Aug. 2009 – 1 Sep. 2009; • 1 ♀; same locality; 1 Sep. 2009 – 8 Sep. 2009; M. Watanabe leg; Malaise trap; BLKU. **Latvia** • 1 ♀; Dolesmuiža, Doles sala; 56.866°N, 24.2014°E; alt. 4 m; 20 Jul. 2018; L.-P. Kolcsár leg.; CKLP. • 1 ♂, 1 ♀; Skaistkalne, small stream; 56.411°N, 24.637°E; alt. 12 m; ; L.-P. Kolcsár leg.; birch-spruce forest; CKLP. **Russia** • 1 ♂; Altai Republic, Turochak District, near Artybash Settlement; 51.79299°N, 87.26535°E; alt. 430 m; 15 Jul. 2006; N.M. Paramonov leg.; ZIN. • 1 ♀; Amur Oblast, Shimanovsk District, Urochishche Samodon, 100 km W Svobodny City; 51.29°N, 126.83°E; alt. 320 m; 6 Aug. 1959; A.G. Zinovjev leg.; ZIN. • 1 ♂; Amur Oblast, Shimanovsk District, Simonovo Settlement, 75km W Svobodny City; 51.46°N, 126.98°E; alt. 305 m; 27 Jul. 1959; A.G. Zinovjev leg.; ZIN. • 1 ♂; Leningrad Oblast, Luga District, Jashhera Village; 58.89°N, 29.82°E; alt. 40 m; 23 Jul. 1963; A.A. Stackelberg leg.; ZIN. • 2 ♂, 1 ♀; Leningrad Oblast, Gobzhicy Village; 58.83°N, 30.13°E; 7 Jul. 1934 -16 Jul. 1934; A.A. Stackelberg leg.; ZIN. • 1 ♂, 2 ♀; Leningrad Oblast, Tolmachyovo Urban Locality; 58.86°N, 29.91°E, alt. 60 m; 16 Jul. 1935 – 26 Jul. 1935; A.A. Stackelberg leg.; ZIN. • 1 ♂; Leningrad Oblast, Kamenka River; 58.88°N, 29.76°E; alt. 65 m; 8 Jul. 1935 ; A.A. Stackelberg leg.; ZIN. • 2 ♂, 5 ♀, Leningrad Oblast, Vsevolozhsk District, Jukki Village; 60.11°N, 30.27°E; alt. 58 m; 13 Jul. 1931 – 22 Jul. 1933; A.A. Stackelberg leg.; ZIN. • 1 ♂; Leningrad Oblast, Vsevolozhsk District, Ostrovki Village; 59.81°N, 30.82°E; alt. 13 m; 21 Jun. 1906 – 22 Jun. 1906; G.G. Jakobson leg.; ZIN. • 1 ♂; Magadan Oblast, Magadan Urban Okrug, near Sokol Urban Settlement; 59.92°N, 150.71°E; alt. 177 m; 11 Jul. 2014 – 19 Jul. 2014; N.E. Vikhrev leg.; ZIN. • 2 ♀; Moscow Oblast, Naro-Fominsk District, Naro-Fominsk City, near Vostochnyy Community; 55.39094°N, 36.68878°E; alt. 195 m; 29 Jun. 2011; • 1 ♂; same locality, 29 Jun. 2014; D.I. Gavryushin leg.; ZIN. • 1 ♀; Moscow Oblast, Naro-Fominsky District, Vostochnyy [Oriental] settlement, within the settlement; 55.3741°N, 36.4984°E; alt. 205 m; 29 Jun. 2011; D.I. Gavryushin leg.; CKLP. • 1 ♂, Moscow Oblast, Naro-Fominsk, Nara River; 55.36075°N, 36.7404°E; alt. 174 m; 29. Jun. 2014; D.I. Gavryushin leg.; CKLP. • 1 ♀; Novgorod Oblast, Novgorod District, 1.5 km SE Glebovo Settlement; 58.54893°N, 31.83198°E; alt. 40 m; 2010; N.M. Paramonov leg.; ZIN. • 1 ♂; Primorsky Krai, Vladivostok City; 43.11553°N, 131.88548°E; alt. 20 m; 8 Aug. 2003; V.V. Sidorenko leg.; ZIN. • 1 ♂; Primorsky Krai, Chuguyevka District, Verchneussuri station; 44.067°N, 133.979°E; alt. 330 m; 30 Jul. 1979; A.G. Zinovjev leg.; ZIN. • 1 ♂; Primorsky Krai, Krasnoarmeysk District, Udegeyskaya Legenda National Park, apiary; 45.46052°N, 135.20451°E; alt. 700 m; 19 Jul. 2009; A.N. Ovtshinnikov leg.; ZIN. • 1 ♀; Primorsky Krai, Terney District, Terney Urban-type Settlement, Lower Serebryanka [Sanhobe] River; 45.09°N, 136.58°E; alt. 60 m; 6 Aug. 1941; K.J. Grunin leg.; ZIN. • 1 ♂; Primorsky Krai,Ussuriysk Urban Settlement, Gorno-Tajozhnoe Settlement, 25 km SE Ussuriysk; 43.69°N, 132.15°E; alt. 120 m; 3 Aug. 1963; E.P. Narchuk leg.; ZIN. • 1 ♀; Sakhalin Oblast, Severo-Kurilsky District, Kuril Islands, Paramushir Island, Rifovaya Bay; 50.4594°N, 156.0138°E; alt. 130 m; 30 Aug. 1999; A.S. Lelej, S.Y. Storozhenko leg.; ZIN. • 1 ♂; Sakhalin Oblast, Yuzhno-Kurilsk Urban Settlement, Kuril Islands, Kunashir Island, near Lagunnoe Lake; 44.062°N, 145.759°E; alt. 20 m; 25 Jul. 1955; N.A. Violovich leg.; ZIN. • 1 ♀; Sakhalin Oblast, Kuril Islands, Shikotan Island, near Malokurilskoye Village; 43.866°N, 146.827°E; alt. 30 m; 21 Aug. 1963; G.O. Krivoluckaja leg. ZIN. • 1 ♂; Sakhalin Oblast, Sakhalin Island, Yuzhno-Sakhalinsk City; 46.95°N, 142.73°E; alt. 50 m; 29 Jul. 1959; N.A. Violovich leg.; ZIN. • 1 ♀; Samara Oblast, Zhigulyovsk Urban Okrug, Zhiguli Nature Reserve, Bakhilova Polyana; 53.43543°N, 49.66252°E; alt. 45 m; 24 Jun. 2006; N.M. Paramonov leg.; ZIN. • 1 ♀; Tver Oblast, Udomlya District, 1,5 km NW Kaskovo Village; 57.98475°N, 35.03497°E; alt. 167 m; 19 Jul. 2017; A.G. Korobkov leg.; ZIN. • 1 ♂; Tver Oblast, Udomlya District, Moldino Settlement; 57.74807°N, 35.24965°E; alt. 155 m; 4 Jul. 2018; • 1 ♂; same locality; 5 Jul. 2018; A.G. Korobkov leg.; ZIN. • 1 ♂; Yaroslavl Oblast, Tutayev District, near former railway station Pustovo; 57.81438°N, 39.56016°E; alt. 122 m; 30 Jun. 2012; M.A. Klepikov leg.; pine forest, stream; ZIN.

##### Redescription.

**Head.** Dorsal part brown, ventral part yellowish brown (Fig. 14B, C). Frons with white to yellowish-grey pubescence, visible only in dry specimens (Fig. 14C, F). Rostrum yellowish brown, short without nasus; mouthparts pale brown to brown. Palpus pale brown to brown, 5 segmented; last segment slightly longer than penultimate segment (Fig. 14F). Scape cylindrical 1.8–2 × longer than pedicel; pedicel ovate; pedicel and scape same colour or pedicel slightly darker (Figs 4C, 14E, F); flagellum 14 segmented, gradually darkening from base to tip; flagellar segments simple in both sexes, not expanded ventrally; male flagellomere cylindrical, with short sparse whitish setae – sensilla, slightly denser in ventral and lateral sides; last segment 1.5–1.8 × longer than penultimate (Figs 4C, 14E); female flagellomeres oval to cylindrical, first 4–6 flagellomeres oval, rest of segments cylindrical, sometimes all segment elongated, cylindrical as in male; only first 8–10 flagellomeres with sensilla; last flagellomere 1.8–2.8 × longer than penultimate (Figs 4C, 14F); verticels black, shorter than length of flagellomere; generally one verticel in ventral surface and two or three in dorsal/dorsolateral sides of flagellomeres, first segment with 4–6 shorter verticels.

**Thorax.** General colouration yellowish with contrasting, shiny black markings. Pronotum yellow, middle part pale brown. Mesonotum yellow to pale brown with three separated black markings (Fig. 14C) or one big black patch (Fig. 14D). Scutellum yellow. Posterior part of mediotergite black (Fig. 14B). Anepisternum and katepisternum well separated; ventral part of katepisternum black; ventral part of anepisternum yellowish (Japan, Fig. 14B) or pale brown to brown (Finland, Russia). Ventral part of meron pale brown (Japan, Fig. 14B) or brown to black (Finland, Russia). Laterotergite black at ventral corner. Coxa and trochanter yellow, femur pale brown; tibia gradually darkening from pale brown to dark brown/black; tarsus uniformly black; tarsomeres each with one spur. Wing hyaline; veins pale brown to brown; pterostigma pale; three branches of M reaching wing margin, M_1_ at same level as M_1+2_, cell a_2_ narrow, > 7 × longer than wide (Fig. 5C); wing membrane with interference patterns, visible with dark background. Halter monochrome or knob darker, yellow to pale brown.

**Abdomen.** Tergites and sternites pale brown to brown, with paler longitudinal median line, poorly visible in dry specimens. Tergites and sternites 7 and 8 darker (Fig. 14A). Pleural parts yellow to greenish yellow in living specimen.

**Male terminalia.** Large, black directed caudally (Fig. 14A). Tergite 9 not fused with gonocoxite, partly cover gonocoxite (Fig. 15C); medial part of tergite 9 rounded, with small tuft of hairs (Fig. 15A); lateral lobe of tergite 9 greatly extended, complex, shorter than basal part of tergite 9; shape variable, rectangular to triangular in lateral view (Fig. 15C); margin wavy, especially in caudal end (Fig. 15C, see also Gelhaus et al. 2007: figs 12–15); weakly curved inward in dorsal view (Fig. 15A); ventral base of lateral lobe with small, black, heavily sclerotised lobe (named lamina by some authors) considerably variable in shape (Fig. 15E, see also, Gelhaus et al. 2007: figs 16–20, Takahashi 1960: figs 2, 3). Posterior margin of tergite 9 between median round part and lateral lobe with dense short, blunt ended setae (Fig. 15A). Gonocoxite complex; apical and ventral lobe tips rounded with hairs; inner part of gonocoxite with less defined lobe, directed apically with hairs on tip (Fig. 15D). Gonostylus simple, outer half wider (Fig. 15F), with triangular, membranous lobe at inner side, poorly visible in dry specimens (Fig. 15G). Sperm pump and ejaculatory apodeme small (Fig. 15H–J), covered by parameres in lateral view (Fig. 15J). Dorsal lobe between interbases complex, sclerotised (Fig. 15H, J); interbase with ventral projection (Fig. 15J). Aedeagus bifid, branches long, curved ventrally almost in right angle, then turned dorsally (Fig. 15J).

**Female terminalia.** Brown, tip of cercus and hypopygial valve yellowish brown (Fig. 14G). Tergite 8 separated at middle by membranous area (Fig. 16A). Ventral corner of tergite 9 weakly rugged and with hairs (Fig. 16B). Triangular sclerite separated from tip of tergite 10 (Fig. 16A). Lateral lobe of tergite 10 relatively small, with long hairs (Fig. 16A, B). Cercus and hypogynial valve simple, wide, blade-shaped, tips rounded (Fig. 16B). Dorsal apical surface of cercus rough, formed by few blunt pyramid or round teeth (Fig. 16A, B). Base of sternite 8 sclerotised (Fig. 16B), lateral margins almost straight in ventral and inner dorsal view (Fig. 16C), with transverse creases (Fig. 16B, C). Two round spermathecae present, duct curved (Fig. 16D). Lateral sclerite of genital fork triangular, two sperm ducts simple, without any clear markings (Fig. 16C).

##### Distribution.

Austria, Belgium, Czech Rep., Denmark, Estonia, Finland, France, Germany, Great Britain, Ireland, Japan: Hokkaido I, Honshu I, Korea (North Korea or South Korea), Lithuania, Luxembourg, Netherlands, Norway, Poland, Romania, Slovakia, Sweden, Switzerland, and Russia (North European territory, Central European territory (Yaroslavl Oblast), Far East (Amur Oblast, Primorsky Krai, Sakhalin Oblast (Kuril Is: Kunashir I) (Oosterbroek 2021).

First records from Belarus, Latvia, and Russia: Altai Republic, Amur Oblast, Novgorod Oblast, Magadan Oblast, Samara Oblast, and Kuril Islands (Shikotan I and Paramushir I). Occurrence data in Japan and surrounding areas are presented in Figure 8B.

##### Comments.

*Diogmaglabrata* is a relatively common species in Europe, with a similar distribution to *Cylindrotomadistinctissima*. However, it is rarer or seemingly absent from southern Europe (Kolcsár et al. 2018; Oosterbroek 2021). Alexander (1931) described the species *Phalacroceramegacauda* from Japan, based on the external morphological characters, without describing or illustrating the male terminalia. Later, Edwards (1938) designated a new genus, *Diogma*, for *Cylindrotomaglabrata*, which was previously included in *Liogma* by Osten Sacken (1859). Later, Alexander (1949) moved *Phalacroceramegacauda* to *Diogma* and mentioned it as a subspecies of *Diogmaglabrata*, without detailing the difference or the reason for transferring it to subspecies rank. Takahashi (1960) illustrated the structural difference of the ventral lobe of tergite 9, called “lamina”, between *Diogmaglabratamegacauda*, and *Diogmaglabrataglabrata*. This lobe’s morphological variability was discussed and illustrated in detail by Gelhaus et al. (2007). They concluded that the two *D.glabrata* subspecies did not significantly differ in stable features and synonymised *D.megacauda* with *D.glabrata*. After morphological comparisons of the Japanese specimens with the West Palearctic specimens, our conclusion is the same. Only the body colouration shows slight differences between the two groups, however, colour variation is common among different populations of Cylindrotominae species. In this study, the European specimens are found to be genetically separated from the Japanese specimens, and the *D.caudata* sequences joined the Finnish *D.glabrata* clade. Additional sequences are needed for both *Diogma* species, from different areas of their distribution ranges, to resolve this genetic contradiction.

#### ﻿ Diogma dmitrii

Taxon classificationAnimaliaDipteraCylindrotomidae

Paramonov, 2005

82A4EA5E-D4E2-5BA3-AF7B-3A62067D9120

Figs 17 , 18

##### Non-type material examined.

**Russia** • 1 ♂; Krasnodar Krai [Republic of Adygea, Maykopsky District], Khamyshki, Lagonaki Plateau; 44.009°N, 39.994°E; alt. 1700 m; 11 Jun. 2012; N.E. Vikhrev leg.; CKLP. • 1 ♀; Krasnodar Krai, Apsheronsky District, Mezmay Settlement, Kamyshanova polyana, Mezmaika River; 44.16989°N, 40.05180°E; alt. 1200 m; 13 Jun. 2004; N.M. Paramonov leg.; CKLP.

##### Supplementary description.

**Figure 17.**
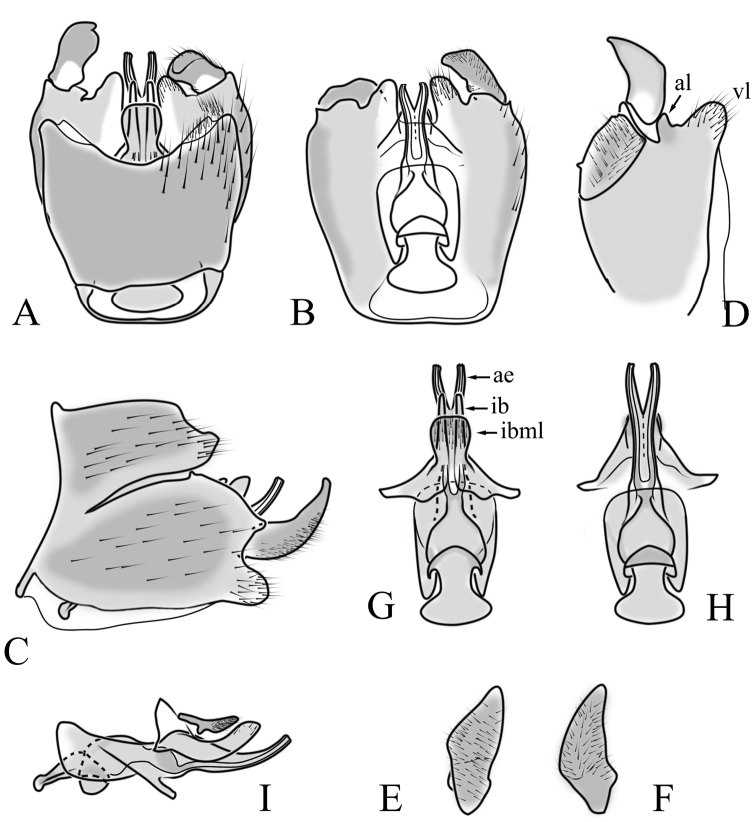
Male genital structures of *Diogmadmitrii* Paramonov, 2005 **A** terminalia, dorsal view **B** terminalia, ventral view **C** terminalia, lateral view **D** gonocoxite and gonostylus, inner lateral view **E** shape of the gonostylus, caudal view **F** shape of the gonostylus, inner dorsal view **G** aedeagus complex, dorsal view **H** aedeagus complex, ventral view **I** aedeagus complex,

**Male terminalia**: Medium sized and relatively simple, directed caudally. Tergite 9 fused with gonocoxite (Fig. 17C). Tergite 9 posterior margin convex in dorsal view (Fig. 17A), lateral lobe very small, triangular, ~ 1/4 ×total length of tergite 9 in lateral view (Fig. 17C); covered with relative long setae; posterior margin of tergite 9 with subhyaline, ventrally directed plate, next to lateral lobe; shape approximately triangular, covered with short pale setae (Fig. 17A). Sternite 9 fused with tergite 9 and gonocoxites, present as a narrow but continuous ring (Fig. 17A–C). Gonocoxite longer than tergite 9 in lateral view. Ventral lobe of gonocoxite well visible, without deep separation from gonocoxite (as in *D.caudata* and *D.glabrata*); inner half pale, partly membranous, covered by long pale hairs (Fig. 17B); apical lobe very small, mostly bare; inner part of gonocoxite forming a plate with hairs on all surface (Fig. 17D). Membranous area between gonocoxites reach base of ventral lobe (Fig. 17B). Gonostylus simple, without lobe in inner side; claw-like in lateral view (Fig. 17D); widened in middle length in caudal view (see Fig. 17E, F from different angles), with small, rounded bulge in inner ventral base (Fig. 17E). Sperm pump and ejaculatory apodeme large, partly covered by paramere in lateral view (Fig. 17I). Dorsal lobe between interbases dorso-ventrally flattened, posterior margin almost straight, covered by dense short hairs (Fig. 17G, I); interbase simple, with a few hairs, curved dorsally, without ventral projection, (Fig. 17I). Aedeagus bifid, branches short, slightly curved dorsally; base wide, evenly narrowing to tip in lateral view (Fig. 17I).

**Figure 18.**
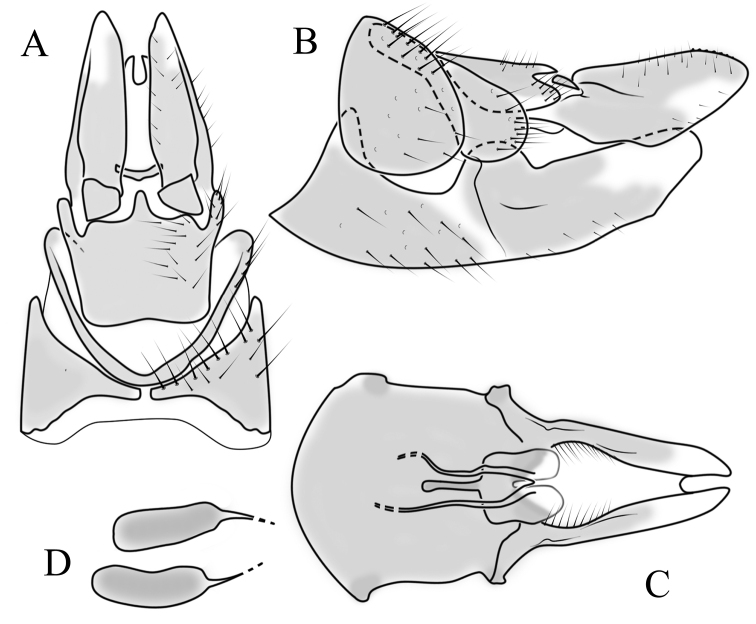
Female genital structures of *Diogmadmitrii* Paramonov, 2005 **A** Terminalia, dorsal view **B** terminalia, lateral view **C** sternite 8, hypogynial valve, genital fork, and sperm ducts, inner dorsal view **D** Spermathecae.

**Female terminalia**: Brown, tip of cercus and hypopygial valve yellowish brown. Tergite 8 separated at middle by membranous area (Fig. 18A). Tergite 8 larger than tergite 9 in lateral view (Fig. 18B). Ventral corner of tergite 9 not rugged, with few hairs (Fig. 18B). Triangular sclerite separated from tip of tergite 10, but close situated (Fig. 18A). Lateral lobes of tergite 10 finger-like with few long hairs (Fig. 18A). Cercus and hypogynial valve simple, wide, blade-shaped, tips rounded (Fig. 18B). Dorsal apical surface of cercus rough, formed by few, blunt pyramid teeth (Fig. 18B). Sternite 8 simple, without transverse creases (Fig. 18B, C). Two very large, elongated spermathecae present with duct almost straight (Fig. 18D). Two sperm ducts simple, without any clear markings, genital fork with a rod-shaped median part, posterior part pale (Fig. 18C).

##### Distribution.

Russia: North Caucasus (Krasnodar Krai, Karachay-Cherkessia Republic); Georgia, Turkey (Asiatic part: Manisa, Rize, Samsun, Trabzon) (Oosterbroek 2021).

#### ﻿ Liogma brevipecten

Taxon classificationAnimaliaDipteraCylindrotomidae

Alexander, 1932

6E701275-F164-5C6A-BB65-3E71F11A15F1

**Figure 19.**
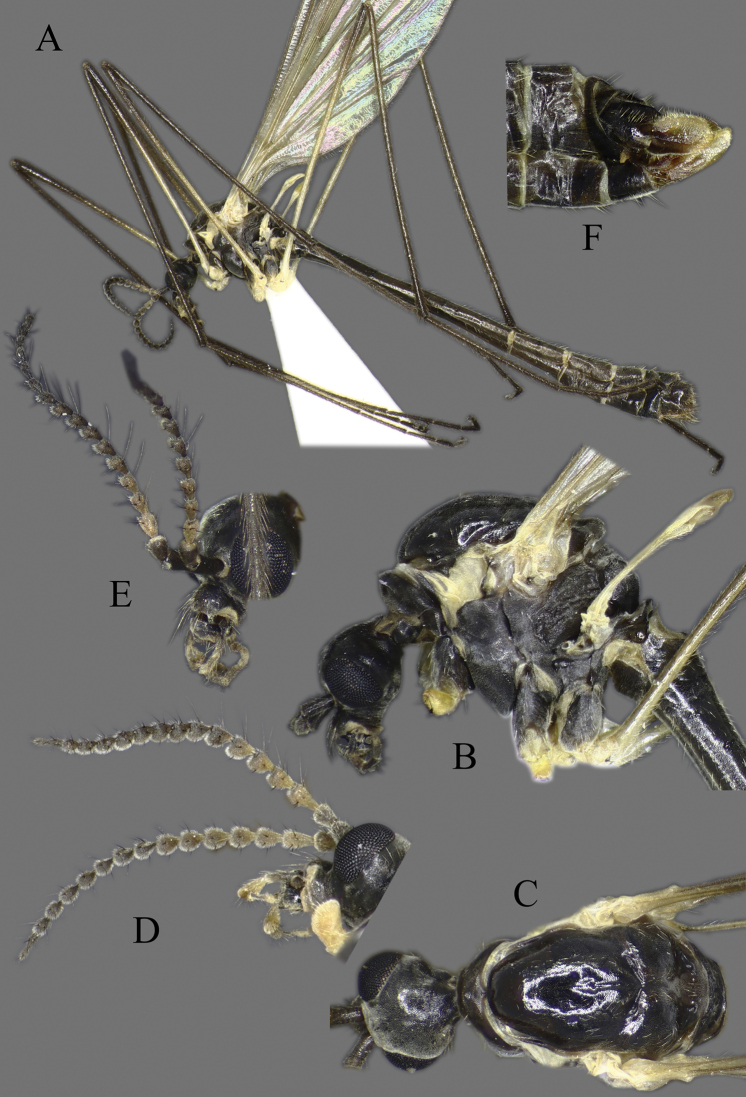
*Liogmabrevipecten* Alexander, 1932 **A** habitus of male, lateral view (colouration of wings is artefact) **B** head and thorax, lateral view **C** head and thorax, dorsal view **D** antenna of male **E** head of female, lateral view **F** female terminalia lateral view.

**Figure 20.**
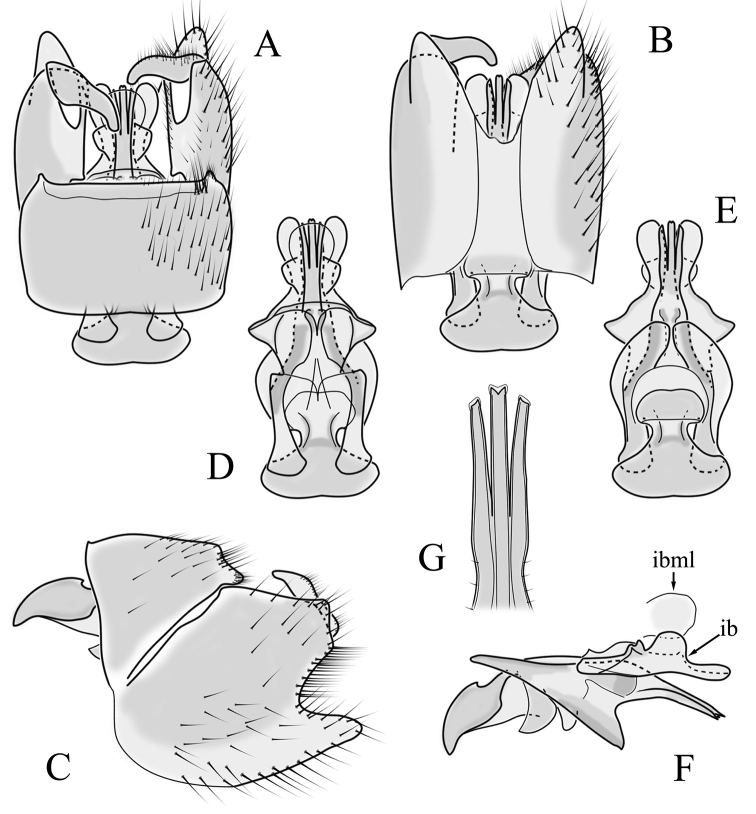
Male genital structures of Liogmabrevipecten Alexander, 1932 **A** terminalia, dorsal view **B** terminalia, ventral view **C** terminalia, lateral view **D** aedeagus complex, dorsal view **E** aedeagus complex, ventral view **F** aedeagus complex, lateral view **G** tip of the aedeagus. Abbreviations: ib – interbase; ibml – interbase median lobe.

**Figure 21.**
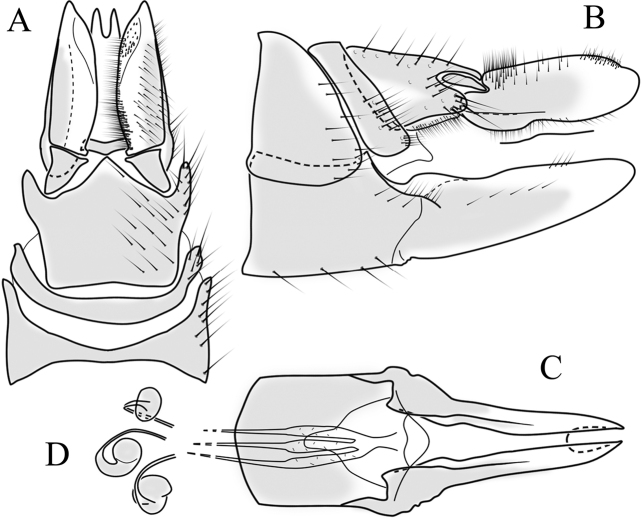
Female genital structures of *Liogmabrevipecten* Alexander, 1932 **A** aerminalia, dorsal view **B** terminalia, lateral view **C** sternite 8, hypogynial valve, genital fork, and sperm ducts, inner dorsal view **D** spermathecae.

**Figure 22.**
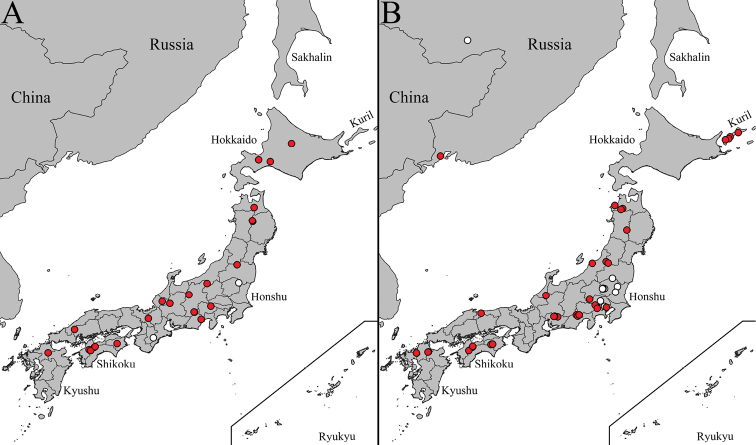
Occurrence data in Japan and surrounding areas of **A***Liogmabrevipecten* Alexander, 1932 **B***Liogmamikado* (Alexander, 1919). Red dots indicate locations of investigated specimens, white dots indicate approximate locations of literature data.

Figs 4E , 5E , 19 , 20 , 21 , 22A

Liogma brevipecten in Alexander 1932: 110–111: original descriptions; Ishida 1955: 75: distribution; Takahashi 1960: 84–85: distribution, additional description, faunistic records, illustration; Sidorenko 1999: 68–70: identification key, illustration, distribution; Nakamura 2001: 23–29: identification key, illustration, distribution, faunistic records; Paramonov 2006: 888–889: identification key; Nakamura 2014: 54: distribution; Imada 2020: biology and ecology of larvae.

##### Non-type material examined.

**Japan** • 1 ♂, 1 ♀; Aomori, Towada, Okuse, Tsutanuma Path; 40.59084°N, 140.95705°E; alt. 468 m; 23 May. 2014; • same locality; 1 Jun. 2014; D. Kato leg.; BLKU. • 1 ♂; Ehime, Kumakogen, small valley; 33.60489°N, 132.85584°E; alt. 580 m; 19 May. 2019; L.-P. Kolcsár leg.; CKLP. • 1 ♂; Ehime, Kumakogen, headwaters, stream; 33.56476°N, 132.93501°E; alt. 1387 m; 17 Jun. 2019; L.-P. Kolcsár leg.; CKLP. • 1 ♂; Ehime, Saijo, spring and mosses rocks; 33.75504°N, 133.15377°E; alt. 1480 m; 5 Jun. 2019; • 2 ♂; same locality; 16 Jun. 2019; L.-P. Kolcsár leg.; CKLP. • 1 ♂; Ehime, Wakayama, small waterfall; 33.71591°N, 133.10839°E; alt. 930 m; 18 May. 2019; L.-P. Kolcsár leg.; CKLP. • 1 ♀; Fukuoka, Soeda, rocky streem and moss covered cliff; 33.48309°N, 130.93289°E; alt. 900 m; 21 May. 2019; L.-P. Kolcsár leg.; CKLP. • 1 ♂; Fukui, Fukui, Mt. Ifuri; 35.96928°N, 136.4459°E; alt. 387 m; larva collected: 22 Apr. 2015, emerged: 3 May. 2015; Y. Imada leg.; CYI. • 1 ♂; Fukui, Ono, Aburazaka-touge; 35.87298°N, 136.82297°E; alt. 735 m; larva collected: 28 Apr. 2012, emerged: 3 May. 2012; M. Kato leg.; CYI. • 1 ♂; Hiroshima, Akiota, Yokogo; 34.59419°N, 132.14497°E; alt. 892 m; 18 May. 2015; D. Kato leg.; BLKU. • 1 ♂; Hokkaido, Chitose, Komukara-toge, small stream; 42.837°N, 141.7505°E; alt. 55 m; 14 Jun. 2015 – 27 Jun. 2015; N. Kuhara leg.; Malaise-trap; EUMJ. • 1 ♂; Hokkaido, Kamikawa, Aizankei; 43.73521°N, 142.7923°E; alt. 762 m; 25 Aug. 2015; M. Kato leg.; CYI. • 1 ♂, 1 ♀; Hokkaido, Sapporo, Minami-ku, Jozankei, trail of Mt. Sapporo; 42.92392°N, 141.17688°E; alt. 450–860 m; 23 Jun. 2014; D. Kato leg.; BLKU. • 1 ♀; Iwate, Hachimantai, Matsuoyoriki; 39.89958°N, 140.89155°E; alt. 1200 m; larva collected: 14 Jun. 2014, emerged: 4 Jul. 2014; Y. Imada leg.; CYI. • 2 ♂; Iwate, Hachimantai, Toshiti Spa; 39.94253°N, 140.86804°E; alt. 1344 m; 3 Aug. 2013; • 4 ♂; same locality; 15 Jul. 2014; • 1 ♀; same locality; 5 Aug. 2014; D. Kato leg.; BLKU. • 1 ♂; Kyoto, Kyoto, Kibune; 35.13681°N, 135.76622°E; alt. 458 m; larva collected: 3 Apr. 2016, emerged: 1 May. 2016; • 1 ♀; same locality; larva collected: 13 May. 2016, emerged: 20 May. 2016; Y. Imada leg.; CYI. • 1 ♂; Nagano, Ichiromata, Mt. Jonen; 36.3°N, 137.76°E; 27 Jul. 1951; Inoue leg.; USNM. • 1 ♂; Nagano, Iida, Jabora-rindo; 35.44865°N, 138.00905°E; alt. 1337 m; larva collected: 27 Apr. 2014, emerged: 6 May. 2014; M. Kato leg.; CYI. • 1 ♂; Nagano, Iida, Shirabiso-touge; 35.43801°N, 138.03053°E; alt. 1830 m; larva collected: 19 Oct. 2015, emerged: 18 Dec. 2015; Y. Imada leg.; CYI. • 1 ♀; Nagano, Sakae, Akiyama-gou; 36.85447°N, 138.64803°E; alt. 1125 m; larva collected: 3 May. 2015, emerged: 14 Apr. 2015; Y. Imada leg.; CYI. • 1 ♀; Shizuoka, Shizuoka, Tsudono; 35.08929°N, 138.35618°E; alt. 175 m; 3 May. 2015; M. Kato leg.; CYI. • 1 ♂; Tokushima, Naka, Mt. Takashiro, Kisawamura; 33.90468°N, 134.23315°E; alt. 1300 m; 16 May. 2016; M. Kato leg.; CYI. • 1 ♂; Tokushima, Yamagata, Yonezawa, Shirabu-onsen; 37.77646°N, 140.11964°E; alt. 888 m; larva collected: 19 Oct. 2013, emerged: 25 Apr. 2014; M. Kato leg.; CYI. • 1 ♀; Yamanashi, Koshu, Enzankamihagihara, Kaminichikawa Pass; 35.7316°N, 138.8321°E; alt. 1580 m; 8 Jul. 2014; D. Kato leg.; BLKU.

##### Redescription.

**Head.** Black with greyish pubescence (Fig. 19B–E). Rostrum short without nasus, but with patch of hairs (Fig. 19B, E); rostrum and mouthparts dark brown to black. Palpus pale brown to black, five segmented; first two segments always darker (Fig. 19E, D); last segment 1.3–1.5 × longer than penultimate. Scape cylindrical, 2 × as long as pedicel, and usually darker than pedicel; pedicel ovate; flagellum 14 segmented, gradually darkening to tip (Figs 4E, 19D, E); flagellomeres expanded ventrally in both sexes, more prominent in male (Figs 4E, 19D); only flagellomeres 2 or 3–9 extended evidently ventrally in female (Figs 4E, 19E), remaining segments elongated; last flagellomere cylindrical in both sexes; extended flagellomeres covered with dense whitish sensilla, denser on ventral side; six verticels on each flagellomere, two long verticels on dorsal surface, two verticels in lateral surface, two shorter on ventral side; first flagellomeres always bearing additional 2–4 verticels; second to sixth flagellomeres sometimes with additional one or two shorter verticels on dorsal surface.

**Thorax.** Uniformly black with weak greyish pruinosity, except pleural area, base of wing, and halter which yellowish especially in living specimens (Fig. 19B). Scatter pale short hairs on mesonotum present, forming two lines. Anterior 1/3–1/2 of mediotergite and anterior half of pleurotergite with creases and rugoses (Fig. 19B). Trochanter yellow; femur gradually darkening apically, basal part yellowish, apically dark brown; tibia brown to dark brown; tarsus uniformly brown to black (Fig. 19A). Wing pale, tinged with brown; pterostigma brown, well defined; veins brown; three branches of M reaching wing margin; M_1_ at same level as M_1+2_, cell a_2_ less than 6 × longer than wide (Fig. 5E); membrane with interference patterns, visible with dark background (Fig. 19A).

**Abdomen.** Dark brown to black (Fig. 19A). Pleura yellowish especially in females and living specimen.

**Male terminalia**: Uniformly dark brown to black, relatively small, directed caudally (Fig. 19A). Tergite 9 fused with gonocoxite (Fig. 20C); caudal margin straight, without prominent outgrowth, only a small lateral lobe present at lateral corner (Fig. 20A, C). Sternite 9 membranous (Fig. 20B). Gonocoxite large, 1.5–1.6 × longer than tergite 9, with long ventral lobe (Fig. 20B, C); inner surface of gonocoxite simple, without lobe (Fig. 20A). Gonostylus simple, tapering to distal end. Aedeagal complex large, 1.2–1.3 × longer than gonocoxite (Fig. 20D–F); ejaculator apodeme and sperm pump large, together 1/2 × length of aedeagal complex, not covered by parameres in lateral view (Fig. 20F); tip of interbase finger-like, with round lobe dorsally in lateral view (Fig. 20F); interbase wide and rounded in dorsal view (Fig. 20D); dorsal lobe between interbases globular, membranous (Fig. 20D, F); aedeagus trifid, median branch slightly longer (Fig. 20G); sperm ducts branching from elongated portion of sperm pump, branching area dark (Fig. 20F).

**Female terminalia**: Brown to black, end of cercus and hypogynial valve yellowish (Fig. 19F). Tergite 8 three times larger than tergite 9 (Fig. 21B), not divided medially (Fig. 21A). Tergite 9 narrow band-shaped in lateral view (Fig. 21B). Triangular sclerite relatively large, 1/4 ×length of tergite 10; sclerite separated from tip of tergite 10; lateral lobe of tergite 10 medium sized, with few long hairs (Fig. 21A, B). Cercus and hypogynial valve broad, blade-like, tips rounded (Fig. 21B). Cercus on dorsal surface close to apical end with small notch; area before notch rough, with short and dense setae, and with few short sharp teeth (Fig. 21A, B); ventral margin of cercus before mid-length with notch (Fig. 21B). Common spermathecal duct present after genital opening; sperm ducts extended, inner wall rugose (Fig. 21C); three spermathecae laterally elongated, base of duct wide, curved, suddenly tapering suddenly (Fig. 21D).

**Distribution.** Japan (Honshu I and Kyushu I) and Russia (Far East: Sakhalin Oblast) (Oosterbroek 2021). First records from Japan: Hokkaido I and Shikoku I (Fig. 22A).

##### Comments.

This species differs from the closely related *Liogmaserraticornis* in details of the antennae, male and female terminalia, and colouration, though these are slight. Both sexes of this species can be separated from *L.serraticornis* based upon the first flagellomere length. It is always longer than the second flagellomere in *L.serraticornis* (Fig. 4F) and similar size in *L.brevipecten* (Fig. 4E). The ventral extensions of the flagellomeres of male *L.brevipecten* are relatively short and shout (Fig. 4E), while *L.serraticornis* has more elongated flagellomeres (Fig. 4F). The pedicel’s colouration and wing venation characters mentioned by Takahashi (1960) were not useful for species separation here because these characters show high variability levels. The two species differ in details of male and female terminalia: the ventral lobe of the gonocoxite has several pale spine-like setae in *L.serraticornis* (Fig. 29B, C), whereas *L.brevipecten* is without these spine-like setae (Fig. 20B, C); the middle branch of the aedeagus is longer than the lateral ones in *L.brevipecten* (Fig. 20G) but shorter than the lateral branches in *L.serraticornis* (Fig. 29G). The female terminalia of *L.brevipecten* is narrow and long in dorsal view (Fig. 21C), but widens ventrally in *L.serraticornis* (Fig. 30C). In *L.serraticornis* the lateral lobes of tergite 10 are 2 × longer than wide (Fig. 30B), but only as long as wide in *L.brevipecten* (Fig. 21B). Inner genital structures also show differences among the species in the spermathecae shapes (see Figs 21D, 30D) and sperm ducts. The base of the sperm duct is readily discernible in *L.brevipecten* (Fig. 21C), while it is short or very poorly discernible in *L.serraticornis* (Fig. 30C); and the sperm ducts house three inflated areas the shape of golf tees in *L.serraticornis* (Fig. 30C), but these are much less well developed in *L.brevipecten* (Fig. 21C).

#### ﻿ Liogma mikado

Taxon classificationAnimaliaDipteraCylindrotomidae

(Alexander, 1919)

F9511990-009B-516C-993E-0AF41571BD3C

**Figure 23.**
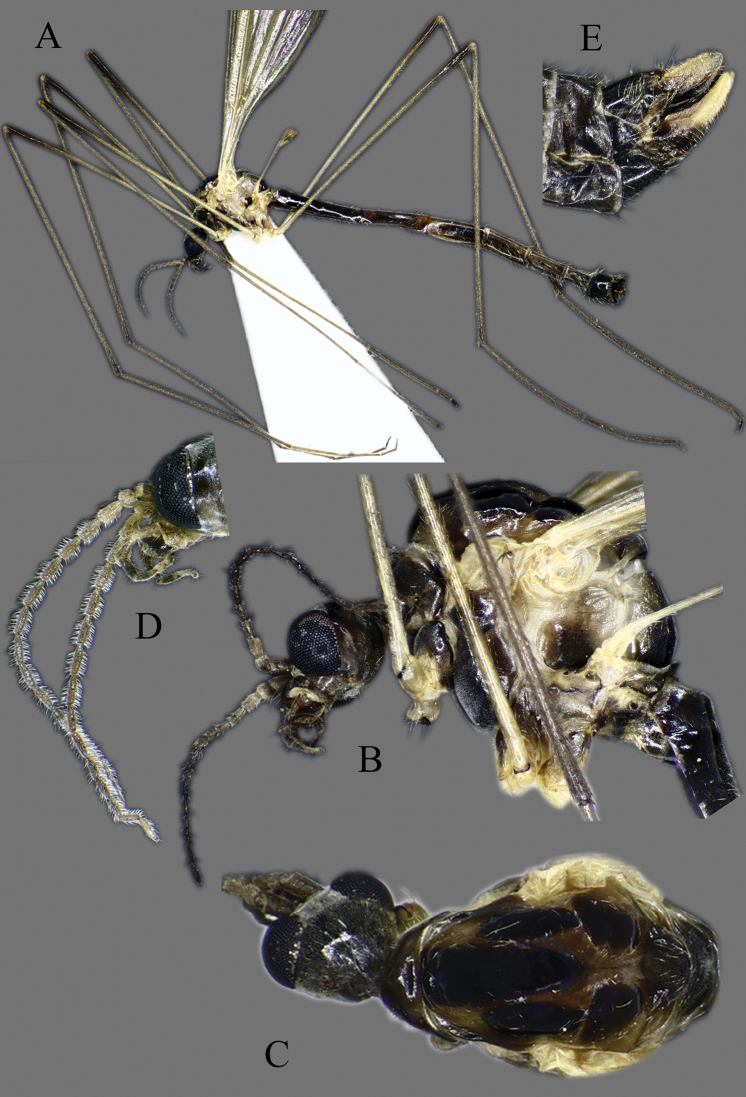
*Liogmamikado* (Alexander, 1919) **A** habitus of male, lateral view (colouration of wings is artefact) **B** head and thorax of female, lateral view **C** head and thorax, dorsal view **D** head of male, dorsal view **E** female terminalia lateral view.

**Figure 24.**
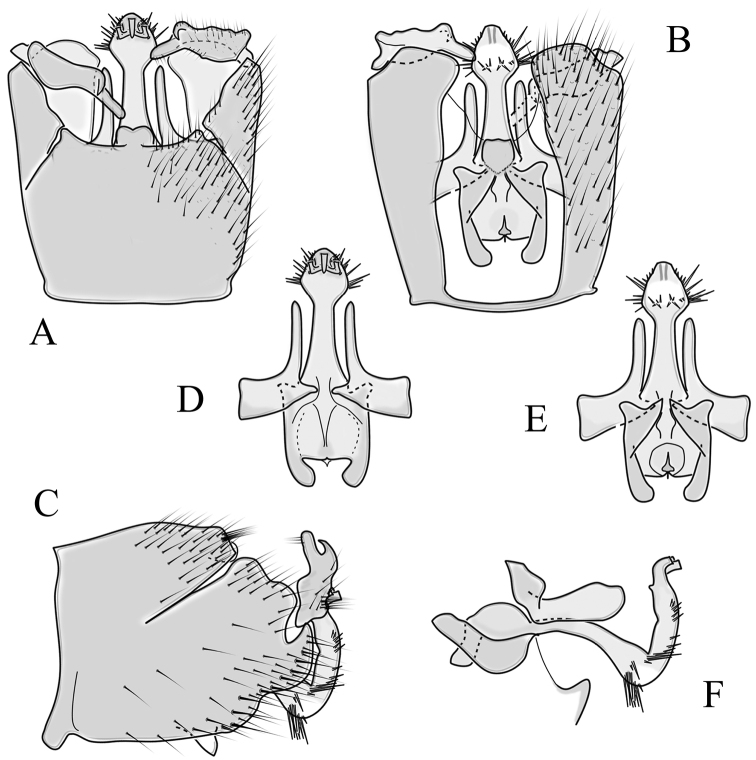
Male genital structures of *Liogmamikado* (Alexander, 1919) **A** terminalia, dorsal view **B** terminalia, ventral view **C** terminalia, lateral view **D** aedeagus complex, dorsal view **E** aedeagus complex, ventral view **F** aedeagus complex, lateral view.

**Figure 25.**
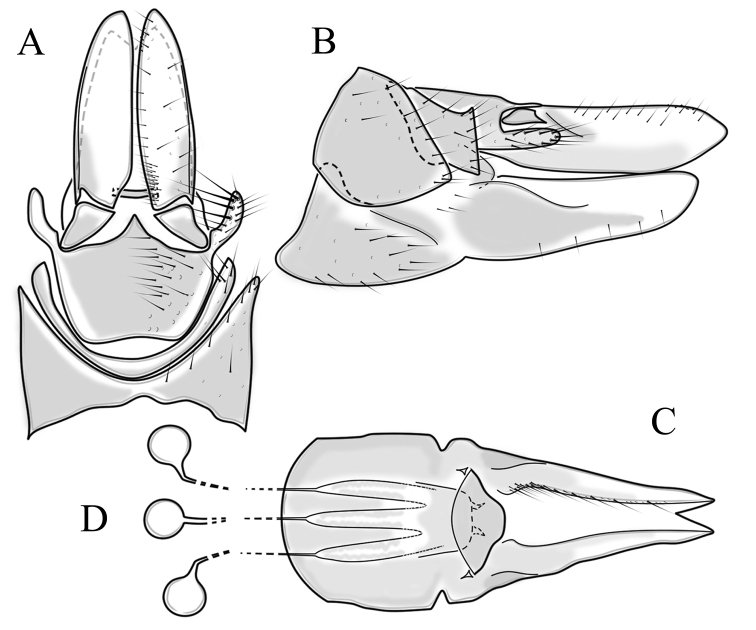
Female genital structures of *Liogmamikado* (Alexander, 1919) **A** terminalia, dorsal view **B** terminalia, lateral view **C** sternite 8, hypogynial valve, genital fork, and sperm ducts, inner dorsal view **D** spermathecae.

Figs 4D , 5D , 22B , 23 , 24 , 25

Phalacrocera mikado in Alexander 1919: 346: original description; Alexander 1928: 10: distribution, illustration; Alexander 1953a: 57: faunistic record; Ishida 1955: 77: distribution.
Liogma mikado in Takahashi 1960: 85–90: new combination, distribution, faunistic records, larva and pupa description, illustrations; Sidorenko 1999: 68–70: identification key, illustration, distribution; Nakamura 2001: 23–29: identification key, illustration, distribution, faunistic records; Paramonov 2004b: 69: faunistic record; Paramonov 2006: 888–889: identification key, distribution; Nakamura 2014: 54: distribution; Kato and Suzuki 2017: 16: distribution; Paramonov 2019: 120: faunistic data; Imada 2020: biology and ecology of larvae; Kim and Bae 2020: distribution.

##### Type material examined.

*Phalacroceramikado* Alexander: ***ALLOTYPE*** ♂: • **Japan**, Tokyo, Tokyo metropolis, 1919.04.?, leg. R. Takahashi (USNM).

##### Non-type material examined.

**Japan** • 2 ♀; Aichi, Toyota, Kawashimo, triburary of Yahagi River; 35.20376°N, 137.3012°E; alt. 140 m; 4 May. 2014; D. Kato leg.; BLKU. • 1 ♀; Aichi, Seto, Iwaya-cho, near Iwayada Park; 35.23957°N, 137.15084°E; alt. 300 m; 4 May. 2016; D. Kato leg.; BLKU. • 1 ♀; Aichi, Seto, Minamiazuma; 35.223213°N, 137.1131°E; alt. 150 m; 5 May. 2014; D. Kato leg.; BLKU. • 3 ♀; Aomori, Hirosaki, Koguriyama, Inekari River; 40.53658°N, 140.48701°E; alt. 170 m; 28 May 2013; • 1 ♂; same locality; 31 May. 2013; D. Kato leg.; BLKU. • 1 ♂; Aomori, Fukaura, Mt. Takanio; 40.68993°N, 140.10285°E; alt. 140 m; 11 May. 2014; D. Kato leg.; BLKU. • 1 ♂; Aomori, Hirosaki, Soma Path; 40.49479°N, 140.40231°E; alt. 392 m; 31 May. 2013; D. Kato leg.; BLKU. • Ehime, Kumakogen, River Myogadani springs, 1275 m, 33.55808°N, 132.93805°E, 2019.05.19, 2 ♂ 1 ♀, L.-P. Kolcsár leg.; CKLP. • 5 ♂, 11 ♀; Ehime, Wakayama, Mount Ishizuchi; 33.76491°N, 133.12948°E; alt. 1600 m; 5 Jul. 2019; L.-P. Kolcsár leg.; CKLP. • 1 ♂; Ehime, Wakayama, small waterfall and stream; 33.74519°N, 133.13714°E; alt. 1305 m; 18 May. 2019; L.-P. Kolcsár leg.; CKLP. • 2 ♀; Ehime, Wakayama, small waterfall; 33.71591°N, 133.10839°E; alt. 930 m; 18 May. 2019; L.-P. Kolcsár leg.; CKLP. • 2 ♂; Fukuoka, Fukuoka, Sawara-ku, Itaya, Mt. Sefuri; 33.43811°N, 130.36673°E; alt. 970 m; 2 May. 2015; • 1 ♂; same locality; 13 May. 2015; D. Kato leg.; BLKU. • 1 ♂; Fukuoka, Miyako, Saigawa-Hobashira, Notoge Pass; 33.49565°N, 130.96156°E; alt. 740 m; 22 Apr. 2016; D. Kato leg.; BLKU. • 1 ♀; Fukuoka, Soeda, rocky streem and moss covered cliff; 33.48309°N, 130.93289°E; alt. 900 m; 21 May. 2019; L.-P. Kolcsár leg.; CKLP. • 2 ♂; Ishikawa, Hakusan, near to Hakusan National Park; 36.25869°N, 136.72558°E; alt. 678 m; 27 May. 2015; M. Kato leg.; CYI. • 1 ♀; Iwate, Nishiwaga, Mahirudake; 39.46511°N, 140.69365°E; alt. 900 m; 19 Jun. 2015; Y. Imada leg.; CYI. • 1 ♂; Niigata, Echigo, Sugatani, Kitakanbara; 37.84°N, 139°E; 8 May. 1955; H. Koike leg.; USNM. • 1 ♀; Saitama, Ogano, Mt. Futago; 36.06994°N, 138.86753°E; alt. 942 m; larva collected: 28 Nov. 2014, emerged 15 Dec. 2014; M. Kato leg.; CYI. • 1 ♀; Shizuoka, Aoi-ku, Umegashima, Akamizu; 35.27455°N, 138.32731°E; alt. 680 m; larva collected: 8 Jan. 2007, emerged: 22 Feb. 2007; leg. Y. Sato EUMJ. • 9 ♂; Shizuoka, Shizuoka, Hatanagi; 35.2976°N, 138.21557°E; alt. 828 m; 12 May. 2013; M. Kato leg.; CYI. • 1 ♂; Shizuoka, Shizuoka, Abenoootaki; 35.30031°N, 138.35084°E; alt. 930 m; larva collected: 15 Jan. 2014, emerged: 19 Apr. 2014; M. Kato leg.; CYI. • 8 ♂, 1 ♀; Shizuoka, Ikawa-touge; 35.24094°N, 138.28156°E; alt. 1471 m; 10 May. 2015; M. Kato / Y. Imada leg.; CYI. • 1 ♂; Tokushima, Awa, Mt. Tsurugi; 33.87°N, 134.11°E; 30 May. 1950; Issiki-Ito leg.; USNM. • 1 ♂; Tokushima, Mima, Koyadaira; 33.87543°N, 134.09571°E; alt. 1340 m; 30 Apr. 2016; D. Kato leg.; BLKU. • 1 ♂; Tokushima, Miyoshi, Higashiiya-Sugeoi, near Nagoro Dam; 33.85182°N, 134.0234°E; 29 Apr. 2016; D. Kato leg.; BLKU. • 2 ♂; Tokyo, Mt. Mitake; 35.78°N, 139.14°E; 10 May. 1931; B. Oda leg.; USNM. • 1 sex unknown; Tokyo, Mt. Takao; 35.62°N, 139.24°E; alt. 300–600 m; 7 May. 1922; Esaki leg.; USNM. • 1 ♀; Tokyo, Tokyo, Akiruno, rocky river and stream; 35.74766°N, 139.18466°E; alt. 288 m; 11 May. 2019; L.-P. Kolcsár leg.; CKLP. • 1 ♂; Tottori, Mt. Daisen; 35.38°N, 133.54°E; 7 Jun. 1930; Hibi leg.; USNM. • 1 ♂; Yamagata, Iide, Mt. Iide; 37.85122°N, 139.78064°E, alt. 600 m; 23 May. 2015; Y. Imada leg.; CYI. • 2 ♂, 1 ♀; Yamagata, Oguni, Nukumidaira; 37.92293°N, 139.67546°E; alt. 433 m; larvae collected: 9 Nov. 2014, emerged: 22 Apr. 2014; Y. Imada leg.; CYI. **Russia** • Primorsky Krai, Khasansky District, Primorsky Settlement, Zolotistyy [Golden] Stream; 43.10075°N, 131.54862°E; alt. 62 m; 10 Jun. 2007 – 11 Jun. 2007; N.M. Paramonov leg.; CKLP. • 2 ♂; Sakhalin Oblast, Yuzhno-Kurilsk Urban Settlement, Kuril/Kunashir Island, near Lagunnoe Lake; 44.0623°N, 145.759°E; alt. 20 m; 11 Jul. 1954 – 12 Jul. 1954; leg. N.A. Violovich ZIN. • 1 ♂; Sakhalin Oblast, Kunashir Island, Mendeleevo Settlement; 43.971°N, 145.694°E; alt. 220 m; 28 Jun. 1973; I.M. Kerzhner leg.; ZIN. • 1 ♀; Sakhalin Oblast, Kunashir Island, Alekhino Settlement [uninhabited]; 43.91°N, 145.52°E; alt. 5 m, 29 Jun. 1962; G.O. Krivoluckaja leg.; ZIN. • 2 ♂; Sakhalin Oblast, Kunashir Island, the mouth of the Tjatina River; 44.2711°N, 146.1583°E; alt. 15 m; 21 Jul. 2014; Y.N. Sundukov leg.; ZIN.

##### Redescription.

**Head.** Dark brown to black, with greyish pubescence (Fig. 23B–D). Rostrum short without nasus. Mouth parts pale brown to brown. Palpus brown to dark brown, five segmented; last segment 1.2–1.4 × longer than penultimate (Fig. 23B, D). Scape cylindrical, 1.6–1.8 × longer than pedicel; pedicel ovate; pedicel brown, scape yellow to brown (Figs 4D, 23B, D); flagellum 14-segmented, pale brown to brown, monochrome or gradually darkening from base to tip. Flagellar segments simple, cylindrical in both sexes, not expanded ventrally; all male flagellomeres and 2–8 female flagellomeres covered with sparse whitish setae/sensilla; sensilla slightly denser in ventral side; verticels less prominent, 4–6 verticels not showing clear arrangement (Figs 4D, 23B, D).

**Thorax.** General colour shiny dark brown to black, with yellowish area in lateral side. Pronotum dark brown to black. Anterior part of mesonotum brown with black stripes or patches, usually forming three longitudinal, black markings on presutural area of scutum, and two drop-shaped black markings on postsutural area of scutum (Fig. 23C) or one large marking; black parts bare and shiny; paler parts with pubescence and with several long yellow hairs, forming longitudinal lines (Fig. 23C). Dorsal pleural area, base of wing, anepimeron, and base of halter yellowish. Coxa black, ventrally paler (Fig. 23B); trochanter yellow; femur gradually darkening distally, basal part yellowish, apical part dark brown to black; tibia and tarsus dark brown. Wing hyaline; veins brown; pterostigma pale; three branches of M reaching wing margin, M_1_ at same level as M_1+2_, cell a_2_ narrow, > 8 × longer than wide (Fig. 5D); membrane with interference patterns, visible with dark background (Fig. 23A). Halter stem pale brown, knob brown.

**Abdomen.** Black, without any distinct patterns (Fig. 23A).

**Male terminalia**: Relatively small, uniformly black, directed caudally (Fig. 23A). Tergite 9 fused with gonocoxite and sternite 9 (Fig. 24C); tergite 9 with median lobe, with notch at middle (Fig. 24A); lateral lobes of tergite 9 not prominent. Sternite 9 reduced to narrow band (Fig. 24B, C). Gonocoxite relatively large, 1.2–1.4 × longer than tergite 9, in lateral view (Fig. 24C); without any distinct lobes (Fig. 24B, C); inner side of gonocoxite membranous; small round sclerotised patch on membranous area between gonocoxites present (Fig. 24B), triangular in lateral view (Fig. 24C, F); holding base of aedeagal complex if it moved dorsally. Gonostylus with a strongly sclerotised, claw-like outgrowth; tip of gonostylus finger-like (Fig. 24A, C). Aedeagus complex as long as gonocoxite and sternite 9 together; sperm pump and ejaculatory apodeme, partly covered by parameres (Fig. 24F); interbase simple L-shaped, both in lateral and dorsal/ventral views (Fig. 24D–F); posterior part blade-like, with a small notch on dorsal side, in lateral view (Fig. 24F); aedeagus widened and curved dorsally at right angle in midlength, covered with prominent spines on ventral and lateral sides; membranous area on ventral side behind ventral spines, make flexible the aedeagus and able to straighten, probably during copulation (Fig. 24D, F); aedeagus with apical branches short, directed caudally; median branch slightly longer and wider than lateral ones (Fig. 24D, F).

**Female terminalia**: Black, tips of cercus and hypopygial valve yellowish (Fig. 23E). Tergite 8, 2 × larger than tergite 9 in lateral view (Fig. 25B); not divided at middle (Fig. 25A). Caudal margin of tergite 9 straight in lateral view (Fig. 25B). Lateral lobe of tergite 10 finger-like, 3 × longer than wide, well separated from tergite 10 (Fig. 25A); triangular sclerite large, separated from tergite 10 (Fig. 25A). Cercus and hypogynial valve blade-like, relative narrow compared to other cylindrotomines (Fig. 25B); rough surface on dorsal tip of cercus hardly recognisable, only a few small pyramid teeth present. Genital fork large, heavily sclerotised plate; common sperm duct after genital opening relatively short, hardly recognisable; sperm ducts carrot-shaped; wall of sperm wrinkled (Fig. 25C); three round spermathecae present, diameter ~ 1/3–1/2 × width of genital fork (Fig. 25D).

##### Distribution.

South Korea, Japan (Honshu I and Shikoku I), and Russia (Jewish Autonomous Oblast, Sakhalin Oblast (Kuril Is: Kunashir I) (Oosterbroek 2021). First records from Japan: Kyushu I (Fig. 22B).

##### Comments.

As with other Cylindrotominae species that have simple antennae and three M vein branches, this species was also originally described as *Phalacrocera* (Alexander 1919). Later Takahashi (1960) moved this species to the *Liogma* genus based on the morphological similarity of the immature stages with *Liogmanodicornis* (Osten Sacken, 1865). However, *L.mikado* is a morphologically and genetically quite distinct species from the other *Liogma* species, and the exact phylogenetic position remains unclear.

#### ﻿ Liogma nodicornis

Taxon classificationAnimaliaDipteraCylindrotomidae

(Osten Sacken, 1865)

A7A38AA5-ED61-5E5F-A1E0-C9B520E5CD86

Figs 26 , 27

Liogma flaveola Alexander, 1919.

##### Non-type material examined.

**Canada** • 1 ♀; Manitoba, Winnipeg, Birds Hill Park, cedar bog; 50.03°N, 96.91° W; alt. 250 m; 20 Jun. 2003; F. Brodo leg.; CKLP. • 1 ♂, 2 ♀; Ontario, Ottawa, Stony Swamp; 45.3°N, 75.83° W; alt. 115 m; 7 Jun. 2011; • 1 ♂; same locality; 30 May. 2011; F. Brodo leg.; CKLP. **Usa** • 1 ♂; New Hampshire, Twin mountains, vochtig loofbos; 44.218°N, 71.415° W; alt. 600 m; 20 Jun. 1982; P. Oosterbroek / I. Tangelder leg.; CKLP. • 1 ♂; • 1 ♂; Michigan, Delta Co., 11 Jun. 1860; R. and K. Dreisbach leg; « Green label under the geographical label: *Liogmanodicornis* (O.S.). NOTE genotype of *Liogma* ».

##### Supplementary description.

**Figure 26.**
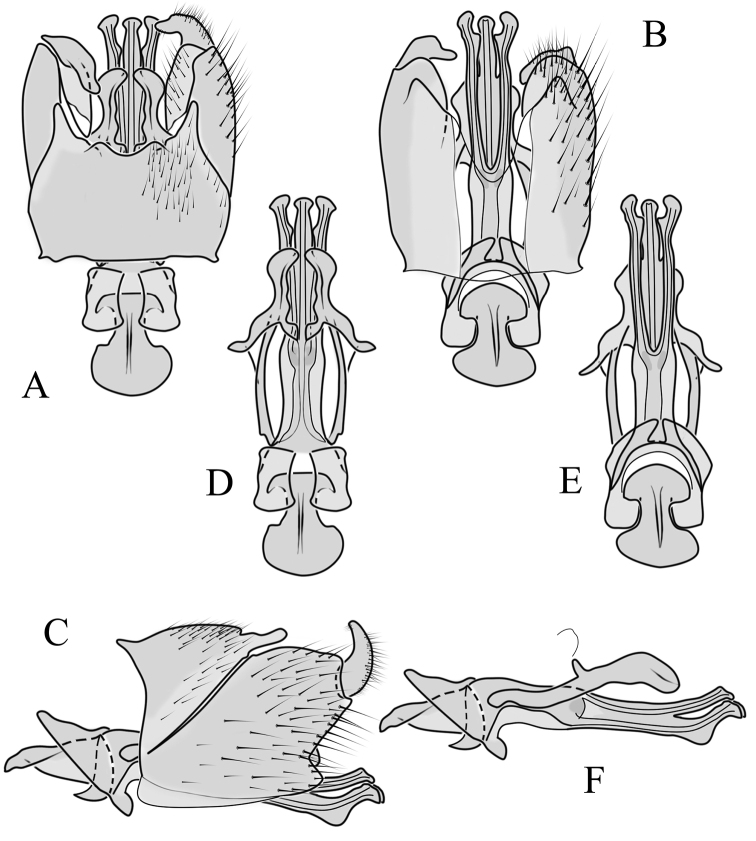
Male genital structures of *Liogmanodicornis* (Osten Sacken, 1865) **A** terminalia, dorsal view **B** terminalia, ventral view **C** terminalia, lateral view **D** aedeagus complex, dorsal view **E** aedeagus complex, ventral view **F** aedeagus complex, lateral view.

**Male terminalia** directed caudally. Tergite 9 fused with gonocoxite at base (Fig. 26C); caudal margin of tergite 9 with prominent lateral lobe, finger-like in lateral view (Fig. 26C), elongated, triangular in dorsal view (Fig. 26A); posterior margin with additional small, triangular outgrowth (Fig. 26A). Sternite 9 membranous (Fig. 26B). Gonocoxite 1.3–1.5 × longer than tergite 9 (including lobe); ventral lobe relatively small, triangular both lateral and ventral views, covered by few setae (Fig. 26B, C); inner surface of gonocoxite with hairs, proximal corner with hairless, paler area (Fig. 26A). Gonostylus simple, tapering to distal end. Aedeagus complex very large, 1.8–1.9 × longer than gonocoxite, in lateral view (Fig. 26C); ejaculator apodeme large, not covered by paramere in lateral view (Fig. 26F); tip of interbase directed inward in dorsal view (Fig. 26D), and ventrally in lateral view (Fig. 26F); dorsal lobe between interbases small, membranous, hardly noticeable (Fig. 26F); aedeagus long, trifid, median branch slightly shorter (Fig. 26D–F); lateral branch prolonged ventrally/caudally (Fig. 26F); tips of branch flattened; sperm ducts branching from elongated portion of sperm pump, branching area dark (Fig. 26F).

**Figure 27.**
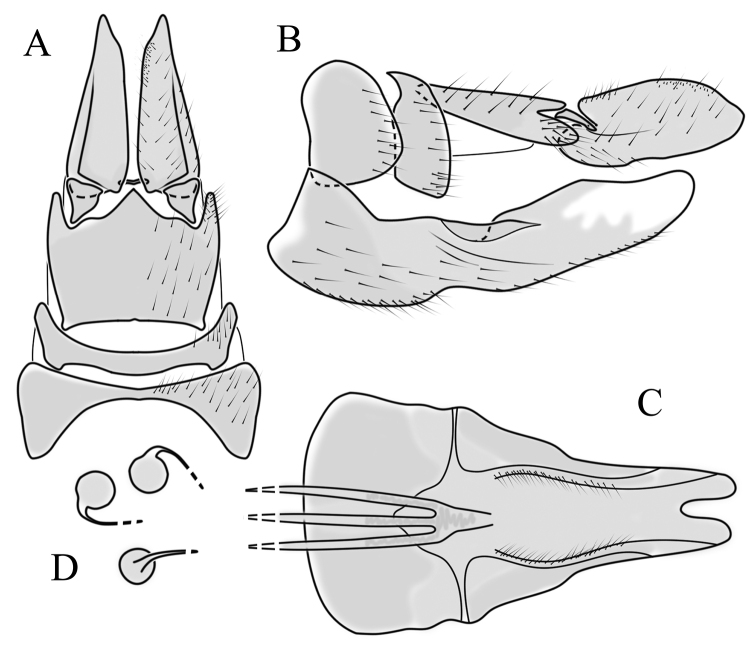
Female genital structures of *Liogmanodicornis* (Osten Sacken, 1865) **A** terminalia, dorsal view **B** terminalia, lateral view **C** sternite 8, hypogynial valve, and sperm ducts, inner dorsal view **D** spermathecae.

**Female terminalia**: Tergite 8, ~ 1.7–1.8 × wider than tergite 9 in lateral view (Fig. 27B), not divided medially (Fig. 27A). Triangular sclerite ~ 1/4 of length of tergite 10; sclerite separated from tip of tergite 10; lateral lobe of tergite 10 medium sized, 2 × as long as wide, with few long hairs (Fig. 27B). Cercus wide, with blunt notches on dorsal, close to tip and ~ 1/3 of mid-length on ventral margin. Hypogynial valve relative long, dorsal margin close to tip concave (Fig. 27B). Common spermathecal duct recognisable; sperm ducts simple, narrow tubes, (Fig. 27C); three round spermathecae with curved, suddenly tapering ducts (Fig. 27D).

**Distribution**: Canada and USA from (Alberta to Newfoundland, south to South Dakota, South Carolina and Georgia) (Oosterbroek 2021).

#### ﻿ Liogma serraticornis

Taxon classificationAnimaliaDipteraCylindrotomidae

Alexander, 1919

1FB4E9F5-D070-51A7-B585-28C246A5037F

**Figure 28.**
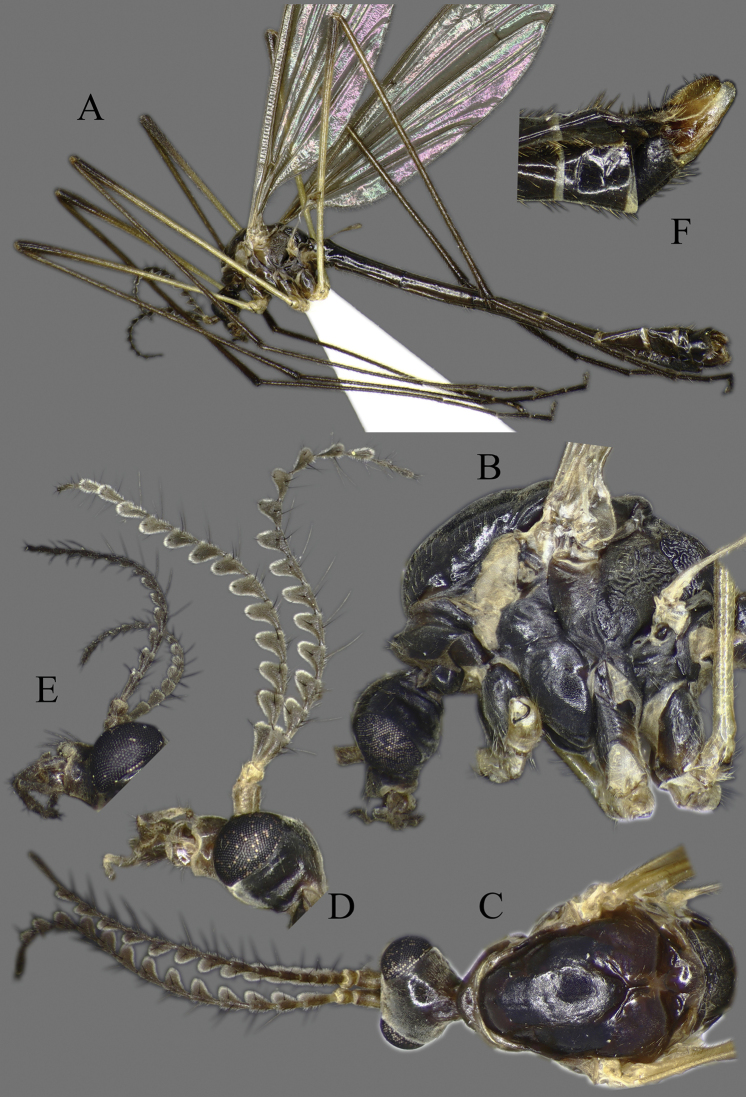
*Liogmaserraticornis* Alexander, 1919 **A** habitus of male, lateral view (colouration of wings is artefact) **B** head and thorax, lateral view **C** head and thorax of male, dorsal view **D** head of male, lateral view **E** head of female, lateral view **F** female terminalia lateral view.

**Figure 29.**
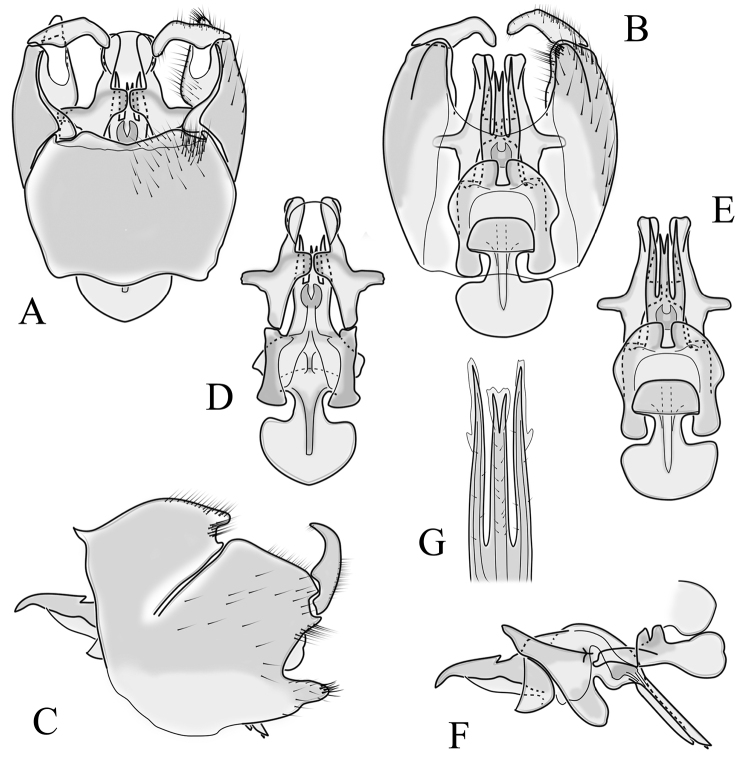
Male genital structures of *Liogmaserraticornis* Alexander, 1919 **A** terminalia, dorsal view **B** Terminalia, ventral view **C** terminalia, lateral view **D** aedeagus complex, dorsal view **E** aedeagus complex, ventral view **F** aedeagus complex, lateral view **G** tip of the aedeagus.

**Figure 30.**
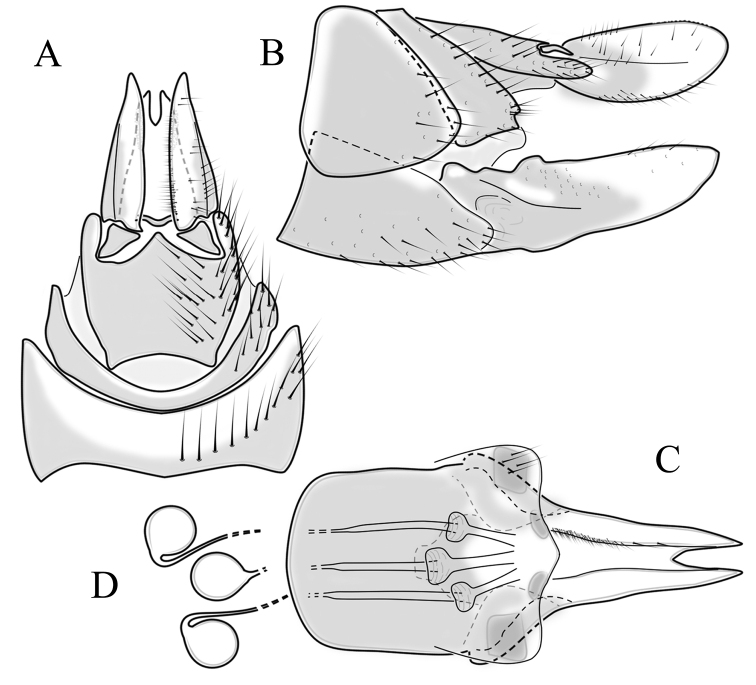
Female genital structures of *Liogmaserraticornis* Alexander, 1919 **A** terminalia, dorsal view **B** terminalia, lateral view **C** sternite 8, hypogynial valve, genital fork, and sperm ducts, inner dorsal view **D** spermathecae.

**Figure 31.**
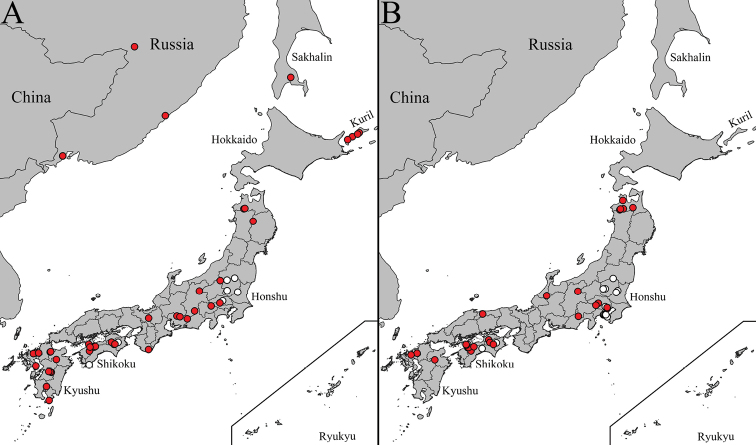
Occurrence data in Japan and surrounding areas of **A***Liogmaserraticornis* Alexander, 1919 **B***Triogmakuwanai* (Alexander, 1913). Red dots indicate locations of investigated specimens, white dots indicate approximate locations of literature data.

Figs 4F , 5F , 28 , 29 , 30 , 31A

Liogma serraticornis in Alexander 1919: 345–346: original description; Alexander 1928: 11: distribution, illustration.; Alexander 1949: 195 comparison.; Esaki 1950: illustration.; Alexander 1953b: 77: faunistic record, distribution.; Ishida 1955: 75–76: distribution.; Takahashi 1960: 84: distribution.; Sidorenko 1999: 68–70: identification key, illustration, distribution.; Nakamura 2001: 23–29: identification key, illustration, distribution, faunistic records.; Paramonov 2004b: 69: faunistic record.; Paramonov 2006: 888–889: identification key, distribution.; Nakamura 2014: 54: distribution.; Kato and Suzuki 2017: 16: distribution.; Imada 2020: biology and ecology of larvae.
Liogma fuscipennis in Alexander 1932 111–112: original description; Alexander 1953a: 55–56, syn. nov.

##### Type material examined.

*Liogmaserraticornis* Alexander: ***Paratype***: **Japan** • ♂; Saitama, 29 May. 1919; R. Takahashi leg.; USNM.

##### Non-type material examined.

**Japan** • 1 ♂; Aichi, Seto, Iwaya-cho, near Iwayada Park; 35.23957°N, 137.15084°E; alt. 300 m; 4 May. 2016; D. Kato leg.; BLKU. • 1 ♂; Aichi, Toei, Futto; 35.10117°N, 137.6607°E; alt. 390 m; larva collected: 9 Mar. 2014, emerged: 1 Apr. 2014; M. Kato leg.; CYI. • 1 ♀; Aichi, Toyota, Kawashimo, triburary of Yahagi River; 35.20376°N, 137.30125°E; alt. 140 m; 4 May. 2014; D. Kato leg.; BLKU. • 1 ♀; Aomori, Hirosaki, Ichinowatari-washinosu; 40.51923°N, 140.43889°E; alt. 205 m; 7 Jun. 2013; D. Kato leg.; BLKU. • 1 ♀; Aomori, Hirosaki, Koguriyama, Inekari River; 40.53658°N, 140.48701°E; alt. 170 m; 7 Jun. 2013; D. Kato leg.; BLKU. • 1 ♀; Ehime, Matsuyama, forest seep and stream; 33.86152°N, 132.82591°E; alt. 180 m; 20 Apr. 2019; L.-P. Kolcsár leg.; CKLP. • 1 ♂; Ehime, Odamiyama; 33.53°N, 132.86°E; 26 May. 1963; M. Miyatake leg.; EUMJ. • 5 ♂; Ehime, Saijo, spring and mosses rocks; 33.75504°N, 133.15377°E; alt. 1480 m; 16 Jun. 2019; L.-P. Kolcsár leg.; CKLP. • 1 ♂, 1 ♀; Ehime, Toon-shi, Saragamine; 33.72361°N, 132.88602°E; alt. 955 m; 21 May. 2017; K. Kuroda leg.; EUMJ. • 1 ♂, 1 ♀; Ehime, Wakayama, Mount Ishizuchi; 33.76491°N, 133.12948°E; alt. 1600 m; 5 Jun. 2019; L.-P. Kolcsár leg.; CKLP. • 2 ♀; Ehime, Wakayama, River Omogo gorge; 33.72581°N, 133.10291°E; alt. 750 m; 5 Jun. 2019; L.-P. Kolcsár leg.; CKLP. . • 1 ♀; Ehime, Kumakogen, headwaters, stream; 33.56476°N, 132.93501°E; alt. 1387 m; 17 Jun. 2019; L.-P. Kolcsár leg.; CKLP. • 1 ♀; Fukuoka, Miyako, small stream and Japanese cedar forest; 33.49796°N, 130.95861°E; alt. 686 m; 21 May. 2019; L.-P. Kolcsár leg.; CKLP. • 1 ♀; Fukuoka, Soeda, rocky streem and moss covered cliff; 33.48309°N, 130.93289°E; alt. 900 m; 21 May. 2019; L.-P. Kolcsár leg.; CKLP. • 1 ♂; Fukushima, Hinoemata, Ozebunanomori Museum; 36.99082°N, 139.27803°E; alt. 1230 m; 28 Jun. 2015; M. Kato leg.; CYI. • 1 ♂, 1 ♀; Iwate, Hachimantai, Matsuoyoriki; 39.89958°N, 140.89155°E; alt. 1200 m; larvae collected: 14 Jun. 2014, emerged: 4 Jul. 2014; Y. Imada leg.; CYI. • 1 ♂; Kagoshima, Inaodake; 31.12°N, 130.88°E; 11 May. 1952; Ito-Issiki leg.; USNM. • 1 ♀; Kagoshima, Kirishima, around Amori-gawa River, Hayato-cho-Kareigawa; 31.79821°N, 130.75275°E; 80 m; 28 Apr. 2018; D. Kato leg.; BLKU. • 1 ♂; Kumamoto, Gokanosho; 32.53°N, 130.86°E; 5 May. 1926; S. Issiki leg.; USNM. • 1 ♀; Kumamoto, Yatsushiro, Izumimachi-Momiki; 32.4915°N, 130.99084°E; alt. 1060 m; 11 May. 2016; T. Hosoya, S. Kakizoe leg.; BLKU. • 1 ♀; Kumamoto, Yatsushiro, Momiki-gawa river, Izumimachi-Momiki and Hagi; 32.51417°N, 130.93927°E; alt. 530 m; 11 May. 2016; D. Kato leg.; BLKU. • 1 ♂; Kyoto, Kyoto, Kibune; 35.13681°N, 135.76622°E; alt. 458 m; 1 May. 2016; Y. Imada leg.; CYI. • 1 ♂; Nagano, Oshika, Oike; 35.4887°N, 138.0219°E; alt. 1250 m; 19 Oct. 2015; Y. Imada leg.; CYI. • 1 ♂; Nagano, Ueda, Sanada-machi, Irikaruizawa; 36.47441°N, 138.25481°E; alt. 777 m; 16 May. 2012; D. Kato leg.; BLKU. • 1 ♂; Nagasaki, Unzen; 32.8°N, 130.23°E; May 1926; E. Svenson leg.; USNM. • 1 ♂; Oita, Kokonoe, Tano; 33.11621°N, 131.23541°E; alt. 1150 m; 7 May. 2016; D. Kato leg.; BLKU. • 2 ♂; Saga, Kanzaki, springs; 33.43401°N, 130.36866°E; alt. 980 m; 23 May. 2019; L.-P. Kolcsár leg.; CKLP. • 1 ♂; Saga, Karatsu, Tsubakiyama Pond, Hamatama-machi-torisu; 33.40414°N, 130.1064°E; alt. 630 m; 26. Apr. 2015; D. Kato leg.; BLKU. • 1 ♂, 1 ♀; Saga, Saga, Kase river near Hokuzan Dam, Fujimachi-sekiya; 33.43322°N, 130.23212°E; alt. 325 m; 23 Apr. 2015; D. Kato leg.; BLKU. • 1 ♂; Tokushima, Miyoshi, around Matsuogawa Dam, Higashiiya-Ochiai; 33.96478°N, 133.93908°E; alt. 900 m; 15 May. 2015; D. Kato leg.; BLKU. • 2 ♂; Tokushima, Minokosi, Mt. Tsurugi; 33.87°N, 134.11°E; alt. 1400 m; 1 Jun. 1950; Issiki-Ito leg.; EUMJ. • 1 ♂; Wakayama, Kozagawa, Takinohai; 33.6058°N, 135.76127°E; alt. 80 m; 13 Apr. 2014; M. Kato leg.; CYI. • 1 ♀; Yamanashi, Koshu, Enzankamihagihara, Kaminichikawa Pass; 35.73161°N, 138.83208°E; alt. 1580 m; 8 Jul. 2014; D. Kato leg.; BLKU. **Russia** • 1 ♂; Khabarovsk Krai, Khabarovsk City; 48.48022°N, 135.07191°E; alt. 80 m; 2 Jun. 2014 – 6 Jun. 2014; N.E. Vikhrev leg.; ZIN. • 1 ♂, 1 ♀; Primorsky Krai, Khasansky District, Primorsky Settlement, Zolotistyy [Golden] Stream; 43.10075°N, 131.54862°E; alt. 62 m; 13 Jun. 2007; N.M. Paramonov leg.; CKLP. • 1 ♂; Primorsky Krai, Kedrovaya Pad Nature Reserve; 43.10075°N, 131.54862°E; alt. 62 m; 7 Jul. 1940; A.S. Monchadskij leg. • 1 ♂; same locality; 12 Jun. 1962; E.P. Narchuk leg.; • 1 ♂; same locality; 2 Jul. 1962; E.P. Narchuk leg.; ZIN. • 1 ♂; Primorsky Krai, Kedrovaya Pad Nature Reserve, bog near Kedrovka River; 43.10075°N, 131.54862° E; alt. 62 m; 16 Jun. 2007; • 1 ♂; same locality; 1 Jun. 2007 – 11 Jun. 2007; N.M. Paramonov leg.; ZIN. • Primorsky Krai, Kedrovaya Pad Nature Reserve, Zolotistyy [Golden] Stream; 43.1007°N, 131.5486°E; alt. 62 m; 2007.06.13, 1 ♂, N.M. Paramonov leg.; ZIN. • 1 ♂; Primorsky Krai, Terney District, Terney Urban-type Settlement, Lower Serebryanka [Sanhobe] River Valley; 45.09314°N, 136.5852°E; alt. 60 m; 18 Jun. 1937; K.J. Grunin leg.; ZIN. • 5 ♂; Sakhalin Oblast, Yuzhno-Kurilsk Urban Settlement, Kuril Islands, Kunashir Island, near Lagunnoe Lake; 44.062°N, 145.759°E; alt. 20 m; 11 Jul. 1954; N.A. Violovich leg.; ZIN. • 2 ♂; Sakhalin Oblast, Kunashir Island, lower course of the Saratovskaja River; 44.26042°N, 146.09912°E; alt. 16 m; 3 Jul. 2014 – 6 Jul. 2014; Y.N. Sundukov leg.; ZIN. • 1 ♂; Sakhalin Oblast, Kunashir Island, lower course of the Filatova River; 44.19078°N, 146.02006°E; alt. 60 m; 27 Jun. 2013 – 28 Jun. 2013; Y.N. Sundukov leg.; ZIN. • 1 ♂; Sakhalin Oblast, Kunashir Island, Alekhino Settlement [uninhabited]; 43.918°N, 145.529°E; alt. 5 m; 29 Jun. 1962; G.O. Krivoluckaja leg.; ZIN. • 1 ♀; Sakhalin Oblast, Sakhalin Island, Yuzhno-Sakhalinsk City; 46.959°N, 142.738°E; alt. 50 m; 22 Jun. 1956; • 1 ♂, 1 ♀; same locality; 27 Jun. 1956; N.A. Violovich leg.; ZIN.

##### Redescription.

**Head.** Black with weak greyish pubescence (Fig. 28C, D). Rostrum short without nasus, but with few hairs (Fig. 28B, E); rostrum and mouthparts brown to black (Fig. 28B, E). Palpus brown to black, five-segmented; first two segments sometimes darker than the rest; last segment 1.3–1.8 × longer than penultimate. Scape cylindrical 1.5–2 × longer than pedicel (Fig. 4F); pedicel ovate; pedicel and scape same coloured or scape slightly darker, yellowish brown to brown; flagellum 14 segmented, monochrome dark brown to black; flagellar segments greatly expanded ventrally in male, last flagellomere cylindrical (Figs 4F, 28D). Flagellomeres 2–6 or 7 extended in female, remaining segments cylindrical (Figs 4F, 28E). Extended flagellomeres covered with dense whitish sensilla; 2–4 long verticels on dorsal surface, two verticels in lateral surface, two shorter on ventral side; first flagellomere always bearing additional verticels (Fig. 4F).

**Thorax**. Uniformly black with very weak greyish pubescence (Fig. 28B, C). Pleural area, base of wing, and base of halter yellowish or greyish white (Fig. 28B). Scatter, pale, short hairs present on mesonotum, forming two barely visible lines. Ventral part of thorax generally dark brown to uniformly black. Anterior half or more of mediotergite and almost all pleurotergite rugose (Fig. 28B). Trochanter yellow to pale brown; femur gradually darkening, basal part yellowish, apically dark brown; tibia gradually darkening distally, pale brown to dark brownish black; tarsus uniformly black (Fig. 28A). Wing hyaline, tinged with yellowish brown (typical “*serraticornis*” form) or infuscated (“*fuscipennis*” form); pterostigma pale brown to black; veins dark brown; three branches of M reaching wing margin; M_1_ at same level as M_1+2_, cell a_2_ less than 6 × longer than wide (Fig. 5F); membrane with interference patterns, visible with dark background (Fig. 28A). Halter monochrome, yellowish brown to black (Fig. 28A).

**Abdomen.** Black, without any clear patterns (Fig. 28A).

**Male terminalia**: Relatively small, uniformly black or ventral parts of gonocoxite paler; directed caudally (Fig. 28A). Tergite 9 fused with gonocoxite (Fig. 29C); proximal margin with two obtuse triangular lobes, which bent back under tergite 9 (Fig. 29A). Sternite 9 fully membranous (Fig. 29B). Gonocoxite large, 1.7–1.8 × longer than tergite 9; with long ventral lobe, tip covered by pale, short spine-like setae (Fig. 29B, C); inner surface of gonocoxite sclerotised, forming dorsal plate with conspicuous edge next to tergite 9 (Fig. 29A). Gonostylus simple, tapering to tip (Fig. 29A–C). Aedeagus complex large, 1.2–1.3 × longer than gonocoxite. Ejaculator apodeme and sperm pump large, together half of length of aedeagal complex (Fig. 29D–F); not covered by parameres (Fig. 29F); interbase spoon-like with small notch apically in lateral view (Fig. 29F); dorsal lobe of interbase small, directed inward in dorsal view (Fig. 29D); dorsal lobe between interbases large, globular and semi-transparent, as wide as tip of interbase in lateral view (Fig. 29F). Aedeagus straight, directed ventrally in 45°; sperm ducts branching from elongated portion of sperm pump, base of branches darkened (Fig. 29D, F); middle branch of aedeagus shorter than lateral branches; each with small spines ventrally; apical end of branches with hyaline membranous tissue (Fig. 29G).

**Female terminalia**: Brown to black, end of cercus and hypogynial valve yellowish (Fig. 28F). Tergite 8 > 2 × wider than tergite 9 in lateral view (Fig. 30B); not divided medially in dorsal view (Fig. 30A). Tergite 9 widening ventrally in lateral view, with small notch at posterior corner (Fig. 30B). Tergite 10 with triangular sclerite smaller, ~ 1/3 of length of tergite 10; sclerite separated from tergite 10 (Fig. 30B); lateral lobe relatively long, at least 2 × longer than wide (Fig. 30B). Cercus oval; hypogynial valve elongated, blade-shaped. Cercus on dorsal surface close to apical end weakly, but clearly rugose, serrate (Fig. 30B); ventral margin of cercus without notch, evenly curved (Fig. 30B). Common spermathecal duct, short, indistinct; spermathecal ducts with extended parts, golf-tees-like (Fig. 30C); three round, spermathecae present, duct curved or straight (Fig. 30D).

##### Distribution.

Japan (Hokkaido I, Honshu I, Shikoku I, and Kyushu I) and Russia (Primorsky Krai, Sakhalin Oblast (incl. Kuril I) (Oosterbroek 2021). First record from Khabarovsk Krai, Russia (Fig. 31A).

##### Comments.

The morphological comparison of this species with *L.brevipecten* is discussed under that species. Colouration is variable within specimens of *Liogmaserraticornis*. Usually, colouration is black with a paler pleural area, and the wing membrane is almost transparent, tinged with pale yellowish brown. In darker specimens, the pleural area and wing membrane is infuscated. This darker form was described as a separate species, *Liogmafuscipennis* Alexander, 1932, but was later synonymised with *L.serraticornis* (Alexander 1953). No genital and genetic difference between the paler and darker specimens were found during our study.

#### ﻿ Phalacrocera replicata

Taxon classificationAnimaliaDipteraCylindrotomidae

(Linnaeus, 1758)

E32EFAB8-B7EF-53DC-B9E9-327B748DAA80

Figs 32 , 33

Phalacrocera nudicornis (Schummel, 1829)
Phalacrocera brevirostris (Zetterstedt, 1838)
Phalacrocera diversa (Walker, 1856)
Phalacrocera neoxena Alexander, 1914.

##### Non-type material examined.

**Canada** • 2 ♂, 1 ♀; Ontario, Ottawa, Stony Swamp; 45.3°N, 75.83° W; alt. 115 m; 10 May. 2017; F. Brodo leg.; CKLP. **Finland** • 2 ♂, 1 ♀; Kirkkonummi, Stormossen. 60.07901°N, 24.57980°E; alt. 7 m; 2 Jun. 2016; E. Viitanen leg.; CKLP. • 1 ♂, 2 ♀; Kaarina, Jarvela; 60.46157°N, 22.37418°E; alt. 38 m; 18 May. 2016 – 1 Jun. 2016; E. Viitanen leg.; Malaise trap; CKLP. • 1 ♂; Tervola, Karhakkamaanjanka; 66.19764°N, 25.12660°E; alt. 58 m; 25 May. 2004 – 28 Jun. 2004; J. Salmela leg.; CKLP. **Russia** • 1 ♂; Krasnoyarsk Krai, Turukhansky District, Igarka City, within the settlement, swampy lake shore in the city; 67.466°N, 86.581°E; alt. 23 m; 30 Jun. 1967; K.B. Gorodkov leg.; CKLP. • 1 ♀; Krasnoyarsk Krai, Igarka City, within the settlement, sedge swamp; 67.466°N, 86.581°E; alt. 23 m; 1 Jul. 1967; K.B. Gorodkov leg.; CKLP. **Usa** • 2 ♂; Michigan, Cheboygan, hard wood swamp; 45.29277°N, 84.42805° W, alt. 280 m; 20 May. 2015; F. Brodo leg.; CKLP.

##### Supplementary description.

**Figure 32.**
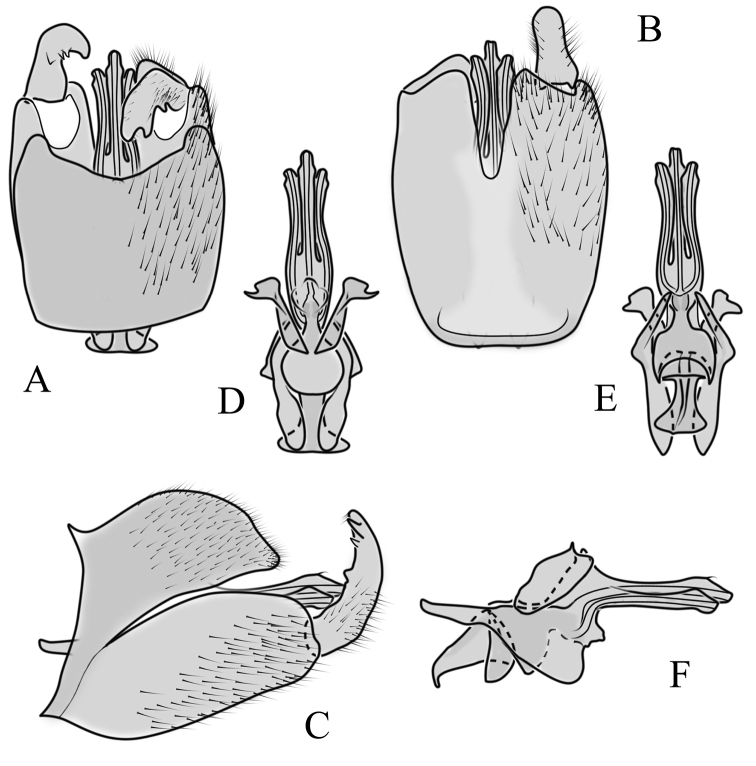
Male genital structures of *Phalacrocerareplicata* (Linnaeus, 1758) **A** terminalia, dorsal view **B** terminalia, ventral view; **C** Terminalia, lateral view **D** aedeagus complex, dorsal view **E** aedeagus complex, ventral view **F** aedeagus complex, lateral view.

**Male terminalia** directed dorsally. Tergite 9 fused with gonocoxite and sternite 9 (Fig. 32C); caudal margin of tergite 9 with small, rounded lateral lobe both ventral and lateral view (Fig. 32A, C); posterior margin U- or V-shaped. Sternite 9 reduced to narrow band (Fig. 32B, C). Gonocoxite 1.2–1.3 × longer than tergite 9; ventral lobe relatively small, rounded (in dry specimen looks more finger-like) (Fig. 32B); apical lobe indistinct; inner surface of gonocoxite sclerotised, with few hairs, without evident modifications. Gonostylus comparable large and complex, with a subapical tooth on outer margin; additional two or three smaller teeth basally (Fig. 32A, C). Aedeagus complex 1.2–1.3 × longer than gonocoxite in lateral view (Fig. 32C); ejaculator apodeme large, not covered by paramere in lateral view (Fig. 32F); interbase seems flat and wide, with a small dorsal tooth in lateral view (Fig. 32F); interbase directed dorso-laterally, with a deep notch on tip in dorsal view (Fig. 32D, E); dorsal lobe between interbases indistinct or absent (Fig. 32D); aedeagus half as long as entry aedeagus complex; aedeagus trifid, straight; median branch longer than lateral branches (Fig. 32D–F), with a triangular dorsal outgrowth; tip slightly bifid or trifid, depend on angle (Fig. 32A); lateral branches slightly curved laterally in dorsal and ventral view (Fig. 32D, E); tips of branch widened dorsally; sperm ducts branching from short elongation of sperm pump; branching area dark (Fig. 32D–F).

**Figure 33.**
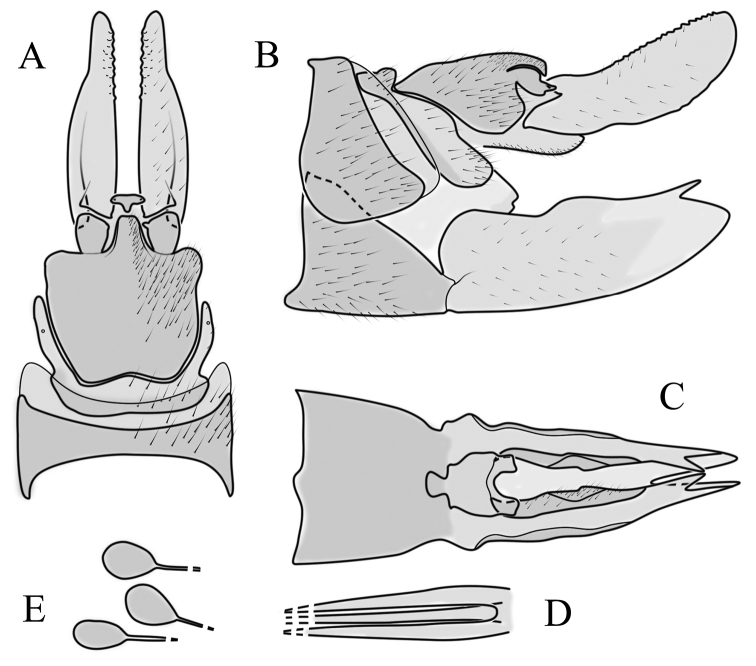
Female genital structures of *Phalacrocerareplicata* (Linnaeus, 1758) **A** terminalia, dorsal view **B** terminalia, lateral view **C** sternite 8, hypogynial valve, and genital fork, inner dorsal view **D** sperm ducts **E** spermathecae.

**Female terminalia**: Tergite 8 posterior part membranous, with few hairs, but not divided medially (Fig. 33A); wider than tergite 9 in lateral view (Fig. 33B). Tergite 10 with small, slightly separated median lobe in middle of posterior margin (Fig. 33A, B); covered with short hairs. Triangular sclerite widely fused with tip of tergite 10; tergite 10 without lateral lobe (Fig. 33B). Cercus elongated blade, with dorsal margin with weakly serrate margin (Fig. 33B). Hypogynial valve wide and long, dorsal margin close to tip with tooth-like lobe, directed caudally (Fig. 33B, C). Common spermathecal duct short or indistinct; sperm ducts simple, without distinct pattern, tapering proximally (Fig. 33D); three spermathecae elongated, with straight duct (Fig. 33E).

##### Distribution.

Widely distributed in Holarctic. It has been reported from the Nearctic: Canada and USA from Ontario and Quebec, south to Michigan, Pennsylvania and Massachusetts. Palearctic: Austria, Belarus, Belgium, China (Heilongjiang), Czech Republic, Denmark, Estonia, Finland, France, Germany, Great Britain, Ireland, Italy (north), Lithuania, Mongolia, Netherlands, Norway, Poland, Russia: North European Russia, Central European Russia, East Siberia (Irkutsk Oblast), Far East (Republic of Sakha (Yakutia), Spain (Zamoro), Sweden, Switzerland, Ukraine (Oosterbroek 2021, Paramonov and Pilipenko 2021).. Here we record the species for the first time from Krasnoyarsk Krai, East Siberia, Russia.

#### ﻿ Phalacrocera tipulina

Taxon classificationAnimaliaDipteraCylindrotomidae

Osten Sacken, 1865

6BEB9DF8-91D2-5EA5-9131-2A993D5110EA

Figs 34 , 35

##### Non-type material examined.

**Canada** • 1 ♂; Quebec, Schefferville, Lac Le Jeune; 54.83006°N, 66.82436° W; alt. 500 m; 13 Jul 1981; F. Brodo leg.; CKLP. • 1 ♂; Quebec, Schefferville, Ashtray lake, 26 km SE from Schefferville; 54.66656°N, 66.65095° W; alt. 500 m; 15 Jul. 1981; F. Brodo leg.; CKLP. • 1 ♂; Quebec, Schefferville, Iron Arm, 18 km SE from Schefferville; 54.70211°N, 66.7630° W; alt. 530 m; 18 Jul. 1981; F. Brodo leg.; CKLP. **Usa** • 3 ♂, 2 ♀; Maine, Jonesport, Rogue Island, Bonney Point fen near coast; 44.57845°N, 67.52928° W; alt. 20 m; 2 Jun. 2011; F. Brodo leg.; CKLP. • 1 ♂, 1 ♀; Virginia, Pearisburg, Mountain Lake; 37.36106°N, 80.53231° W; alt. 1190 m; 25 Feb. 2018; Y. Imada leg.; CKLP.

##### Supplementary description.

**Figure 34.**
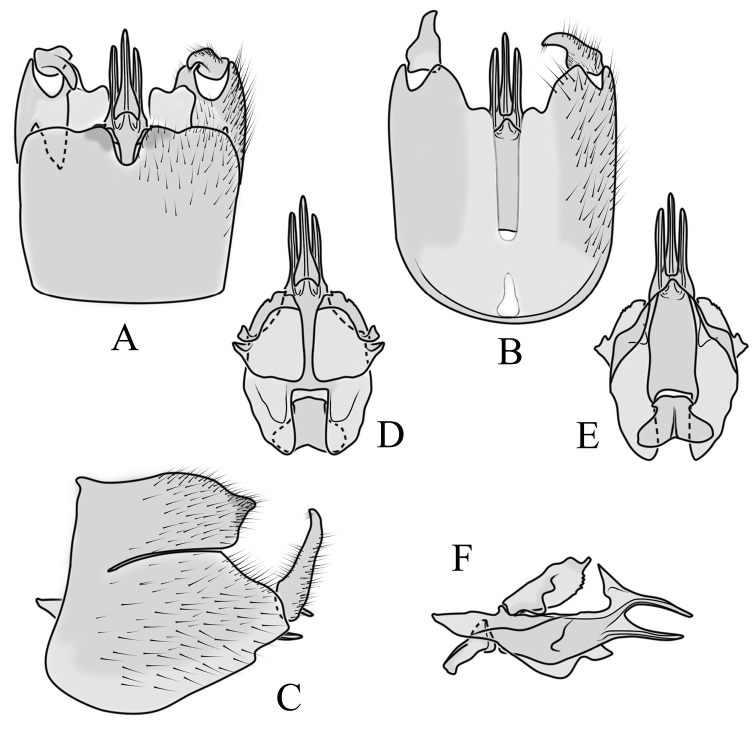
Male genital structures of *Phalacroceratipulina* Osten Sacken, 1865 **A** terminalia, dorsal view **B** terminalia, ventral view **C** terminalia, lateral view **D** aedeagus complex, dorsal view **E** aedeagus complex, ventral view **F** aedeagus complex, lateral view.

**Male terminalia** directed dorsally. Tergite 9 fused with gonocoxite and sternite 9 (Fig. 34C); caudal margin of tergite 9 medially with small, darker, outgrowths and with deep U-shaped notch between them (Fig. 34A); tip of median lobes rounded in dorsal view (Fig. 34A), triangular in in lateral view (Fig. 34C); tergite 9 without lateral lobes. Sternite 9 reduced to narrow band (Fig. 34B). Gonocoxite 1.2–1.3 × longer than tergite 9; ventral lobe very small membranous and indistinct (Fig. 34B, C); apical lobe indistinct; inner surface of gonocoxite sclerotised, with few hairs, forming a triangular sclerite. Gonostylus simple, tapering distally (Fig. 34A–C). Aedeagus complex 1.1–1.2 × longer than gonocoxite in lateral view (Fig. 34C); ejaculator apodeme medium sized, not covered by paramere in lateral view (Fig. 34F); interbase seems flat and wide, both dorsal and lateral view (Fig. 34D, F); interbase with small tooth apically, directed inward in dorsal view; caudal margin with additional notches, which seems teeth in lateral view; median lobe between interbases absent or indistinct; parameres fused ventrally, and forming wide plate, as wide as interbases together (Fig. 34D, E); aedeagus trifid, lateral branches straight, shorten than median tube (Fig. 34D–F); median branch longer than lateral branches, situated dorsally to lateral branches, with a bifid (visible dorsally or caudally), prominent outgrowth; directed dorsally, slightly backward (Fig. 34F); tips of branch tapering distally; sperm ducts branching from wide elongation of sperm pump; branching area slightly dark (Fig. 34D–F).

**Figure 35.**
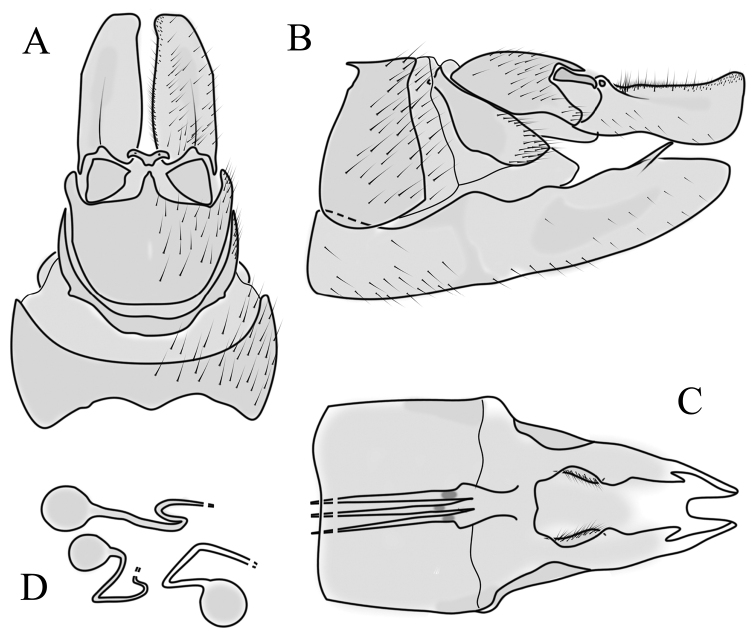
Female genital structures of *Phalacroceratipulina* Osten Sacken, 1865 **A** terminalia, dorsal view **B** terminalia, lateral view **C** sternite 8, hypogynial valve, and sperm ducts, inner dorsal view **D** spermathecae.

**Female terminalia**: Tergite 8 posterior part membranous, with few hairs, but not divided medially (Fig. 35A); wider than tergite 9 in lateral view (Fig. 35B). Tergite 9 directed caudally, lateral corner triangular. Triangular sclerite large, fused with tergite 10 uncertain; tergite 10 with small, less separated lateral lobe (Fig. 35B). Cercus short, widening apically, with blunt tip; dorsal margin with rugged margin, formed by small outgrowths (Fig. 35A, B). Sternite 8 and hypogynial valve wide and long, dorsal margin close to tip with tooth-like lobe, directed caudally (Fig. 35B, C). Common spermathecal duct present; sperm ducts simple, with distinct darkened area after branching, evenly tapering proximally (Fig. 35C); three spermathecae rounded, with very long and irregularly curved duct (Fig. 35D).

##### Distribution.

Canada, USA (Wisconsin to Ontario and Newfoundland, south to Virginia) (Oosterbroek 2021).

#### ﻿ Triogma kuwanai

Taxon classificationAnimaliaDipteraCylindrotomidae

(Alexander, 1913)

7BB10EA1-0CEB-5BFB-8EB0-B5B6B534A4A0

**Figure 36.**
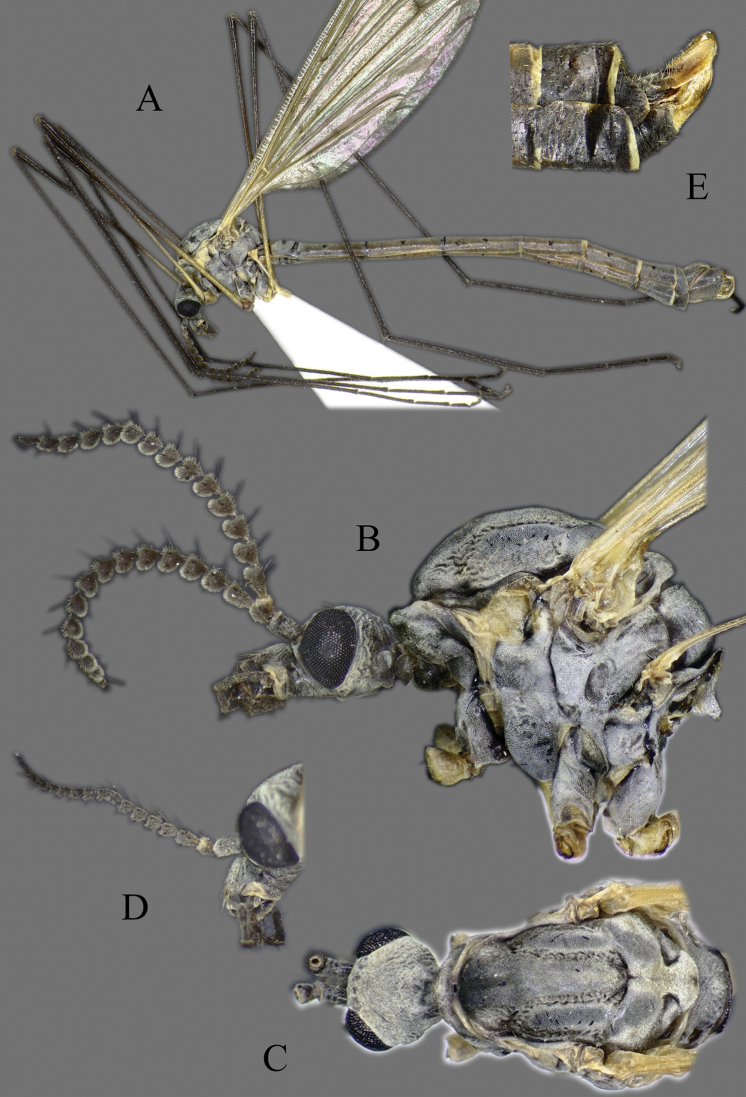
*Triogmakuwanai* (Alexander, 1913) **A** habitus of male, lateral view (colouration of wings is artefact) **B** head and thorax of male, lateral view **C** head and thorax, dorsal view **D** head of female, lateral view **E** female terminalia, lateral view.

**Figure 37.**
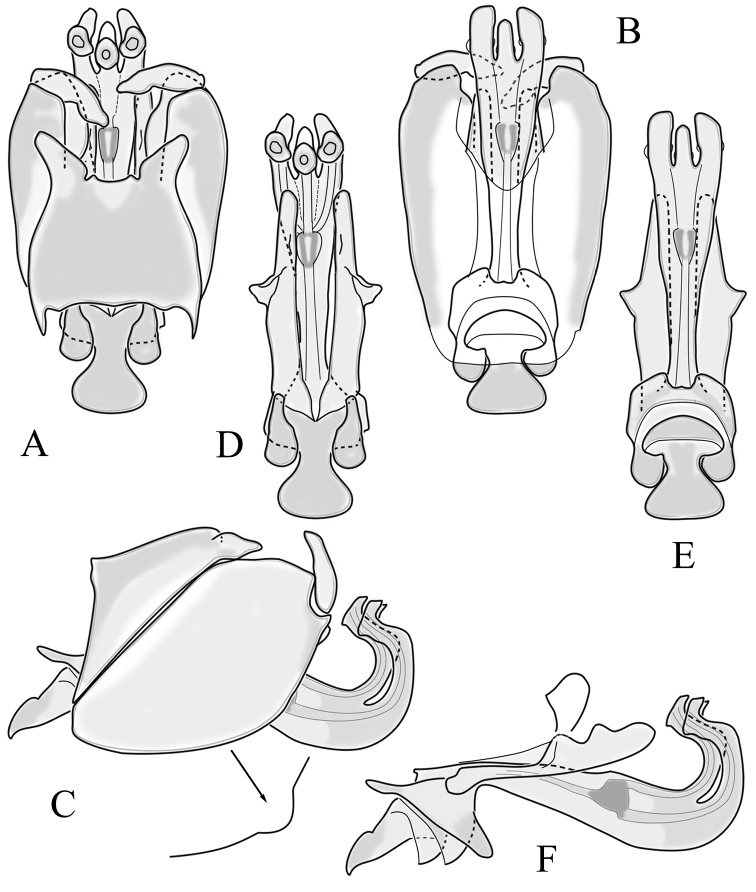
Male genital structures of *Triogmakuwanai* (Alexander, 1913) **A** terminalia, dorsal view **B** terminalia, ventral view **C** terminalia, lateral view, arrow indicating shape variability of gonocoxite margin **D** aedeagus complex, dorsal view **E** aedeagus complex, ventral view **F** aedeagus complex, lateral view.

**Figure 38.**
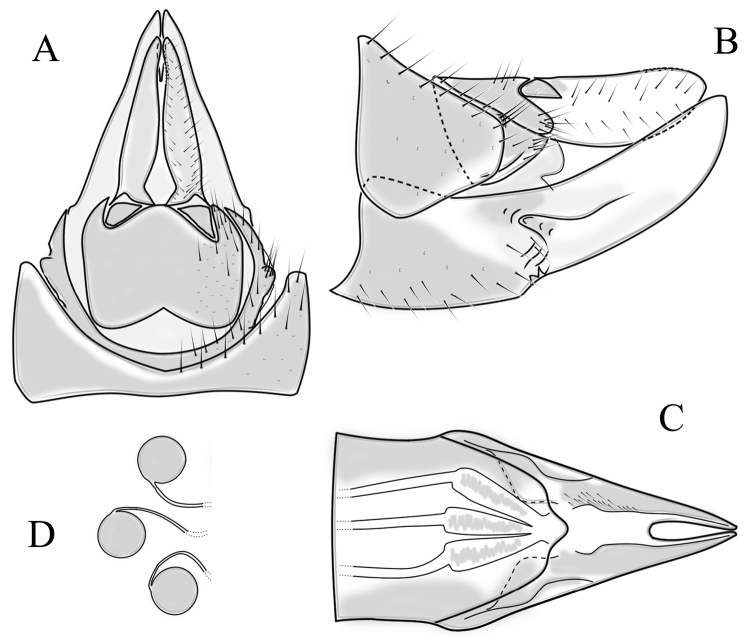
Female genital structures of *Triogmakuwanai* (Alexander, 1913) **A** terminalia, dorsal view **B** terminalia, lateral view **C** sternite 8, hypogynial valve, genital fork, and sperm ducts, inner dorsal view **D** spermathecae.

Figs 4G , 5G, H , 31B , 36 , 37 , 38

Triogma kuwanai limbinervis Alexander, 1953, syn. nov.
Triogma nimbipennis Alexander, 1941, syn. nov.
Liogma kuwanai in Alexander 1913: 294–295, 321–322: illustration, original description; Alexander 1920: 15–16: female description.
Triogma kuwanai in Alexander 1928: 12: distribution, illustrations, comb. nov.; Esaki 1950: illustration; Alexander 1953a: 56: faunistic records; in Takahashi 1960: 81: distribution; Nakamura 2001: 23–29: identification key, illustration, distribution, faunistic records; Nakamura 2005: 685: illustration; Oosterbroek 2020 (since 2018): taxonomic status. Imada 2020: biology and ecology of larvae.
Triogma kuwanai kuwanai in Ishida 1955: 76–77: distribution; Sidorenko 1999: 68–70: identification key, illustration, distribution; Paramonov 2006: 888–889: identification key, illustration, distribution; Nakamura 2014: 54: distribution; Kato and Suzuki 2017: 16: faunistic records, distribution.
Triogma kuwanai limbinervis ssp. n. in Alexander 1953a: 56–57: original description, illustration; Alexander, 1953b: 77: distribution; Takahashi 1960: 82: distribution; Sidorenko 1999: 68–70: identification key, illustration, distribution; Paramonov 2006: 888–889: identification key, distribution; Nakamura 2014: 54: distribution.
Triogma limbinervis in Oosterbroek 2020 (since 2018): taxonomic status.
Triogma nimbipennis in Alexander 1941: 407–408: original description, illustration, comparison; Yang 1991: information about type material; Sinclair and Dorchin 2010: 80: information about type material; Alexander and Alexander 1973: 69: catalogue, distribution; Oosterbroek 2020: taxonomic status.

##### Type material examined.

*Triogmakuwanailimbinervis* Alexander, 1953: ***Paratype***: **Japan** • ♀; Kochi, Tosa, Nisikawa, Mt. Yanase; alt. 800 m; 4 May. 1951; R. Takahashi leg.; USNM.

*Triogmanimbipennis* Alexander, 1941: ***Paratypes*: China** • ♂; Kuatun (Guadun), Fukien (Fukijen); 2500–3000 m; 23 Apr. 1938; • 2 ♀; same locality; 27 Apr. 1938 – 28 Apr. 1938; • 1 ♀; same locality; 27 Apr. 1938; Klapperich leg.; ZFMK.

##### Non-type material examined.

*Triogmakuwanaikuwanai* (Alexander, 1913): **Japan** • 1 ♂; Aomori, Hirosaki, Koguriyama, Inekari River; 40.53658°N, 140.48701°E; alt. 170 m; 24 May. 2013; • 1 ♂; same locality; 25 May. 2013; • 1 ♀; same locality; 28 May. 2013; D. Kato leg.; BLKU. • 2 ♂; Aomori, Nakadomari, Osawanai, Osawanai Pond; 40.94641°N, 140.46231°E; alt. 35 m; 15 May. 2014; D. Kato leg.; BLKU. • 1 ♂; Aomori, Nishimeyamura, Hirasawa River; 40.48729°N, 140.31335°E; alt. 710 m; 4 Jun. 2013; D. Kato leg.; BLKU. • 1 ♂; Aomori, Towada, Okuse, Tsutanuma Path; 40.59084°N, 140.95705°E; alt. 468 m; 23 May. 2014; D. Kato leg.; BLKU. • 1 ♂; Ehime, Iyo, Mt. Saragamine; 33.72°N, 132.89°E, 8 May. 1949; M. Miyatake leg.; EUMJ. • 1 ♀; Ehime, Komi, Yanadani; 33.55°N, 133.01°E; 6 May. 1994 – 8 May. 1994; Ohbayashi, Nishino, Okada le.; EUMJ. • 1 ♀; Ehime, Matsuyama, Misaka-toge; 33.71°N, 132.85°E; 3 May. 1951; Yano T. leg.; EUMJ. • 1, sex unknown; Ehime, Matsuyama, Sugitate; 33.84°N, 132.79°E; 8 Ap. 1950; M. Miyatake leg.; EUMJ. • 1 ♀; Ehime, ?Matsuyama, Shichidori; 33.84°N, 132.79°E; 3 May. 1952; Ide leg.; EUMJ. • 1 ♂; Ehime, Saijo, spring and mosses rocks; 33.75504°N, 133.15377°E; alt. 1480 m; 5 Jun. 2019; L.-P. Kolcsár leg.; CKLP. • 1 ♂; Fukuoka, Fukuoka, Sawara-ku, Itaya, Mt. Sefuri; 33.43811°N, 130.36673°E; alt. 970 m; 2 May. 2015; D. Kato leg.; BLKU. • 1 ♂; Ishikawa, Hakusan, near to Hakusan National Park; 36.25869°N, 136.72558°E; 678 m; 27 May. 2015; M. Kato leg.; CYI. • 1 ♂, 1 ♀; Nagano, Ueda, Sanada-machi, Irikaruizawa; 36.47441°N, 138.25481°E; alt. 777 m; 16 May. 2012; D. Kato leg.; BLKU. • 1 ♂; Oita, Kokonoe, Tano; 33.11621°N, 131.23541°E; alt. 1150 m; 7 May. 2016; D. Kato leg.; BLKU. • 1 ♂; Okayama, Maniwa, Hiruzen-Kamifukuda, Nawashirodani-gawa River; 34.08837°N, 133.87994°E; alt. 600 m; 30 Apr. 2016; D. Kato leg.; BLKU. • 2 ♂, 1 ♀; Saga, Karatsu, Kyuragi-Hirano, Mt. Sakurei; 33.35701°N, 130.07038°E; alt. 862 m; 26 Apr. 2015; D. Kato leg.; BLKU. • 1 ♀; Saitama, Saitama; 35.88°N, 139.26°E; 29 May. 1919; R. Takahashi leg.; USNM. • 2 ♂, 1 ♀; Shizuoka, Shizuoka, Ikawa-touge; 35.24094°N, 138.28156°E; alt. 1471 m; 10 May. 2015; M. Kato leg.; •1 ♂; same locality; 18 May. 2016; Y. Imada leg.; CYI. • 1 ♂; Tokushima, Awa, Mt. Tsurugi; 33.87°N, 134.11°E; 31 May. 1950; Issiki-Ito leg.; USNM. • 2 ♂; Tokushima, Higashimiyoshi, Higashiyama, Ogawadani River; 34.08837°N, 133.87994°E; alt. 340 m; 21 Apr. 2014; D. Kato leg.; BLKU. • 1 ♂; Tokushima, Miyoshi, Higashiiya-Ochiai, around Matsuogawa Dam; 33.96478°N, 133.93908°E; alt. 900 m; 30 Apr. 2016; D. Kato leg.; BLKU. • 1, sex unknown; Tokyo, Meguro; 35.62°N, 139.7°E; 8 Apr. 1919; R. Takahashi leg.; USNM. • 1 ♂; Tokyo, Mt. Mitake; 35.78°N, 139.14°E; 10 May. 1931; B. Oda leg.; USNM. • 1, sex unknown; Tokyo, Tokyo; 35.67°N, 139.69°E; 8 Apr. 1930; R. Takahashi leg.; USNM. • 1 ♂; Tottori, Kurayoshi, Sekigane-cho-Nozoe, Mt. Karasuga; 35.35352°N, 133.58577°E; alt. 1000 m; 17 May. 2015; D. Kato leg.; BLKU.

*Triogmakuwanailimbinervis* Alexander: **Japan** • 7 ♂ 1 ♀; Ehime, Matsuyama, small ruderal streem; 33.86328°N, 132.77157°E; alt. 125 m; 31 Mar. 2019; L.-P. Kolcsár leg.; CKLP. • 2 ♀; Ehime, Matsuyama, ruderal forest and orange plantation; 33.86041°N, 132.76552°E; alt. 84 m; 6 Apr. 2019; L.-P. Kolcsár leg.; CKLP.

##### Redescription.

**Head.** Rugose; ground colouration dark brown to black, with very intense greyish pubescence (Fig. 36B, C). Rostrum moderately long, with few short hairs; palpus greyish black, five segmented; last segment 1.4–1.6 × longer than penultimate in male, 1.2–1.3 × in female. Scape cylindrical, rugose, ~ 2 × as long as pedicel; pedicel ovate; flagellum 14 segmented monochrome greyish black (Figs 4G, 36B, D). Male flagellomeres, except ultimate, expanded ventrally, covered with dense whitish grey sensilla, denser ventrally; ultimate flagellomere cylindrical, with several sensilla (Figs 4G, 36B); female flagellomeres 1–5 or 6 extended ventrally, remaining flagellomeres fusiform to cylindrical; flagellomeres 1–10 or 11 bearing sparse whitish grey sensilla mostly on ventral side (Figs 4G, 36E). Flagellomere with two long verticels on dorsal surface, two short on lateral face, and two short on ventral side; first flagellomere always bearing additional 1–4 verticels.

**Thorax.** Ground colouration dark brown to black, with very dense and intensive grey pruinosity, thorax appearing grey (Fig. 36A–C). Pleural area, wing base, and halter yellow to yellowish brown (Fig. 36B). Anterior part of mesonotum with rugose sutures (Fig. 36C); lateral margin of scutum rugose (Fig. 36A, B). Anterior half of mediotergite rugose (Fig. 36B). Katepisternum and metakatepisternum weakly rugose (Fig. 36B). Trochanter yellow to pale brown; femur gradually darkening apically, basally yellowish, apically black; tibia and tarsus uniformly black (Fig. 36A). Wing hyaline, tinged with pale brown; membrane with interference patterns, visible with dark background (Fig. 36A); pterostigma brown; veins yellow at base of wing, apically brownish; three branches of M reaching wing margin; M_1_ at same level as M_1+2_, cell a2 less than 6 × longer than wide (Fig. 5G, H); small, weakly infuscate areas around base and fork of Rs, at crossvein r-m (if present), at base of M_1+2_, at crossvein m-cu, M_2_, and crossvein m-m. Note: the early spring specimens from Shikoku Island have more intensive wing pattern (Fig. 5H), than the later spring specimens, or specimens collected in the other part of Japan (Fig. 5G). The pattern is more intensive in the living specimens, less prominent in the dead ones, and became paler after time.

**Abdomen.** Grey with reddish tinge, caudal half of tergites and sternites 8 and 9 darker. Abdominal plaques (external remnants of attachment sites of muscles in the pupa) shiny, punctuated (Fig. 36A).

**Male terminalia**: Reddish grey, directed caudally (Fig. 36A). Tergite 9 fused with gonocoxite at base, fusion suture present (Fig. 37C); tergite 9 with laterally directed, ear-like lobes in dorsal view (Fig. 37A), triangular or bird-head-shaped laterally (Fig. 37C); additional two very small, triangular lobe on posterior margin of tergite 9. Sternite 9 fully membranous (Fig. 37B). Gonocoxite large 1.5–1.6 × longer than tergite 9, without evident ventral lobe (Fig. 37B, C), small protuberance on ventral margin of gonocoxite in some specimen rarely present (Fig. 37C see arrow). Gonostylus simple, generally tapering to distal end (Fig. 37A, C). Aedeagus complex very large, 1.5–1.7 × longer than gonocoxite (Fig. 37D–F); ejaculatory apodeme and sperm pump large, not covered by paramere in lateral view (Fig. 37D–F); interbase simple, tip rounded or sharp, with small lobe dorsally in lateral view (Fig. 37F); dorsal lobe between interbases, membranous, bubble-like; sperm ducts branching from elongation of sperm pump, area darkened (Fig. 37F); aedeagus 2 × as wide as interbase in lateral view; directed ventrally then turned dorsally, almost turning back anteriorly (Fig. 37F); trifid, medial branch shorter than lateral branches (Fig. 37D–E); tips of branches widened and flattened (Fig. 37D–F).

**Female terminalia**: Cercus and hypopygial valve pale brown (Fig. 36E). Tergite 8, ~ 1.5 × larger than tergite 9 in lateral view (Fig. 38B); very broad in dorsal view, not divided medially (Fig. 38A). Tergite 9 triangular in lateral view, with a small round lobe at middle, with few longer setae (Fig. 38A). Triangular sclerites of tergite 10 variable in size (Fig. 38A), in some specimens partly fused with tergite 10. Cercus simple, tip rounded or weakly pointed; dorsal margin weakly rugged, formed by small pyramidal teeth (Fig. 38A, B). Hypogynial valve long, blade-like, longer than cercus; with pit at base, holding lateral lobes of male tergite 9 during copulation (Fig. 38B). Common spermathecal duct short; spermathecal ducts wide, carrot-shaped, suddenly narrow; inner wall rugged (Fig. 38C); three round spermathecae, with very narrow duct (Fig. 38D).

##### Distribution.

Japan (Fig. 31B) (Hokkaido I, Honshu I, Shikoku I, and Kyushu I) (Oosterbroek 2021). Distribution records of *Triogmakuwanailimbinervis* (Shikoku I) and *T.nimbipennis* transferred to *Triogmakuwanai* (China: Zhejiang and Fujian).

##### Comments.

Alexander (1953a) described the subspecies, *Triogmakuwanailimbinervis*, from Shikoku Island, of which wing markings in some individuals are more conspicuous than those of *T.k.kuwanai*. In the original description, Alexander noted, “there is evidence of mergence with the typical form – *Triogmakuwanaikuwanai* –, where the wings are unpatterned or virtually so” (Alexander 1953a). *Triogmak.limbinervis* has been referred to as the *T.limbinervis* in the CCW (since 2018) due to its sympatric occurrence with *Triogmakuwanai* in Shikoku Island. This study suggests that wing markings, the diagnostic character of *T.k.limbinervis* (Fig. 5H), appear only in early spring specimens from Shikoku and Kyushu Islands, but do not after early spring in later specimens. It is also observed that wing marking turns paler over time. Some specimens from northern Honshu occasionally have pale wing markings, as well, suggesting variation. These two subspecies do not significantly differ in terms of male and female terminalia, and were not distinguished as different by the barcode sequences. *Triogmak.limbinervis* syn. nov. is therefore synonymised with *Triogmakuwanai*. Another closely related species *T.nimbipennis* Alexander, 1941, was described from China (Zhejiang and Fujian) (Alexander 1941). This species is quite similar to *T.kuwanai*, and shows a subtle difference in the colour tint: particularly, the wings of *T.nimbipennis* are darker than those of *T.kuwanai*. Alexander (1941) mentioned that *T.nimbipennis* can be considered as a subspecies of *T.kuwanai*. After the morphological comparison of the type specimens of *T.nimbipennis* with *T.kuwanai*, the two species were found not to differ in genital structure, and so *T.nimbipennis* syn. nov. is proposed as a junior synonym of *Triogmakuwanai*.

#### ﻿ Triogma trisulcata

Taxon classificationAnimaliaDipteraCylindrotomidae

(Schummel, 1829)

D1B2E355-66A6-5491-AC5B-46E84C0AD7F6

Figs 39 , 40

Triogma pulla (Meigen, 1830)

##### Non-type material examined.

**Russia** • 1 ♂, 1 ♀; Leningrad Oblast, Luzhsky District, around Luga City; 58.74°N, 29.85°E; alt. 40 m; 5 Jun. 1954; A.A. Stackelberg leg.; CKLP. **United Kingdom** • 2 ♂; Birmingham, Sutton Park, Longmoor Valley; 52.5635°N, 1.8633° W; alt. 125 m; 30 Apr. 2019; P. Boardman leg.; CKLP.

##### Supplementary description.

**Figure 39.**
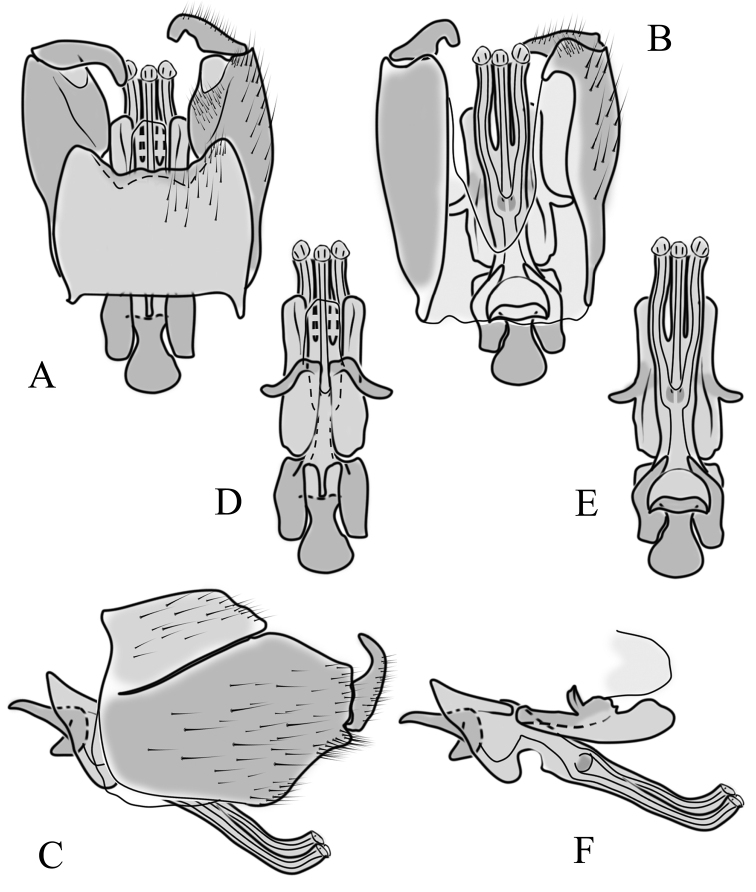
Male genital structures of *Triogmatrisulcata* (Schummel, 1829) **A** terminalia, dorsal view **B** terminalia, ventral view **C** terminalia, lateral view **D** aedeagus complex, dorsal view **E** aedeagus complex, ventral view **F** aedeagus complex, lateral view.

**Male terminalia.** Directed caudally. Tergite 9 fused with gonocoxites at base (Fig. 39C). Tergite 9 lateral parts weakly produced, triangular (Fig. 39A, C); posterior margin bent back under tergite 9, forming W-shaped plate (Fig. 39A). Sternite 9 fully membranous (Fig. 39B). Gonocoxite large ~ 1.5–1.6 × longer than tergite 9, without evident ventral or apical lobe (Fig. 39B, C); inner surface hairy. Gonostylus simple, narrowing to end (Fig. 39A, C). Aedeagus complex 1.4 × longer than gonocoxite (Fig. 39C); ejaculatory apodeme medium size, not covered by paramere in lateral view (Fig. 39F); interbase weakly curve dorsally, with small notch at tip in lateral view (Fig. 39F); dorsal lobe between interbases, membranous, bubble-like; sperm ducts branching from elongation of sperm pump, branching area darker (Fig. 39E, F); aedeagus trifid, as wide as interbase at mid-length in lateral view, aedeagus directed ventrally, just tip turning back dorsally (Fig. 39F); medial branch shorter than lateral branches (Fig. 39D–F).

**Figure 40.**
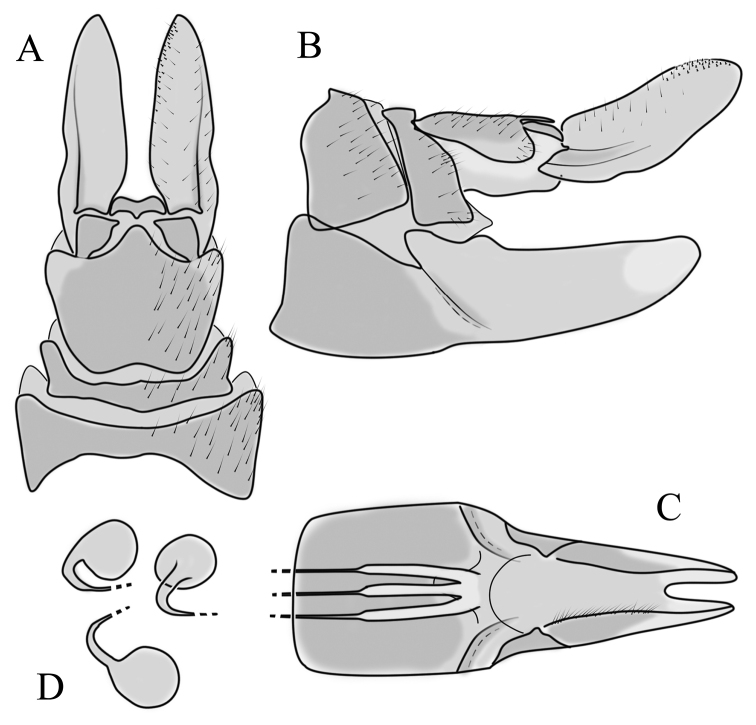
Female genital structures of *Triogmatrisulcata* (Schummel, 1829) **A** terminalia, dorsal view **B** terminalia, lateral view **C** sternite 8, hypogynial valve, and sperm ducts, inner dorsal view **D** spermathecae.

**Female terminalia**: Tergite 8, ~ 2 × wider than tergite 9 in lateral view (Fig. 40B); not divided medially, posterior part partly membranous with a few hairs (Fig. 40A). Tergite 9 rectangular in lateral view (Fig. 40B). Triangular of tergite 10 large, fused with tergite 10 (Fig. 40A). Cercus simple, with distinct rugged area at tip; formed by short pyramid teeth (Fig. 40A, B); ventral margin with small, rounded notch at 1/3 of length. Hypogynial valve long, blade-like, shorter than cercus; with transverse ditch at base, holding lateral lobes of male tergite 9 during copulation (Fig. 40B). Common spermathecal duct short; spermathecal ducts carrot-shaped, without clear pattern; suddenly narrow (Fig. 40C); three spermathecae large, irregularly spherical, with comparably long and curved duct (Fig. 40D).

##### Distribution.

Palearctic species, with a wide distribution range in Europe, except the southern parts. Previously reported from Austria, Belgium, Czech Republic, Denmark, Estonia, Finland, France, Germany, Great Britain, Hungary, Italy, Latvia, Lithuania, Netherlands, Norway, Poland, Romania, Russia (North and Central European Russia), Slovakia, Sweden, and Switzerland. It was reported from Eastern Palearctic, but so far only from East Siberia (Irkutsk Oblast), Russia (Oosterbroek 2021).

## Supplementary Material

XML Treatment for Cylindrotoma distinctissima

XML Treatment for Cylindrotoma americana

XML Treatment for Cylindrotoma nigriventris

XML Treatment for Diogma caudata

XML Treatment for Diogma glabrata

XML Treatment for Diogma dmitrii

XML Treatment for Liogma brevipecten

XML Treatment for Liogma mikado

XML Treatment for Liogma nodicornis

XML Treatment for Liogma serraticornis

XML Treatment for Phalacrocera replicata

XML Treatment for Phalacrocera tipulina

XML Treatment for Triogma kuwanai

XML Treatment for Triogma trisulcata
